# Supplementary material for: Effective coverage of nutrition interventions across the continuum of care in Bangladesh: insights from nationwide cross-sectional household and health facility surveys
Source: BMJ Open. 2021 Jan 20;11(1):e040109. doi: 10.1136/bmjopen-2020-040109 (PMC7818835; doi:10.1136/bmjopen-2020-040109)
Supplement: Supplementary data [file bmjopen-2020-040109supp001.pdf]

Supplemental Table 1. Harmonized facility categories and reported categories in SPA and DHS

Harmonized facility category	SPA facility category	DHS facility category
District and upazila public facilities	District hospital (DH)<br>Upazila health complex (UHC)<br>Maternal and child welfare center (MCWC)	District hospital (DH)<br>Upazila health complex (UHC)<br>Maternal and child welfare center (MCWC)
Union level public facilities	Union health and family welfare center (UHFWC)/(UHFWC upgraded)<br>Union subcenter/rural dispensary	Upazila health & family welfare center<br>Satellite clinic/epi outreach
Public community clinic	Public community clinic	Public community clinic<br>Other public sectors
NGO clinic/hospital	NGO clinic/hospital	NGO static clinic<br>NGO satellite clinic<br>Other NGO clinic
Private clinic/hospital	Private clinic/hospital	Private clinic/hospital<br>Private medical<br>Traditional/Qualified doctor

Supplemental Table 2: Percentage of health facilities with structural input items, by division

		Barisal	Chittagong	Dhaka	Khulna	Rajshahi	Rangpur	Sylhet
**Antenatal care**								
**Staff**	Staff with any training on ANC	38.55	57.24	57.8	44.27	35.87	39.45	54.35
**Guideline**	National guidelines for ANC	56.26	38.49	39.93	58.47	46.86	70.65	60.09
	Visual aids for client education	69.80	63.78	57.09	72.27	67.74	72.19	91.51
**Functional equipment**	Adult weighing scale	88.74	89.73	72.93	91.45	87.21	93.23	90.38
	Tape measure for fundal height	41.18	27.51	33.42	40.77	32.44	50.37	36.35
	Blood pressure apparatus	87.03	86.88	90.67	94.51	92.03	97.85	88.96
	Stethoscope	89.75	86.32	92.10	96.73	93.69	96.06	87.98
	Fetal stethoscope	13.36	7.84	15.89	13.24	8.39	29.11	26.26
**Diagnostics capacity**	Hemoglobin	5.91	8.68	12.46	3.71	9.29	19.29	11.60
	Urine protein	8.28	15.84	20.06	11.35	18.58	26.71	18.58
**Medicine**	Iron tablets	41.31	50.18	64.42	64.36	73.60	65.79	42.02
	IFA tablets	93.74	80.16	73.44	83.17	89.06	90.34	86.82
**Birth care**								
**Staff**	Staff with any training on IMPACT	23.68	33.01	45.01	37.02	35.94	43.94	34.23
**Guideline**	Guidelines for BEmOC or CEmOC	32.66	26.00	25.83	21.19	10.84	56.25	20.46
**Functional equipment**	Infant scale	52.46	56.88	49.54	61.50	77.21	89.71	52.01
	Manual or digital BP apparatus	90.45	89.58	94.13	82.86	100.00	100.00	92.91
**Child growth monitoring**								
**Staff**	Staff with any training on growth monitoring	50.08	46.13	55.37	47.18	50.20	40.08	49.72
**Guideline**	Guidelines for growth monitoring	57.50	48.33	37.09	51.80	41.36	65.11	22.03
**Functional equipment**	Child scale (child or infant)	69.87	67.66	50.28	60.79	75.85	96.16	82.95
	Length or height board	70.34	49.76	50.48	56.34	66.41	69.23	72.63
	Tape for measuring head	32.28	24.25	23.24	30.86	27.20	43.00	50.57
	Growth chart	83.21	69.66	45.23	71.65	60.51	91.50	66.73
Readiness score for child growth monitoring								
**Sick child care**								
**Staff**	Staff with any training on IMCI	67.19	48.62	57.54	42.75	52.18	55.86	58.89
**Guideline**	Guidelines for IMCI	66.62	42.83	38.33	51.17	45.03	77.60	58.55
	IMCI chart booklet	64.33	40.94	49.47	34.79	55.25	84.87	57.20
	IMCI mother's cards (IMCI card)	43.90	32.20	31.08	25.35	32.13	83.08	38.93
	Other visual aids for teaching caretakers	64.14	46.00	43.75	53.46	60.44	80.14	71.73
**Functional equipment**	Child scale (child or infant)	66.12	57.20	40.63	64.15	64.93	84.61	79.74
**Diagnostics capacity**	Hemoglobin	5.15	8.29	11.85	3.45	9.50	19.83	11.63
**Medicine**	ORS	71.97	63.52	61.01	51.95	74.91	69.94	52.35
	Albendazole/ Mebendazole	91.78	87.03	89.88	85.69	89.78	96.99	97.72
	Iron tablet	35.08	43.04	57.41	60.68	72.61	58.15	33.61
	Vitamin A	67.99	54.26	67.73	62.17	76.47	81.77	47.22
	Zinc tablet/ Zinc sulphate syrup	76.46	56.77	69.70	61.62	71.29	76.64	62.32

ANC: antenatal care, BEmOC: Basic Emergency Obstetric and Neonatal Care, CEmOC: Comprehensive Emergency Obstetric and Neonatal Care, IMCI: integrated management of childhood illness, IMPAC: integrated management of pregnancy and childbirth, ORS: Oral rehydration salts.

Supplemental Table 3: Percentage of health facilities with structural input items, by facility type

		DUPF	ULPF	PC	NGO	Private
**Antenatal care**						
**Staff**	Staff with any training on ANC	83.64	50.04	46.31	60.36	27.48
**Guideline**	National or other guidelines for ANC	70.33	47.82	48.16	72.95	24.70
	Visual aids for client education	79.25	68.52	65.47	78.34	45.94
**Equipment**	Adult weighing scale	91.47	82.20	85.67	94.15	83.82
	Tape measure for fundal height	60.76	35.40	32.67	63.31	52.64
	Blood pressure apparatus	99.12	90.74	90.30	99.14	94.67
	Stethoscope	98.94	91.02	91.18	99.15	97.97
	Fetal stethoscope	46.35	20.38	8.67	46.55	43.29
**Diagnostics capacity**	Hemoglobin	61.97	7.90	3.53	58.87	72.14
	Urine protein	63.89	12.02	12.34	68.63	68.12
**Medicine**	Iron tablets	69.37	62.22	57.81	73.95	57.60
	IFA tablets	88.23	82.69	84.79	82.66	62.35
**Birth care**						
**Staff**	Staff with any training on IMPACT	55.96	31.75	40.71	52.84	22.05
**Guideline**	Guidelines for BEmOC or CEmOC	40.50	26.09	27.42	27.94	11.29
**Equipment**	Infant scale	69.52	60.47	47.87	76.72	64.97
	Manual or digital BP apparatus	96.91	88.33	93.18	99.34	100.00
**Child growth monitoring**						
**Staff**	Staff with any training on growth monitoring	67.88	42.91	50.18	47.73	40.35
**Guideline**	Guidelines for growth monitoring	52.55	43.30	46.22	64.57	29.31
**Equipment**	Child scale (child or infant)	82.28	74.50	64.19	86.20	90.63
	Tape for measuring head	47.65	26.41	28.95	57.15	32.90
	Length or height board	71.77	52.26	60.09	71.51	57.32
	Growth chart	85.51	68.72	63.01	83.71	85.13
Readiness score for child growth monitoring						
**Sick child care**						
**Staff**	Staff with any training on IMCI	78.46	52.70	54.11	45.91	32.97
**Guideline**	Guidelines for IMCI	76.74	55.46	47.93	56.32	30.23
	IMCI chart booklet	78.06	59.78	49.35	64.58	35.14
	IMCI mother's cards (IMCI card)	62.65	50.15	33.78	52.56	24.58
	Other visual aids for teaching caretakers	65.70	54.39	55.88	68.49	41.49
**Equipment**	Child scale (child or infant)	84.76	66.10	55.77	80.08	77.59
**Diagnostics capacity**	Hemoglobin	62.53	8.18	3.78	58.33	68.26
**Medicine**	ORS	64.45	34.50	73.57	78.80	46.65
	Albendazole/ Mebendazole	75.39	87.03	93.31	83.53	79.06
	Iron tablet	64.20	53.69	52.45	73.53	59.43
	Vitamin A	58.72	29.90	80.73	70.19	53.41
	Zinc tablet/ Zinc sulphate syrup	65.12	34.36	79.26	73.28	80.94

DUPF: District and upazila public facilities, ULPF: Union level public facilities Public community clinic
ANC: antenatal care, BEmOC: Basic Emergency Obstetric and Neonatal Care, CEmOC: Comprehensive Emergency Obstetric and Neonatal Care, IMCI: integrated management of childhood illness, IMPAC: integrated management of pregnancy and childbirth, ORS: Oral rehydration salts.

Supplemental Figure 1: Place of ANC and child care by facility type, Bangladesh 2014

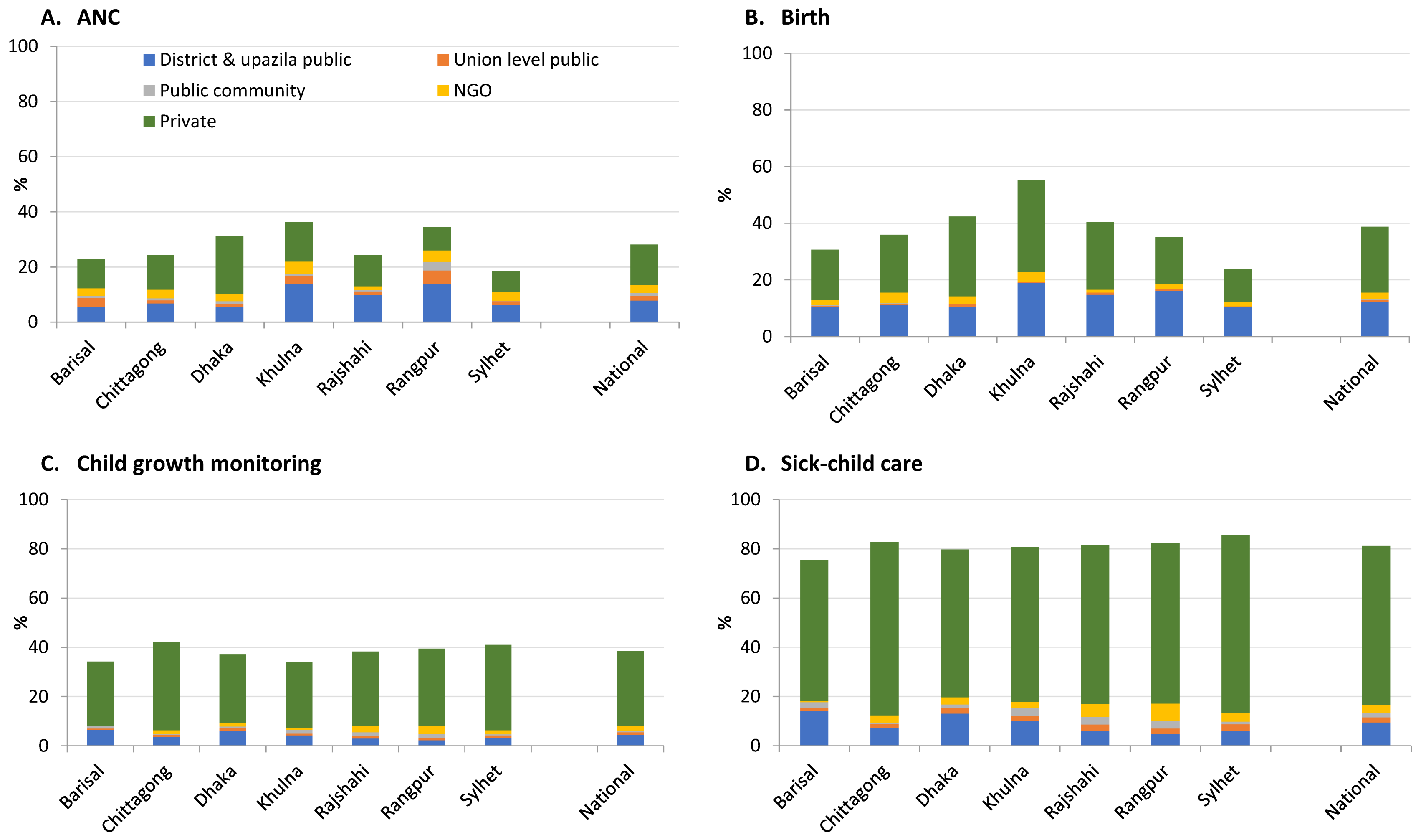

Source: DHS data

Supplemental Figure 2: Distribution of facilities with ANC and sick child care by facility type, Bangladesh 2014

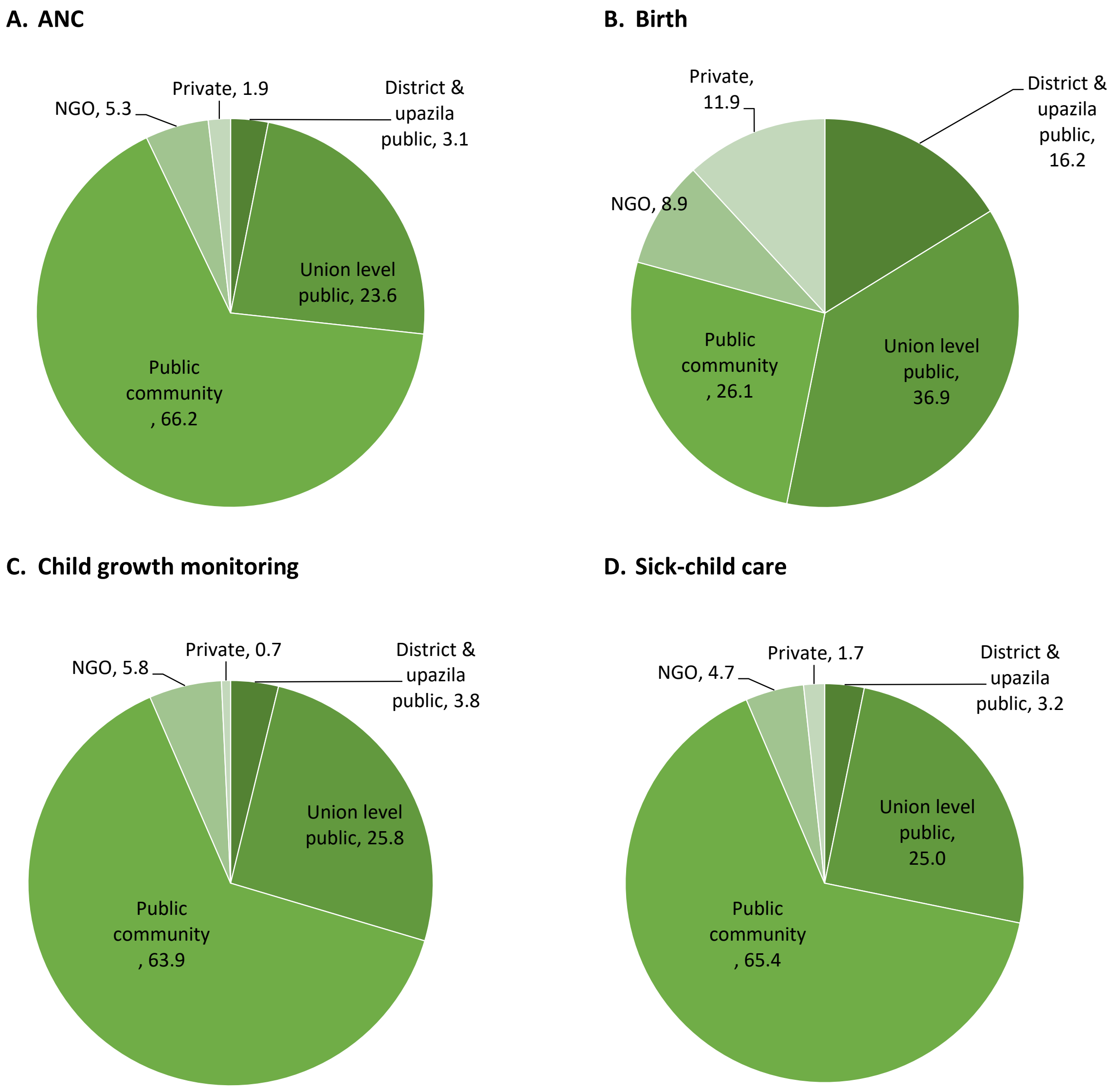

Source: SPA data
