# Supplementary material for: Effective coverage of nutrition interventions across the continuum of care in Bangladesh: insights from nationwide cross-sectional household and health facility surveys
Source: BMJ Open. 2021 Jan 20;11(1):e040109. doi: 10.1136/bmjopen-2020-040109 (PMC7818835; doi:10.1136/bmjopen-2020-040109)
Supplement: Author's manuscript [file bmjopen-2020-040109.draft_revisions.pdf]

BMJ Open

BMJ Open is committed to open peer review. As part of this commitment we make the peer review history of every article we publish publicly available.

When an article is published we post the peer reviewers' comments and the authors' responses online. We also post the versions of the paper that were used during peer review. These are the versions that the peer review comments apply to.

The versions of the paper that follow are the versions that were submitted during the peer review process. They are not the versions of record or the final published versions. They should not be cited or distributed as the published version of this manuscript.

BMJ Open is an open access journal and the full, final, typeset and author-corrected version of record of the manuscript is available on our site with no access controls, subscription charges or pay-per-view fees (http://bmjopen.bmj.com).

If you have any questions on BMJ Open's open peer review process please email info.bmjopen@bmj.com

BMJ Open

Effective coverage of nutrition interventions across the continuum of care: Insights from household and health facility data in Bangladesh

Journal:	*BMJ Open*
Manuscript ID	bmjopen-2020-040109
Article Type:	Original research
Date Submitted by the Author:	05-May-2020
Complete List of Authors:	Nguyen, Phuong Hong; International Food Policy Research Institute<br>Khương , Quỳnh Long ; Hanoi University of Public Health<br>Pramanik, Priyanjana ; International Food Policy Research Institute<br>Billah, Sk; ICDDRB, Maternal and Child Health Division<br>Menon, Purnima; International Food Policy Research Institute<br>Piwoz, Ellen; Bill and Melinda Gates Foundation,<br>Leslie, Hannah; Harvard TH Chan School of Public Health, Global Health and Population
Keywords:	EPIDEMIOLOGY, International health services < HEALTH SERVICES ADMINISTRATION & MANAGEMENT, Quality in health care < HEALTH SERVICES ADMINISTRATION & MANAGEMENT, Public health < INFECTIOUS DISEASES, NUTRITION & DIETETICS

SCHOLARONE™
Manuscripts

I, the Submitting Author has the right to grant and does grant on behalf of all authors of the Work (as defined in the below author licence), an exclusive licence and/or a non-exclusive licence for contributions from authors who are: i) UK Crown employees; ii) where BMJ has agreed a CC-BY licence shall apply, and/or iii) in accordance with the terms applicable for US Federal Government officers or employees acting as part of their official duties; on a worldwide, perpetual, irrevocable, royalty-free basis to BMJ Publishing Group Ltd ("BMJ") its licensees and where the relevant Journal is co-owned by BMJ to the co-owners of the Journal, to publish the Work in this journal and any other BMJ products and to exploit all rights, as set out in our licence.

The Submitting Author accepts and understands that any supply made under these terms is made by BMJ to the Submitting Author unless you are acting as an employee on behalf of your employer or a postgraduate student of an affiliated institution which is paying any applicable article publishing charge ("APC") for Open Access articles. Where the Submitting Author wishes to make the Work available on an Open Access basis (and intends to pay the relevant APC), the terms of reuse of such Open Access shall be governed by a Creative Commons licence – details of these licences and which Creative Commons licence will apply to this Work are set out in our licence referred to above.

Other than as permitted in any relevant BMJ Author's Self Archiving Policies, I confirm this Work has not been accepted for publication elsewhere, is not being considered for publication elsewhere and does not duplicate material already published. I confirm all authors consent to publication of this Work and authorise the granting of this licence.

For peer review only

Effective coverage of nutrition interventions across the continuum of care: Insights from household and health facility data in Bangladesh

Authors:

Phuong Hong Nguyen[1*], Long Quynh Khuong[2], Priyanjana Pramanik[1], Sk Masum Billah[3 4], Purnima Menon[1], Ellen Piwoz[5], and Hannah H Leslie[6]

Author affiliations:

[1]International Food Policy Research Institute, Washington, DC, USA; [2]Hanoi University of Public Health, Hanoi, Viet Nam; [3]Maternal and Child Health Division, ICDDR, B, Dhaka, Bangladesh; [4]The University of Sydney, Faculty of Medicine and Health, Sydney School of Public Health, New South Wales, Australia [5]Bill & Melinda Gates Foundation; [6]Harvard T.H. Chan School of Public Health, Boston, MA, USA

***Corresponding author:**

Phuong Hong Nguyen, Poverty, Health and Nutrition Division, International Food Policy Research Institute, 2005 I Street, NW, Washington DC, 20005. Phone: 202-826-4088. Email: P.H.Nguyen@cgiar.org

Word count: 4,101 (from introduction to conclusion, not including tables)

Abbreviations

ANC : Antenatal care

BEmOC : Basic Emergency Obstetric and Neonatal Care

CEmOC : Comprehensive Emergency Obstetric and Neonatal Care

DHS : Demographic and Health Surveys

DUPF : District and upazila public facilities

For peer review only - http://bmjopen.bmj.com/site/about/guidelines.xhtml

23	IFA	: Iron folic acid
24	IMCI	: Integrated management of childhood illness
25	IMPAC	: Integrated management of pregnancy and childbirth
26	LMIC	: Low- and middle-income countries
27	MUAC	: Mid-upper arm circumference
28	NGO	: Non-governmental organization
29	ORS	: Oral rehydration salts
30	PC	: Public community clinic
31	Pp	: Percentage points
32	SES	: Socio-economic status
33	SPA	: Service Provision Assessment
34	ULPF	: Union level public facilities
35	WHO	: World Health Organization

For peer review only

For peer review only - http://bmjopen.bmj.com/site/about/guidelines.xhtml

ABSTRACT

Introduction: Improving the impact of nutrition interventions requires adequate measurement of both reach and quality of interventions, but limited evidence exists on advancing coverage measurement. We adjust contact-based coverage estimates, taking into consideration the inputs required to deliver quality nutrition services, to calculate effective coverage of nutrition interventions across the continuum of care in Bangladesh.

Methods: We used data from the 2014 Bangladesh Demographic and Health Surveys to assess use of maternal and child health services and the 2014 Service Provision Assessment to determine facility readiness to deliver nutrition interventions. Service readiness was computed as the mean availability of nutrition-specific inputs, capturing human resources and training, equipment, diagnostics, and medicines. Contact coverage was combined with service readiness to create a measure of input-adjusted nutrition coverage at the national and regional levels, across place of residence, and by maternal education and household socio-economic quintiles.

Results: Contact coverage varied from 28% for attending at least 4 ANC visits to 38% for institutional delivery, 35% for child growth monitoring and 81% for sick-child care. Facilities demonstrated incomplete readiness for nutrition services, ranging from 45 to 63% across services. Nutrition input-adjusted coverage was 18% for ANC, 25% for institutional delivery, 21% for child growth monitoring and 51% for sick-child care. Input-adjusted coverage varied by 8-24 percentage points (pp) between regions within the country. Inequalities in input-adjusted coverage were large during ANC and institutional delivery (13-18pp between urban and rural areas, 14-17pp between low and high education, and 27-37pp between highest and lowest wealth quintiles), and less variable for sick-child care (< 2pp).

For peer review only - http://bmjopen.bmj.com/site/about/guidelines.xhtml

Conclusion: Nutrition input-adjusted coverage was suboptimal and varied sub-nationally and across the continuum of care in Bangladesh. Special efforts are needed to improve the reach as well as the quality of health and nutrition services to achieve the Sustainable Development Goals.

Keywords: Bangladesh, facility readiness, input-adjusted coverage, nutrition, effective coverage

For peer review only

For peer review only - http://bmjopen.bmj.com/site/about/guidelines.xhtml

Strengths and limitations of this study

- To bridge the gap in limited evidence on advancing coverage measurement, this study develops and applies nutrition-related metrics of effective coverage across the continuum of care by incorporating service readiness of nutrition interventions into coverage estimates of ANC and post-delivery care.
- Our study draws on nationally representative population and health system data, applies recently validated methods for effective coverage estimation, and is one of the few analyses to extend effective coverage assessments to quantify inequities within the population.
- Our study provides insight into health system challenges in Bangladesh and indicates potential priorities for improvement, including special investment in reaching underserved populations with key services, while also ensuring adequate quality of services.
- The exclusion of small private clinics hospitals with less than 20 beds could lead to an overestimation of quality for private sector users as larger facilities tend to have higher readiness than smaller ones. Contact coverage could be influenced by recall bias. The assumption of no systematic difference in facility readiness within region by the education or socioeconomic stratifies may affect inequality estimates.

For peer review only - http://bmjopen.bmj.com/site/about/guidelines.xhtml

INTRODUCTION

Global evidence suggests that if high-quality health systems could effectively deliver a core subset of 19 maternal and child interventions, almost one quarter of the maternal deaths, neonatal deaths, and stillbirths would be prevented.[1] Access to ten evidence-based nutrition interventions alone could reduce the mortality rate in children under 5 by 15%.[2] Despite this, coverage data for high-impact interventions along the continuum of care remain limited due to challenges in data collection and measurement.[3] Improving the impact of nutrition interventions requires adequate measurement of both reach and quality of interventions. However, current global measurement mainly focuses on contact coverage indicators,[4] but does not adequately capture the quality of services delivered, thus providing only weak links with actual health benefits received by the population in need.[5]

Effective coverage has been variously defined as 'the fraction of maximum possible health gain an individual with a health care need can expect to receive from the health system'[6] and as a situation where 'people who need health services obtain them in a timely manner and at a level of quality necessary to obtain the desired effect and potential health gains.[7] Effective coverage goes beyond contact coverage measures by using not just the receipt of services by clients but also incorporating quality of care received. Multiple quality domains can be considered, including inputs (service and equipment availability, training etc.), service delivery processes (e.g. compliance with protocols and standards of care), and outcomes (health benefits, client satisfaction etc.).[8] However, different studies used various definitions and measurements for effective coverage, thus limiting the comparability among studies. To address this challenge, the recently proposed seven-step coverage framework begins with the

For peer review only - http://bmjopen.bmj.com/site/about/guidelines.xhtml

target client population, and following them through a hypothetical cascade of the losses of health benefits at different stages, including service contact, input-adjusted coverage, intervention coverage, quality-adjusted coverage (receives health service according to standard), user adherence -adjusted coverage and outcome-adjusted coverage (health gain achieved).[5 9]

Effective coverage have been found considerably lower than contact coverage for antenatal care (ANC),[10 11] family planning, and sick-child care in several low and middle income countries (LMICs).[11] Findings from LMICs also suggested low levels of availability, readiness, and coverage of obstetric services[12] and showed substantial reductions (20-39 percentage points, pp) from contact coverage to effective coverage in facility birth.[13] Overall, a review of 36 studies showed large gaps between contact coverage and quality-adjusted coverage levels (10 to 38 pp) across the continuum of care for reproductive, maternal, newborn, and child health.[5]

Although recent studies have paid more attention to capture the quality of care and quantify the gaps between contact coverage and effective coverage across continuum of care, studies on nutrition-related interventions are scarce. Previous studies mainly came from small-scale studies that focused on fortified foods where contact coverage was measured through exposure to a particular fortified food and quality-adjusted measures were based on regular consumption of the fortified food.[5] Recently, one study in Malawi shows that even though the utilization of ANC and facility delivery was high, women often did not received nutrition-related interventions (such as iron interventions, nutrition counseling and breastfeeding counseling).[14]

Bangladesh has observed significant improvement in contact coverage in maternal and child health in the last decade,[15] yet substantial gaps remained in coverage of nutrition services,

For peer review only - http://bmjopen.bmj.com/site/about/guidelines.xhtml

with less than 30% of women receiving all four key ANC nutrition services, and only 25% of sick children having their weight checked against a growth-chart.[16] There were also large gaps in facility readiness to provide nutrition services. Specifically, only around half of providers reported having received any training on nutrition, and only 13% of facilities possessed more than 5 of the 13 items deemed essential for service delivery for children.[16] Most previous studies in Bangladesh reported contact coverage[17-19] and quality of services provided[16 20] in a separate manner. This paper aimed to adjust contact-based health coverage estimates, taking into consideration the inputs required to deliver quality nutrition services, across the continuum of care in Bangladesh, specifically ANC and delivery for women and growth monitoring and curative care for young children.

For peer review only - http://bmjopen.bmj.com/site/about/guidelines.xhtml

DATA AND METHODS

Data sources

We used two sets of publicly-available nationally representative data: the 2014 Bangladesh Demographic and Health Survey (DHS)[21] and the 2014 Service Provision Assessment (SPA).[22] The DHS surveys are population-based household surveys that provide representative data on health and nutrition indicators at national and regional levels.[21] The survey is based on a two-stage stratified sample of households. In the first stage, 600 enumeration areas were selected with probability proportional to the enumeration area size. In the second stage of sampling, a systematic sample of 30 households on average was selected by equal probability sampling from a household list in each selected cluster. All women in selected households with a birth in the three years before the survey were interviewed about antenatal care and birth care for the most recent live birth. Child curative care questions were asked for all living child under 5 years in the household.

The SPA is a formal health facility-based survey designed to provide information on health service availability and readiness, with an emphasis on the delivery of reproductive, maternal, newborn and child health services.[22] The Bangladesh SPA included a facility inventory and a health care provider interview. Facilities are assessed for the availability of health services, basic amenities, equipment, laboratory services, essential medicines, and human resources. During the facility inventory, the survey team attempts to observe elements of routinely offered care (such as weighing children, plotting child's weight on graph, etc.). Health facilities are sampled from a complete listing of 19,184 registered health facilities in the country, with the aims to provide the survey results separately for the seven administrative

For peer review only - http://bmjopen.bmj.com/site/about/guidelines.xhtml

divisions and different types of public facilities, NGO clinics/hospitals, and private hospitals. Small private clinics with less than 20 beds are excluded. Further details on the survey's sampling strategy are presented in the survey report.[22]

Statistical analysis

Input-adjusted coverage for nutrition intervention is calculated among individuals in need of care as the product of the coverage of services and the readiness of health facilities to provide quality care. The selected indicators for estimating input-adjusted coverage for nutrition interventions across continuum of care are presented in Table 1.

Coverage of services

We estimated coverage of services based on DHS data, accounting for the DHS sampling weights. Coverage of ANC care was defined as the percentage of women 15–49 years old with at least one live birth in the three years preceding the survey who reported at least 4 ANC visits (one of those visits from a trained provider) for their most recent birth. Coverage of institutional delivery was defined as the percentage of women with a live birth in the 3 years preceding the survey gave birth in a health facility. There is no consensus on coverage measurement for child growth monitoring. However, growth monitoring activities, such as height, weight and mid-upper arm circumference (MUAC) measurement, are intended to be a core part of the Integrated Management of Childhood Illness (IMCI) protocol that is followed during sick-child contacts[16] which has been shown to have an impact on childhood stunting.[23] Based on this, and given the lack of survey questions that explicitly ask respondents about growth monitoring services, coverage of growth monitoring was defined as the percentage of sick children who sought treatment at a formal health facility among all children under 5 years

For peer review only - http://bmjopen.bmj.com/site/about/guidelines.xhtml

of age. Coverage of sick-child care was defined as the percentage of children with any illness who sought treatment at a formal health facility.

Facility readiness to provide nutrition interventions

We analyzed facility readiness to deliver nutrition interventions based on six attributes as guided by the World Health Organization (WHO)'s Service Availability and Readiness Assessment Manual[24] including availability of: 1) trained personnel, 2) guidelines, 3) equipment, 4) diagnostic capacity, and 5) medicines as well as 6) observation of activities occurring at the time of the visit. During ANC, these attributes are essential for delivering several nutrition interventions such as iron folic acid (IFA) supplementation, weight monitoring for pregnant women, anemia management, blood pressure management, fetal growth monitoring and health and nutrition education (Table 1). During birth, the main nutrition intervention is support for early breastfeeding practices. During postnatal care, protocols are for children to be weighed and to receive nutrition education, vitamin A supplementation, deworming anemia management, and ORS and zinc for diarrhea during sick-child care.

We calculated the composite readiness score using an equal-weight approach. The sum of all attributes was standardized to have a maximum of 100 to show the percentage of the readiness of the facility. The calculated readiness score for a specific facility category is an average score of all facilities in the same category. We accounted for the SPA sampling weight when summarizing readiness scores.

For peer review only - http://bmjopen.bmj.com/site/about/guidelines.xhtml

Table 1: Definition of indicators for estimating input-adjusted coverage for nutrition interventions across continuum of care

Services	Measure of need (denominator)	Measure of use (numerator)	Quality estimator
Nutrition interventions during antenatal care	Women 15–49 years old with at least one child under 3 years	Women 15–49 years old with at least one child under 3 years, whom for their most recent birth, reported at least 4 ANC visits (ANC4+)	Facility readiness score based on:<br>• Training staff<br>• ANC guideline<br>• Basic equipment<br>• Diagnostic capacity<br>• Essential medicines<br>• Activity reported/ observed in facilities<br>○ Iron and/or folic acid supplementation<br>○ Intermittent preventive treatment for malaria<br>○ Weighing of clients<br>○ Taking blood pressure<br>○ Conducting group health education sessions<br>○ Urine test for protein<br>○ Blood test for anemia<br>○ Measuring client's height<br>○ Ultrasonography
Nutrition interventions during birth care	Women 15–49 years old with at least one child under 5 years	Women 15–49 years old with at least one child under 5 years, whom for their most recent birth, reported delivery in a health facility	Facility level score based on:<br>• Staff training<br>• Guidelines on basic and comprehensive birth care<br>• Basic equipment<br>• Activity reported in facilities<br>○ Kangaroo Mother Care<br>○ Skin to skin contacts<br>○ Initiation of breastfeeding within the first hour<br>○ Do not give the newborn pre-lacteal liquids<br>○ Weigh the newborn immediately<br>○ Administer Vitamin K to newborn<br>○ Provide maternal Vitamin A
Child growth monitoring	All children alive between 0 and 59 months	All children who had diarrhea or ARI, for whom advice on treatment was sought from a medical provider	Facility level score based on staff with any training on growth monitoring, guidelines for growth monitoring, availability of equipment and growth chart for measuring child weight and height, observe health staff weighing the child and plotting child's weight on graph
Nutrition interventions during child sick care	All children alive between 0 and 59 months who had diarrhea or ARI in the last two weeks	All children who had diarrhea or ARI, for whom advice on treatment was sought from a medical provider	Facility-level score based on<br>• Training staff<br>• ANC guideline<br>• Basic equipment<br>• Diagnostic capacity<br>• Essential medicines<br>• Activity reported/ observed in facilities<br>○ Diagnose and/or treat child malnutrition<br>○ Provide vitamin A supplementation to children<br>○ Provide iron supplementation to children<br>○ Provide zinc supplementation to children<br>○ Provide deworming to children<br>○ Provide group health education

For peer review only

For peer review only - http://bmjopen.bmj.com/site/about/guidelines.xhtml

Estimating input-adjusted coverage

We linked health facility and household survey data at the aggregate level, using an ecological linking method which has been shown a feasible and valid approach for estimating quality-adjusted effective coverage.[25] We disaggregated the coverage by type of facility where services were sought. Facility types were harmonized between the DHS and SPA at 5 levels: District and upazila public facilities, union level public facilities, public community clinic, NGO clinic/hospital and private clinic/hospital (Supplemental Table 1).

Input-adjusted coverage was estimated at both the national and regional level (7 divisions), accounting for 5 types of facilities where the care was sought. At the regional level, the input-adjusted coverage is the summation of input-adjusted coverage of each type of facility that is constructed as the product of the coverage and readiness estimates:

$$IAC_r = \sum_j C_{rj} * Q_{rj}$$

where IAC_r represents input-adjusted coverage in region r,

C_{rj} is the proportion of women/children who sought care in facility type j in region r,

And Q_{rj} is the average readiness score of facility type j in region r.

The national input-adjusted coverage is the summation of regional input-adjusted coverage weighted by the proportion of users in each region:

$$IAC_T = \sum_r IAC_r * w_r$$

where w_r is the proportion of users in region r.

The variance estimation of input-adjusted coverage was assessed with an approximation procedure referred to as the "delta" method which has been used and described in detail in previous research.[26] Finally, we calculated the input-adjusted coverage by strata of place of

For peer review only - http://bmjopen.bmj.com/site/about/guidelines.xhtml

residence, educational level and socio-economic (SES) quintiles. This analysis assumes that there is no systematic difference in facility readiness within region by the education or socioeconomic stratifiers (e.g. that within a given sub-national region, the average readiness of public community clinics used by highly educated women was the same as average readiness of public community clinics used by less educated women). Data analysis was performed using Stata version 16.0.

Patient and public involvement

There was no patient and public involvement

RESULTS

The household survey included 17,863 women (out of 18,245 eligible women aged 15-49 years, a response rate of 98%), among them 4,488 women reporting a birth in the three years preceding the survey and 7,886 women with living children under 5 years. Health service coverage varied considerably between services, with low coverage for ANC4+ (28%), institutional delivery (38%), child growth monitoring (35%), but substantially higher for sick-child care (81%).

The health facility survey included 1,548 facilities of 1,596 sampled (97% response rate); among them 1,493 facilities (96%) offering services on antenatal care, 586 (38%) offering services on delivery care, and 1,463 (92%) offering curative care for sick children. The availability of inputs that are important for providing nutrition interventions across continuum of care is presented in Table 2 for the national level. Overall, facilities lacked trained personnel, guidelines, equipment, medicines or supplies needed to provide high quality of care (Table 2).

For peer review only - http://bmjopen.bmj.com/site/about/guidelines.xhtml

Of facilities offering each service, approximately half of facilities had staff with any training on ANC, child growth monitoring, and IMCI, but a lower proportion of facilities (39%) had staff with any training on integrated management of pregnancy and childbirth (IMPAC). Similarly, around half of facilities had national or other guidelines for ANC, growth monitoring or IMCI, but only 27% had guidelines for Basic Emergency Obstetric and Neonatal Care (BEmOC) or Comprehensive Emergency Obstetric and Neonatal Care (CEmOC). While a majority of facilities (>85%) had adult weighing scales, blood pressure monitors and stethoscopes, only 36% had tape measures for fundal height and 15% had fetal stethoscopes. Child scales, length or height board and growth chart were only available in 60-70% of facilities that offered sick-child care. Regarding diagnostic capacity, 11% of facilities were able to test for hemoglobin and 18% for urine protein. Iron-folic acid supplements were available in 91% of facilities. Zinc or vitamin A supplementation was reported in three quarters of facilities and deworming for children was reported in almost all facilities. The availability of these items varied by division (Supplemental Table 2) and by types of facilities (Supplemental Table 3).

When observing activities related to nutrition in ANC, around half of facilities took weight or measured blood pressure, a third conducted group health education sessions, and <10% conducted blood tests for anemia or urine tests for protein, and only 2% used ultrasonography to check on fetal growth. While health facilities performed well in providing supports for early breastfeeding during delivery care per facility self-report (70-98%), provision of services related to child growth assessment and nutritional management of illness was limited: only 41% of facilities reported or were observed measuring child weight, 27% plotting the child weight on a growth chart.

For peer review only - http://bmjopen.bmj.com/site/about/guidelines.xhtml

250 **Table 2: Percentage of health facilities with structural input items and their readiness score**

		% weighted	95% CI
**Antenatal care (n=1493 facilities)**			
**Staff**	Staff with any training on ANC	48.75	43.24-54.26
**Guideline**	National or other guidelines for ANC	49.64	41.88-57.40
	Visual aids for client education	66.93	60.71-73.15
**Equipment**	Adult weighting scale	85.44	81.83-89.06
	Tape measure for fundal height	36.17	29.37-42.97
	Blood pressure apparatus	91.22	88.21-94.24
	Stethoscope	91.93	89.06-94.79
	Fetal stethoscope	15.25	10.77-19.74
**Diagnostics capacity**	Hemoglobin	10.57	7.15-13.99
	Urine protein	17.87	13.62-22.12
**Medicine**	Iron or folic acid tablets	91.41	88.18-94.64
**Activity reported/ observed[1] in facilities**	Iron supplementation	82.19	76.42-87.97
	Acid folic supplementation	77.05	71.16-82.94
	Intermittent preventive treatment for malaria (IPT)	5.06	3.18-6.93
	Weighing of clients	49.89	41.20-58.59
	Taking blood pressure	50.19	41.05-59.34
	Urine test for protein	8.56	4.61-12.50
	Blood test for anemia	6.58	3.05-10.10
	Ultrasonography	1.97	0.98-2.97
	Conducting group health education sessions	34.02	25.81-42.23
Readiness score for ANC		***45.15***	***42.54-47.76***
**Birth care (n=586 facilities)**			
**Staff**	Staff with any training on IMPAC	38.74	30.01-47.47
**Guideline**	Guidelines for BEmOC or CEmOC	27.18	17.28-37.09
**Equipment**	Infant scale	60.64	52.62-68.65
	Manual or digital BP apparatus	93.35	89.08-97.62
**Activity reported in facilities**	Kangaroo practice for low birth weight babies	44.11	32.79-55.42
	Skin to skin contacts	70.11	60.58-79.65
	Initiation of breastfeeding within the first hour	97.19	94.54-99.83
	Do not give the newborn prelacteal liquids	77.49	69.09-85.90
	Weigh the newborn immediately	70.08	61.53-78.63
	Administer Vitamin K to newborn	48.54	38.58-58.50
	Provide maternal Vitamin A	60.45	50.73-70.18
Readiness score for birth care		***62.54***	***59.00-66.07***
**Child growth monitoring (n=1463 facilities)**			
**Staff**	Staff with any training on growth monitoring	48.76	42.46-55.07
**Guideline**	Guidelines for growth monitoring	46.65	39.28-54.01
**Equipment**	Child scale (child or infant)	69.00	63.08-74.92
	Length or height board	59.16	51.64-66.67
	Tape for measuring head	30.67	24.48-36.86
	Growth chart	66.70	60.66-72.73
**Activity reported/ observed[1] in facilities**	Weighing the child	40.92	31.83-50.00
	Plotting child's weight on graph	27.48	18.72-36.23
Readiness score for child growth monitoring		***47.61***	***43.33-51.90***
**Sick child care (n=1463 facilities)**			
**Staff**	Staff with any training on IMCI	53.85	47.36-60.33
**Guideline**	Guidelines for IMCI	50.84	44.29-57.39

For peer review only - http://bmjopen.bmj.com/site/about/guidelines.xhtml

		% weighted	95% CI
	IMCI chart booklet	53.35	47.13-59.56
	IMCI mother's cards (IMCI card)	39.48	33.50-45.45
	Other visual aids for teaching caretakers	56.23	50.36-62.09
**Equipment**	Child scale (child or infant)	60.80	54.16-67.44
**Diagnostics capacity**	Hemoglobin	10.34	6.85-13.84
**Medicine**	ORS	63.59	58.14-69.04
	Albendazole	87.59	83.68,91.50
	Mebendazole	12.23	8.04,16.42
	Iron tablet	54.35	46.15,62.56
	Vitamin A	66.52	60.60,72.44
	Zinc tablet	65.75	59.92,71.57
	Zinc sulphate syrup	18.02	12.97,23.08
**Activity reported/ observed[1] in facilities**	Diagnose and/or treat child malnutrition	93.33	90.57-96.09
	Provide vitamin A supplementation to children	74.44	70.55-78.34
	Provide iron supplementation to children	64.03	57.31-70.75
	Provide zinc supplementation to children	75.05	70.19-79.90
	Provide deworming to children	97.44	96.43-98.46
	Providing group health education	35.45	26.30-44.61
	Weighing the child	40.92	31.83-50.00
	Plotting child's weight on graph	27.48	18.72-36.23
Readiness score for child sick care		***51.23***	***48.23-54.23***

[1]Survey team assessing whether they see these practices in general in the facility at the time of the visit.
ANC: antenatal care, BEmOC: Basic Emergency Obstetric and Neonatal Care, CEmOC: Comprehensive Emergency Obstetric and Neonatal Care, IMCI: integrated management of childhood illness, IMPAC: integrated management of pregnancy and childbirth, ORS: Oral rehydration salts.

Readiness scores were calculated at the national (Table 2) and regional levels and by facility type (Figure 1). Overall, facilities demonstrated 45% of the expected resources to provide nutrition services during ANC, 63% for delivery care, and approximately half the required resources for postnatal care. Readiness scores varied by regions and depending on the service, where Rangpur performed the best overall, Khulna showed high readiness for ANC but average for delivery care, child growth monitoring and sick-child care, and Sylhet is the lowest on delivery care but average on others (Figure 1). District and upazila public facilities had the highest readiness scores across continuum of care, whereas lower-level facilities (union level and public community) were much less prepared. For example, district public hospitals had the

For peer review only - http://bmjopen.bmj.com/site/about/guidelines.xhtml

highest readiness score, with 64% of the expected capacity to provide nutrition interventions during ANC care services, but public community clinics had a readiness score of only 43%.

<Insert Figure 1 here>

Figure 2 plots health service coverage against facilities' readiness score in each region of the country. For ANC4+, the regional averages of coverage and readiness fell mostly in quadrant III for all regions, indicating both low coverage (ranged between 18-36%) and low readiness (between 42-50%) (Table 3), thus the priority is improving coverage as well as quality of nutrition services for ANC care. In contrast, the priority for delivery care clearly lies in improving coverage: most regions had low access (less than 50% except Khulna) but had higher readiness (56-74%). For child growth monitoring, all regions showed low coverage (30-39%); Dhaka had the lowest readiness (38%) while other regions had a higher but wide range of readiness (46-62%). For sick-child care, regions had similar levels of high coverage (>75%) but showed lower and highly variable readiness.

<Insert Figure 2 here>

Taking into account the readiness of facilities to provide the nutrition service and the use of service, we estimated the input-adjusted coverage at the national and regional level (Table 3 and Figure 3). At the national level, input-adjusted coverage was 18% for ANC4+, 25% for delivery care, 21% for child growth monitoring and 51% for sick-child care. The gaps between contact and input-adjusted coverage ranged between 10-40 percentage points (pp).

<Insert Figure 3 here>

For peer review only - http://bmjopen.bmj.com/site/about/guidelines.xhtml

287 **Table 3: Estimated regional and national input-adjusted coverage of nutrition interventions across**
288 **continuum of care, Bangladesh 2014**

	Coverage	Readiness score	Input-adjusted coverage		
			Estimate	Lower bound	Upper bound
**Antenatal care 4+**					
Barisal	22.79	42.75	14.29	10.80	18.66
Chittagong	24.35	42.6	15.40	12.62	18.67
Dhaka	31.19	44.14	19.78	16.40	23.66
Khulna	36.21	44.34	22.77	18.57	27.60
Rajshahi	24.36	45.3	15.56	12.18	19.68
Rangpur	34.44	50.24	22.72	18.04	28.18
Sylhet	18.44	50.4	11.94	8.63	16.31
**Overall**	28.06	45.15	17.84	16.12	19.71
**Birth care**					
Barisal	30.38	55.86	19.26	14.47	25.16
Chittagong	35.49	61.61	24.03	19.91	28.69
Dhaka	40.91	61.56	26.84	22.40	31.81
Khulna	54.96	63.54	36.89	30.33	43.99
Rajshahi	39.23	62.72	26.35	22.27	30.89
Rangpur	34.52	73.91	23.93	20.00	28.35
Sylhet	22.87	53.47	14.86	11.68	18.74
**Overall**	37.73	62.54	25.12	23.04	27.31
**Child growth monitoring**					
Barisal	32.77	51.76	20.79	16.96	25.22
Chittagong	36.78	46.23	22.47	17.74	28.03
Dhaka	33.87	37.96	21.40	17.77	25.55
Khulna	29.79	46.19	18.43	15.52	21.74
Rajshahi	35.34	49.84	11.37	9.90	13.03
Rangpur	36.99	61.85	25.59	19.79	32.41
Sylhet	38.51	48.53	23.78	19.51	28.64
**Overall**	35.05	47.61	21.00	19.11	23.02
**Sick child care**					
Barisal	76.83	52.39	48.26	39.60	57.02
Chittagong	82.92	46.4	51.35	43.85	58.79
Dhaka	80.95	46.7	49.86	42.10	57.62
Khulna	77.28	46.99	46.04	37.43	54.90
Rajshahi	78.53	53.98	48.47	33.61	63.60
Rangpur	82.69	66.11	57.18	45.61	68.01
Sylhet	84.38	53.34	53.24	42.73	63.47
**Overall**	81.20	51.23	50.80	46.83	54.76

289 Lower bound and upper bound represent the lower and upper bounds of the 95% confidence interval of the
290 effective coverage

291

292

For peer review only - http://bmjopen.bmj.com/site/about/guidelines.xhtml

When examining the inequality gaps, we found that input-adjusted coverage varied by as much as 8-24 pp between regions within the country (Figure 3). Inequality in input-adjusted coverage was large during pregnancy and delivery (13-18 pp between urban and rural areas, 14-17 pp between low and high education, and 23-37 pp between the rich and the poor), but narrower for child growth monitoring and sick-child care (<2 pp) (Figure 4).

<Insert Figure 4 here>

DISCUSSION

Improving both coverage and quality of healthcare services is essential to achieving universal health coverage and achieving the third Sustainable Development Goal of ensuring healthy lives and promoting well-being for all at all ages.[27] Our study is one of very few to develop and apply nutrition-related metrics of effective coverage across the continuum of care by incorporating service readiness of nutrition interventions into coverage estimates of ANC and post-delivery care. Our findings show low levels of coverage across the continuum of care (28-38%), except for sick-child care (81%); we also find moderate levels of facility readiness to provide nutrition services (41-74%). After adjustment for readiness, the input-adjusted coverage was found to be 10-40 pp lower than the contact coverage, and there was high level of inequality between regions within the country, residential areas, education and SES status for ANC and birth care, but less so for sick-child care. Because our estimates focus on inputs to care and do not capture how often these inputs were provided in adherence with evidence-based protocols, they should be considered an upper limit of the quality of nutrition care actually received.

For peer review only - http://bmjopen.bmj.com/site/about/guidelines.xhtml

The large gap between contact and adjusted coverage in our study was aligned with a review of 36 studies finding a range of 10-38 pp reductions between crude coverage and quality-adjusted coverage across the continuum of care.[5] These findings indicate that women who sought care in a health facility did not necessarily receive the quality of care needed for them and their children. Our findings provide insight into health system challenges and indicate potential priorities for improvement.

For ANC, the priority is ensuring that all four contacts are provided by a trained provider, while improving coverage and quality simultaneously to reach high levels of effective coverage for nutrition services. We find a low coverage of 4+ ANC (28%), with evidence of inequities by wealth, education and place of residence. Evidence from a systematic review shows that both demand-side (such as maternal education, spousal education, marital status, women's employment, autonomy, cultural beliefs, media exposure, and household income) and supply-side factors (such as service availability and cost) influence ANC uptake.[28 29] Financial and transport constraints may be a greater barrier for women in remote and poorer areas.[30 31] Interventions such as mobile health technologies have been found to improve ANC attendance,[32] but improving coverage requires targeted investments to reach underserved communities. We also found low readiness of the health system to provide nutritional services during ANC (45%) which is consistent with a previous study in Bangladesh.[16] Our findings suggest that facility readiness can be improved by ensuring the availability of trained providers and necessary equipment, as well as strengthening diagnostic capacity.

For birth care, the priority clearly lies in improving coverage of facility-based deliveries while also improving readiness and provider compliance with labor management protocols to

For peer review only - http://bmjopen.bmj.com/site/about/guidelines.xhtml

increase support for early initiation of breastfeeding. Although the trend of institutional delivery has increased over time from 29% in 2011[33 34] to 38% at the time of the survey in 2014, nearly two third of all births still take place at home. Women's education interventions and community-level mobilization may increase the coverage of institutional delivery.[35 36] In terms of quality, facilities have moderate readiness (63%) to provide nutrition services, particularly activities related to early initiation of breastfeeding, but additional efforts are needed to improve the overall quality of service for facility-based delivery, including training staff, providing guidelines, emergency transport and necessary equipment.[37]

For postnatal care, the priority is improving both coverage and quality of nutrition services for child growth monitoring. While community-based management of acute malnutrition activities such as screening and growth monitoring has been prioritized under the Bangladesh National Nutrition Services,[38] there is little data on the coverage and uptake of these interventions. Growth monitoring activities are part of the IMCI protocol that is to be followed during sick-child contacts, but implementation is poor[16] and their reach across the population is limited by the reach of public health facilities and by inclusion in sick child care rather than in well-child care.[39] This suggests the need for widely available services to deliver preventive nutrition services, not only to identify malnourished children, but to detect growth faltering early through regular growth monitoring and counseling for all children. Coverage can be increased by generating community awareness about the need for these services and generating demand. To ensure quality, trained staff should be available and have the necessary equipment to take anthropometric measurements, correctly use growth charts, and counsel

For peer review only - http://bmjopen.bmj.com/site/about/guidelines.xhtml

caregivers. Other elements of overall sick-child care quality should be improved by ensuring that staffs are trained on IMCI, and that guidelines, equipment and medicine are available.

The inequality in effective coverage highlights targets for intervention according to administrative division and populations served. There is a clear variability in the effective coverage of indicators across administrative divisions, with some divisions (Khulna and Rangpur) performing better overall and others (Sylhet) performing poorly overall. For Sylhet specifically, poor performance may be because of its terrain, which makes many areas hard to reach. However, lessons should be shared from the successes of high-performing divisions such as Khulna and Rangpur, and focused strategic planning and action could help reduce regional disparities. Input-adjusted coverage also showed inequities by place of residence, education, and socioeconomic status. However, the size of these inequalities varied where the gaps were larger in the case of ANC and delivery, but smaller for child growth monitoring and sick-child care. Our findings of inequalities in coverage during pregnancy and delivery align with previous research finding that urban residence, higher education attainment (for the respondent and spouse), and higher wealth status significantly predict utilization of institutional delivery services[34] and 4+ ANC exposure[17]. Thus, there is a special need to specifically target underserved populations.

Our study has several strengths. Studies using data from household surveys often report contact coverage estimates that are limited by the respondent recall, but do not capture the content of these services nor their quality. Our study has reported both the services readiness (which was estimated from health facility data), and the contact coverage (which was drawn from household survey data) and combined these two to estimate input-adjusted

For peer review only - http://bmjopen.bmj.com/site/about/guidelines.xhtml

coverage, thus bringing our estimates closer into the spectrum of effective coverage. The household surveys and facility data were collected in the same year and were combined using valid ecological linking method[25] by using facility type and disaggregating by divisions and urban/rural location. Variance and precision of coverage indicators were derived from linked data using the recommended delta method[26]. By including nutrition interventions for multiple populations (urban vs. rural; varying levels of education and wealth quintile) in our analysis, we provide a potential tool to assess the health promotion capacity of the health system by highlighting underserved populations.

We acknowledge some of the limitations of this analysis. *First,* although we included various facility levels, the exclusion of small private clinics hospitals with less than 20 beds from the SPA could lead to an overestimation of quality for private sector users as larger facilities tend to have higher readiness than smaller ones. *Second,* contact coverage was estimated based on women's recall; however, women may not remember exactly where they received services, particularly if they went to more than one type of facility. We have addressed this by restricting the recall period of 3 years for ANC and birth delivery to minimize the recall bias, and using the highest-level facility type in case women providing multiple responses. *Third*, the assumption of no systematic difference in facility readiness within region by the education or socioeconomic stratifies may affect inequality estimates. In reality, differences in facilities in the same category, discrimination in provision care of underserved groups, and lack of individual capacity to extract value from the healthcare system may lead to systematic differences in quality experienced by women of different socioeconomic groups in the same tier of facilities,[40] leading to higher inequalities than are shown here.

For peer review only - http://bmjopen.bmj.com/site/about/guidelines.xhtml

CONCLUSION

Sustainable Development Goal 3 calls for achieving universal health coverage, which requires successfully covering underserved populations with healthcare services as well as ensuring that the quality of services is adequate. Nutrition interventions should be an integral part of health services for mothers and children; however, we find that nutrition input-adjusted coverage was suboptimal and varied sub-nationally and across the continuum of care in Bangladesh. Special efforts are needed to improve the reach as well as the quality of health and nutrition services to achieve the SDGs.

For peer review only

For peer review only - http://bmjopen.bmj.com/site/about/guidelines.xhtml

FIGURE LEGENDS

Figure 1: Readiness score of facilities to provide nutrition interventions, by division (A) and types of facility (B), Bangladesh 2014

A. By division

B. By facility type

Figure 2: Regional readiness score of facilities to provide nutrition interventions versus coverage, Bangladesh 2014

Figure 3: Input-adjusted coverage of nutrition interventions by region, Bangladesh 2014

Figure 4: Inequality in input-adjusted coverage of nutrition interventions, Bangladesh 2014

SUPPLEMENTARY FILES

Supplementary table 1: Harmonized facility categories and reported categories in SPA and DHS

Supplemental Table 2: Percentage of health facilities with structural input items, by division

Supplemental Table 3: Percentage of health facilities with structural input items, by facility type

Supplemental Figure 1: Place of ANC and child care by facility type, Bangladesh 2014

Supplemental Figure 2: Distribution of facilities with ANC and sick child care by facility type, Bangladesh 2014

For peer review only - http://bmjopen.bmj.com/site/about/guidelines.xhtml

For peer review only

AUTHOR CONTRIBUTIONS

PHN conceived the manuscript, conducted the statistical analysis, wrote significant sections of the manuscript and revised the manuscript.

LQK conducted the statistical analysis, prepared tables and figures for the manuscript.

PP conducted the literature review, wrote significant sections of the manuscript and revised the manuscript.

MB supported data interpretation, reviewed and edited the manuscript.

PM supported data interpretation, reviewed and edited the manuscript.

EP supported data interpretation, reviewed and edited the manuscript.

HHL conceived the manuscript, advised on statistical analysis, supported data interpretation, and revised the manuscript.

All authors read and approved the final submitted manuscript.

FUNDING: Bill & Melinda Gates Foundation through POSHAN, led by International Food Policy Research Institute.

CONFLICT OF INTEREST STATEMENT*:* Authors declare no conflicts of interest.

PATIENT AND PUBLIC INVOLVEMENT: Patients and/or the public were not involved in the design, or conduct, or reporting, or dissemination plans of this research.

DATA AVAILABILITY STATEMENT: No additional data are available.

For peer review only - http://bmjopen.bmj.com/site/about/guidelines.xhtml

For peer review only

451 **ETHICAL APPROVAL**

452 The survey and its procedures were properly reviewed and approved by the authority of the
453 National Institute of Population Research and Training (NIPORT) of the Ministry of Health and
454 Family Welfare.

For peer review only

For peer review only - http://bmjopen.bmj.com/site/about/guidelines.xhtml

REFERENCES

1. Chou VB, Walker N, Kanyangarara M. Estimating the global impact of poor quality of care on maternal and neonatal outcomes in 81 low- and middle-income countries: A modeling study. *PLoS Med* 2019;16(12):e1002990. doi: 10.1371/journal.pmed.1002990 [published Online First: 2019/12/19]
2. Bhutta ZA, Das JK, Rizvi A, et al. Evidence-based interventions for improvement of maternal and child nutrition: what can be done and at what cost? *Lancet* 2013;382(9890):452-77. doi: 10.1016/S0140-6736(13)60996-4 [published Online First: 2013/06/12]
3. Gillespie S, Menon P, Heidkamp R, et al. Measuring the coverage of nutrition interventions along the continuum of care: time to act at scale. *BMJ Glob Health* 2019;4(Suppl 4):e001290. doi: 10.1136/bmjgh-2018-001290 [published Online First: 2019/07/13]
4. Countdown to 2030 Collaboration. Countdown to 2030: tracking progress towards universal coverage for reproductive, maternal, newborn, and child health. *Lancet* 2018;391(10129):1538-48. doi: 10.1016/S0140-6736(18)30104-1 [published Online First: 2018/02/06]
5. Amouzou A, Leslie HH, Ram M, et al. Advances in the measurement of coverage for RMNCH and nutrition: from contact to effective coverage. *BMJ Glob Health* 2019;4(Suppl 4):e001297. doi: 10.1136/bmjgh-2018-001297 [published Online First: 2019/07/13]
6. Shengelia B, Tandon A, Adams OB, et al. Access, utilization, quality, and effective coverage: an integrated conceptual framework and measurement strategy. *Soc Sci Med* 2005;61(1):97-109. doi: 10.1016/j.socscimed.2004.11.055 [published Online First: 2005/04/26]
7. Boerma T, AbouZahr C, Evans D, et al. Monitoring intervention coverage in the context of universal health coverage. *PLoS Med* 2014;11(9):e1001728. doi: 10.1371/journal.pmed.1001728 [published Online First: 2014/09/23]
8. Donabedian A. Evaluating the quality of medical care. 1966. *Milbank Q* 2005;83(4):691-729. doi: 10.1111/j.1468-0009.2005.00397.x [published Online First: 2005/11/11]
9. Marsh AD, Muzigaba M, Diaz T, et al. Effective coverage measurement in maternal, newborn,child, and adolescent health and nutrition: progress, future prospects, and implications for quality health systems. *Lancet Glob Health* 2020;8:e730–36.
10. Kanyangarara M, Munos MK, Walker N. Quality of antenatal care service provision in health facilities across sub-Saharan Africa: Evidence from nationally representative health facility assessments. *J Glob Health* 2017;7(2):021101. doi: 10.7189/jogh.07.021101 [published Online First: 2017/11/23]
11. Leslie HH, Malata A, Ndiaye Y, et al. Effective coverage of primary care services in eight high-mortality countries. *BMJ Glob Health* 2017;2(3):e000424. doi: 10.1136/bmjgh-2017-000424 [published Online First: 2018/04/11]
12. Kanyangarara M, Chou VB, Creanga AA, et al. Linking household and health facility surveys to assess obstetric service availability, readiness and coverage: evidence from 17 low- and middle-income countries. *J Glob Health* 2018;8(1):010603. doi: 10.7189/jogh.08.010603 [published Online First: 2018/06/05]
13. Wang W, Mallick L, Allen C, et al. Effective coverage of facility delivery in Bangladesh, Haiti, Malawi, Nepal, Senegal, and Tanzania. *PLoS One* 2019;14(6):e0217853. doi: 10.1371/journal.pone.0217853 [published Online First: 2019/06/12]
14. Joseph NT, Piwoz E, Lee D, et al. Examining coverage, content, and impact of maternal nutrition interventions: the case for quality-adjusted coverage measurement. *Journal of Global Health* 2020;10: 010501
15. NIPORT. Bangladesh Demographic and Health Survey 2017-18: Key Indicators. Dhaka, Bangladesh, and Rockville, Maryland, USA: NIPORT and ICF, 2019.

For peer review only - http://bmjopen.bmj.com/site/about/guidelines.xhtml

16. Billah SM, Saha KK, Khan ANS, et al. Quality of nutrition services in primary health care facilities: Implications for integrating nutrition into the health system in Bangladesh. *PLoS One* 2017;12(5):e0178121. doi: 10.1371/journal.pone.0178121 [published Online First: 2017/05/26]
17. Islam MM, Masud MS. Health care seeking behaviour during pregnancy, delivery and the postnatal period in Bangladesh: Assessing the compliance with WHO recommendations. *Midwifery* 2018;63:8-16. doi: 10.1016/j.midw.2018.04.021 [published Online First: 2018/05/15]
18. Ali N, Sultana M, Sheikh N, et al. Predictors of Optimal Antenatal Care Service Utilization Among Adolescents and Adult Women in Bangladesh. *Health Serv Res Manag Epidemiol* 2018;5:2333392818781729. doi: 10.1177/2333392818781729 [published Online First: 2018/08/08]
19. Anwar I, Nababan HY, Mostari S, et al. Trends and inequities in use of maternal health care services in Bangladesh, 1991-2011. *PLoS One* 2015;10(3):e0120309. doi: 10.1371/journal.pone.0120309 [published Online First: 2015/03/24]
20. Biswas TK, Sujon H, Rahman MH, et al. Quality of maternal and newborn healthcare services in two public hospitals of Bangladesh: identifying gaps and provisions for improvement. *BMC Pregnancy Childbirth* 2019;19(1):488. doi: 10.1186/s12884-019-2656-1 [published Online First: 2019/12/12]
21. NIPORT. National Institute of Population Research and Training (NIPORT), Mitra and Associates, and ICF International. Bangladesh Demographic and Health Survey 2014. Dhaka, Bangladesh, and Rockville, Maryland, USA: NIPORT, Mitra and Associates, and ICF International, 2016.
22. NIPORT. National Institute of Population Research and Training (NIPORT), Associates for Community and Population Research (ACPR), and ICF International. 2016. Bangladesh Health Facility Survey 2014. Dhaka, Bangladesh: NIPORT, ACPR, and ICF International, 2016.
23. Arifeen SE, Hoque DM, Akter T, et al. Effect of the Integrated Management of Childhood Illness strategy on childhood mortality and nutrition in a rural area in Bangladesh: a cluster randomised trial. *Lancet* 2009;374(9687):393-403. doi: 10.1016/S0140-6736(09)60828-X [published Online First: 2009/08/04]
24. WHO. Service Availability and Readiness Assessment (SARA). An annual monitoring system for service delivery. Reference Manual. Geneva, Switzerland: World Health Organization 2013.
25. Munos MK, Maiga A, Do M, et al. Linking household survey and health facility data for effective coverage measures: a comparison of ecological and individual linking methods using the Multiple Indicator Cluster Survey in Cote d'Ivoire. *J Glob Health* 2018;8(2):020803. doi: 10.7189/jogh.08.020803 [published Online First: 2018/11/10]
26. Sauer SM, Pullum T, Wang W, et al. Variance estimation for effective coverage measures: A simulation study. *Journal of Glob Health (in press)* 2020
27. UNDP. Sustainable Development Goals Geneva, Switzerland: United Nations; 2015 [Available from: https://sustainabledevelopment.un.org/sdg3 accessed 12/03 2020.
28. Simkhada B, Teijlingen ER, Porter M, et al. Factors affecting the utilization of antenatal care in developing countries: systematic review of the literature. *J Adv Nurs* 2008;61(3):244-60. doi: 10.1111/j.1365-2648.2007.04532.x [published Online First: 2008/01/17]
29. Haider MR, Qureshi ZP, Khan MM. Effects of women's autonomy on maternal healthcare utilization in Bangladesh: Evidence from a national survey. *Sex Reprod Healthc* 2017;14:40-47. doi: 10.1016/j.srhc.2017.09.002 [published Online First: 2017/12/03]
30. Haque MA, Dash SK, Chowdhury MA. Maternal health care seeking behavior: the case of Haor (wetland) in Bangladesh. *BMC Public Health* 2016;16:592. doi: 10.1186/s12889-016-3296-2 [published Online First: 2016/07/20]

For peer review only - http://bmjopen.bmj.com/site/about/guidelines.xhtml

31. Islam MR, Odland JO. Determinants of antenatal and postnatal care visits among Indigenous people in Bangladesh: a study of the Mru community. *Rural Remote Health* 2011;11(2):1672. [published Online First: 2011/07/01]
32. Watterson JL, Walsh J, Madeka I. Using mHealth to Improve Usage of Antenatal Care, Postnatal Care, and Immunization: A Systematic Review of the Literature. *Biomed Res Int* 2015;2015:153402. doi: 10.1155/2015/153402 [published Online First: 2015/09/18]
33. NIPORT. National Institute of Population Research and Training (NIPORT), Mitra and Associates, and ICF International. Bangladesh Demographic and Health Survey 2011. Dhaka, Bangladesh, and Rockville, Maryland, USA: NIPORT, Mitra and Associates, and ICF International, 2013.
34. Yaya S, Bishwajit G, Ekholuenetale M. Factors associated with the utilization of institutional delivery services in Bangladesh. *PLoS One* 2017;12(2):e0171573. doi: 10.1371/journal.pone.0171573 [published Online First: 2017/02/14]
35. Sharma BB, Jones L, Loxton DJ, et al. Systematic review of community participation interventions to improve maternal health outcomes in rural South Asia. *BMC Pregnancy Childbirth* 2018;18(1):327. doi: 10.1186/s12884-018-1964-1 [published Online First: 2018/08/12]
36. Lassi ZS, Bhutta ZA. Community-based intervention packages for reducing maternal and neonatal morbidity and mortality and improving neonatal outcomes. *Cochrane Database Syst Rev* 2015(3):CD007754. doi: 10.1002/14651858.CD007754.pub3 [published Online First: 2015/03/25]
37. NIPORT. National Institute of Population Research and Training (NIPORT), Associates for Community and Population Research (ACPR), and ICF International. 2020. Bangladesh Health Facility Survey 2017. Dhaka, Bangladesh: NIPORT, ACPR, and ICF International, 2020.
38. MoH&FW. Operational Plan For 'National Nutrition Services' July 2011-June 2016. In: Welfare MoHaF, ed., 2011.
39. Saha KK, Billah SM, Menon P, et al. Bangladesh National Nutrition Services: assessment of implementation status: The World Bank, 2015.
40. Sharma J, Leslie HH, Kundu F, et al. Poor Quality for Poor Women? Inequities in the Quality of Antenatal and Delivery Care in Kenya. *PLoS One* 2017;12(1):e0171236. doi: 10.1371/journal.pone.0171236 [published Online First: 2017/02/01]

For peer review only

For peer review only - http://bmjopen.bmj.com/site/about/guidelines.xhtml

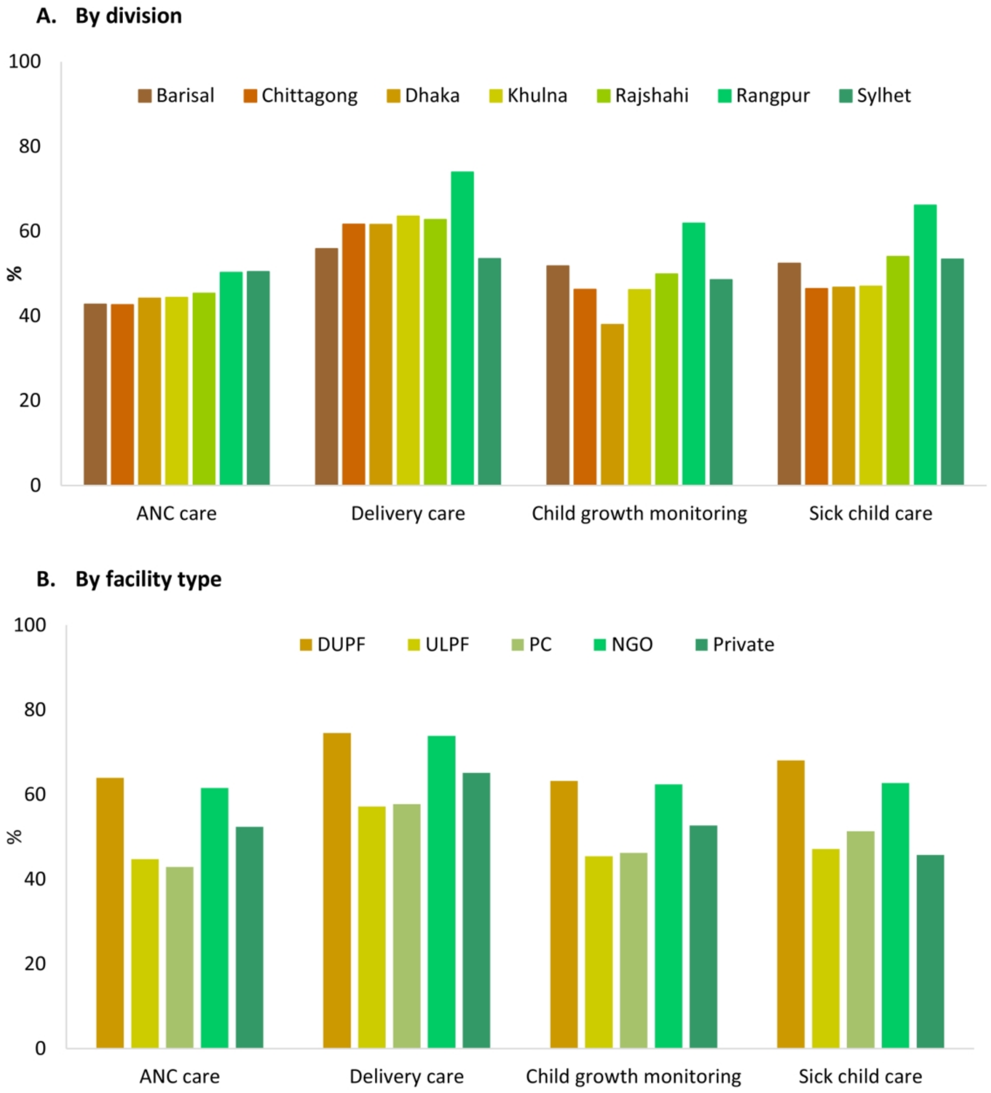

Figure 1

162x181mm (300 x 300 DPI)

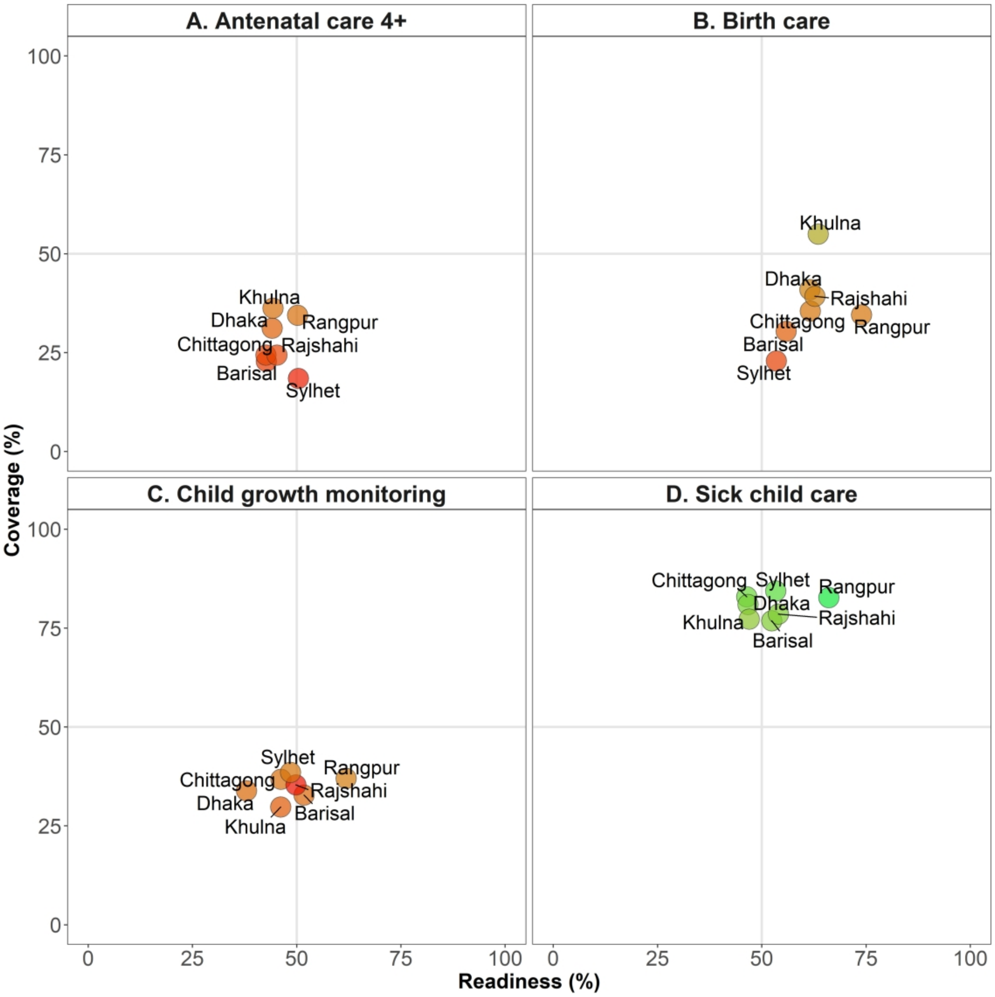

Figure 2

304x304mm (300 x 300 DPI)

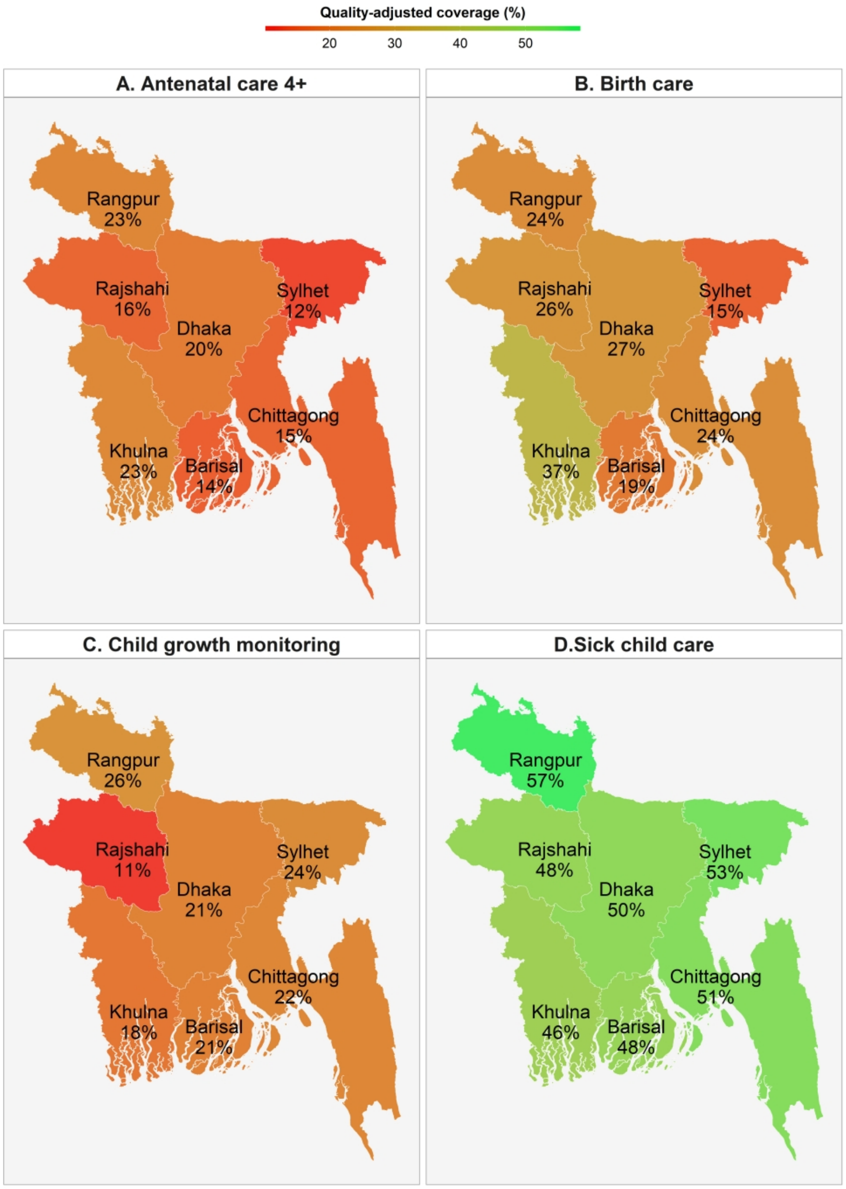

Figure 3

271x380mm (300 x 300 DPI)

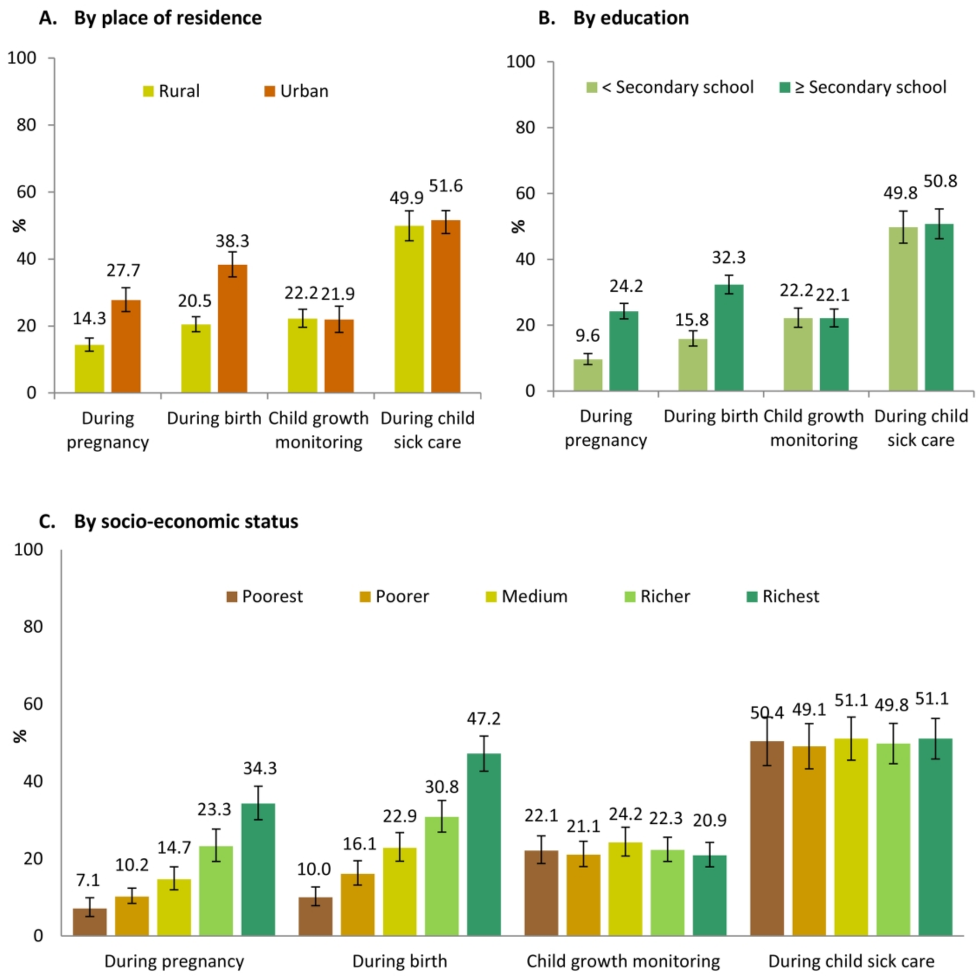

Figure 4

180x180mm (300 x 300 DPI)

Supplemental Table 1. Harmonized facility categories and reported categories in SPA and DHS

Harmonized facility category	SPA facility category	DHS facility category
District and upazila public facilities	District hospital (DH)<br>Upazila health complex (UHC)<br>Maternal and child welfare center (MCWC)	District hospital (DH)<br>Upazila health complex (UHC)<br>Maternal and child welfare center (MCWC)
Union level public facilities	Union health and family welfare center (UHFWC)/(UHFWC upgraded)<br>Union subcenter/rural dispensary	Upazila health & family welfare center<br>Satellite clinic/epi outreach
Public community clinic	Public community clinic	Public community clinic<br>Other public sectors
NGO clinic/hospital	NGO clinic/hospital	NGO static clinic<br>NGO satellite clinic<br>Other NGO clinic
Private clinic/hospital	Private clinic/hospital	Private clinic/hospital<br>Private medical<br>Traditional/Qualified doctor

For peer review only

Supplemental Table 2: Percentage of health facilities with structural input items, by division

		Barisal	Chittagong	Dhaka	Khulna	Rajshahi	Rangpur	Sylhet
**Antenatal care**								
**Staff**	Staff with any training on ANC	38.55	57.24	57.8	44.27	35.87	39.45	54.35
**Guideline**	National guidelines for ANC	56.26	38.49	39.93	58.47	46.86	70.65	60.09
	Visual aids for client education	69.80	63.78	57.09	72.27	67.74	72.19	91.51
**Equipment**	Adult weighting scale	88.74	89.73	72.93	91.45	87.21	93.23	90.38
	Tape measure for fundal height	41.18	27.51	33.42	40.77	32.44	50.37	36.35
	Blood pressure apparatus	87.03	86.88	90.67	94.51	92.03	97.85	88.96
	Stethoscope	89.75	86.32	92.10	96.73	93.69	96.06	87.98
	Fetal stethoscope	13.36	7.84	15.89	13.24	8.39	29.11	26.26
**Diagnostics capacity**	Hemoglobin	5.91	8.68	12.46	3.71	9.29	19.29	11.60
	Urine protein	8.28	15.84	20.06	11.35	18.58	26.71	18.58
**Medicine**	Iron or folic acid tablets	96.99	91.40	91.52	90.51	96.58	100.00	94.70
**Activity reported/ observed[1] in facilities**	Iron supplementation	67.04	78.58	87.45	73.48	90.69	78.73	94.23
	Acid folic supplementation	62.30	74.31	80.39	68.85	84.90	78.73	83.80
	Intermittent preventive treatment for malaria (IPT)	3.49	6.86	6.78	0.64	5.51	4.42	3.39
	Weighing of clients	56.52	50.54	43.05	51.27	51.65	52.14	58.28
	Taking blood pressure	57.73	47.82	47.39	53.24	51.94	47.16	56.5
	Urine test for protein	3.97	3.57	8.69	6.06	10.76	17.19	9.78
	Blood test for anemia	4.95	3.08	6.94	1.87	5.88	16.96	6.55
	Ultrasonography	0.45	0.88	5.04	1.29	0.74	0.54	0.70
	Conducting group health education sessions	33.90	31.37	30.7	32.61	34.47	42.42	40.18
**Birth care**								
**Staff**	Staff with any training on IMPACT	23.68	33.01	45.01	37.02	35.94	43.94	34.23
**Guideline**	Guidelines for BEmOC or CEmOC	32.66	26.00	25.83	21.19	10.84	56.25	20.46
**Equipment**	Infant scale	52.46	56.88	49.54	61.50	77.21	89.71	52.01
	Manual or digital BP apparatus	90.45	89.58	94.13	82.86	100.00	100.00	92.91
**Activity reported in facilities**	Kangaroo practice	35.64	41.26	48.20	48.44	37.55	47.06	39.57
	Skin to skin contacts	52.92	60.54	71.35	85.17	63.57	90.55	62.84
	Initiation of breastfeeding within the first hour	91.98	97.71	100.00	98.81	100.00	91.05	87.99
	Do not give the newborn prelacteal liquids	91.37	82.08	68.68	93.28	78.34	76.31	77.25
	Weigh the newborn immediately	65.43	64.54	67.81	67.99	84.63	82.27	55.60
	Administer Vitamin K to newborn	32.28	56.88	48.94	38.14	52.39	53.28	31.11
	Provide maternal Vitamin A	45.55	69.24	57.70	64.58	49.49	82.59	34.19
**Child growth monitoring**								
**Staff**	Staff with any training on growth monitoring	50.08	46.13	55.37	47.18	50.20	40.08	49.72
**Guideline**	Guidelines for growth monitoring	57.50	48.33	37.09	51.80	41.36	65.11	22.03
**Equipment**	Child scale (child or infant)	69.87	67.66	50.28	60.79	75.85	96.16	82.95
	Length or height board	70.34	49.76	50.48	56.34	66.41	69.23	72.63
	Tape for measuring head	32.28	24.25	23.24	30.86	27.20	43.00	50.57
	Growth chart	83.21	69.66	45.23	71.65	60.51	91.50	66.73
**Activity**	Weighing the child	39.71	40.74	34.46	40.04	43.46	47.86	42.85

For peer review only

		Barisal	Chittagong	Dhaka	Khulna	Rajshahi	Rangpur	Sylhet
**reported/observed in facilities**	Plotting child's weight on graph	24.45	27.65	21.23	23.10	38.82	34.38	18.48
Readiness score for child growth monitoring								
**Sick child care**								
**Staff**	Staff with any training on IMCI	67.19	48.62	57.54	42.75	52.18	55.86	58.89
**Guideline**	Guidelines for IMCI	66.62	42.83	38.33	51.17	45.03	77.60	58.55
	IMCI chart booklet	64.33	40.94	49.47	34.79	55.25	84.87	57.20
	IMCI mother's cards (IMCI card)	43.90	32.20	31.08	25.35	32.13	83.08	38.93
	Other visual aids for teaching caretakers	64.14	46.00	43.75	53.46	60.44	80.14	71.73
**Equipment**	Child scale (child or infant)	66.12	57.20	40.63	64.15	64.93	84.61	79.74
**Diagnostics capacity**	Hemoglobin	5.15	8.29	11.85	3.45	9.50	19.83	11.63
**Medicine**	ORS	71.97	63.52	61.01	51.95	74.91	69.94	52.35
**Activity reported/ observed[1] in facilities**	Diagnose and/or treat child malnutrition	97.01	94.28	84.28	97.59	99.69	94.59	98.87
	Provide vitamin A supplementation to children	72.65	69.00	72.35	78.79	75.49	86.22	64.50
	Provide iron supplementation to children	37.19	56.07	67.36	60.26	73.79	80.93	57.49
	Provide zinc supplementation to children	80.52	59.31	79.16	69.97	73.71	88.11	80.68
	Provide deworming to children	96.15	98.79	96.78	96.65	97.95	97.04	99.34
	Providing group health education	32.00	33.08	35.90	36.20	36.42	39.01	31.05
	Weighing the child	39.71	40.74	34.46	40.04	43.46	47.86	42.85
	Plotting child's weight on graph	24.45	27.65	21.23	23.10	38.82	34.38	18.48

[1]Survey team assessing whether they see these practices in general in the facility at the time of the visit.
ANC: antenatal care, BEmOC: Basic Emergency Obstetric and Neonatal Care, CEmOC: Comprehensive Emergency Obstetric and Neonatal Care, IMCI: integrated management of childhood illness, IMPAC: integrated management of pregnancy and childbirth, ORS: Oral rehydration salts.

For peer review only

Supplemental Table 3: Percentage of health facilities with structural input items, by facility type

		DUPF	ULPF	PC	NGO	Private
**Antenatal care**						
**Staff**	Staff with any training on ANC	83.64	50.04	46.31	60.36	27.48
**Guideline**	National or other guidelines for ANC	70.33	47.82	48.16	72.95	24.70
	Visual aids for client education	79.25	68.52	65.47	78.34	45.94
**Equipment**	Adult weighting scale	91.47	82.20	85.67	94.15	83.82
	Tape measure for fundal height	60.76	35.40	32.67	63.31	52.64
	Blood pressure apparatus	99.12	90.74	90.30	99.14	94.67
	Stethoscope	98.94	91.02	91.18	99.15	97.97
	Fetal stethoscope	46.35	20.38	8.67	46.55	43.29
**Diagnostics capacity**	Hemoglobin	61.97	7.90	3.53	58.87	72.14
	Urine protein	63.89	12.02	12.34	68.63	68.12
**Medicine**	Iron or folic acid tablets	96.41	94.51	94.57	89.92	66.29
**Activity reported/ observed[1] in facilities**	Iron supplementation	84.66	81.40	82.78	80.49	71.92
	Acid folic supplementation	79.12	76.67	77.17	78.80	69.00
	Intermittent preventive treatment for malaria (IPT)	21.74	5.34	2.97	10.97	30.99
	Weighing of clients	65.25	48.95	48.96	58.51	39.94
	Taking blood pressure	68.39	53.44	47.38	59.04	44.53
	Urine test for protein	32.42	3.89	5.80	34.68	35.79
	Blood test for anemia	33.24	4.08	3.06	29.73	35.79
	Ultrasonography	11.56	0.43	0.00	10.58	41.93
	Conducting group health education sessions	42.19	36.42	32.62	42.86	10.54
**Birth care**						
**Staff**	Staff with any training on IMPACT	55.96	31.75	40.71	52.84	22.05
**Guideline**	Guidelines for BEmOC or CEmOC	40.50	26.09	27.42	27.94	11.29
**Equipment**	Infant scale	69.52	60.47	47.87	76.72	64.97
	Manual or digital BP apparatus	96.91	88.33	93.18	99.34	100.00
**Activity reported in facilities**	Kangaroo practice	61.43	33.04	40.84	57.97	51.62
	Skin to skin contacts	87.27	70.94	54.47	73.82	75.7
	Initiation of breastfeeding within the first hour	97.64	94.27	100.00	100.00	97.34
	Do not give the newborn prelacteal liquids	71.24	88.78	72.36	73.56	65.14
	Weigh the newborn immediately	85.97	61.98	55.57	92.25	88.85
	Administer Vitamin K to newborn	75.49	33.57	26.25	78.65	84.73
	Provide maternal Vitamin A	77.54	39.63	75.85	79.06	54.13
**Child growth monitoring**						
**Staff**	Staff with any training on growth monitoring	67.88	42.91	50.18	47.73	40.35
**Guideline**	Guidelines for growth monitoring	52.55	43.30	46.22	64.57	29.31
**Equipment**	Child scale (child or infant)	82.28	74.50	64.19	86.20	90.63
	Tape for measuring head	47.65	26.41	28.95	57.15	32.90
	Length or height board	71.77	52.26	60.09	71.51	57.32
	Growth chart	85.51	68.72	63.01	83.71	85.13
**Activity reported / observed in facilities**	Weighing the child	66.02	39.98	38.21	59.13	38.95
	Plotting child's weight on graph	40.61	24.96	26.54	43.87	18.55
Readiness score for child growth monitoring						
**Sick child care**						
**Staff**	Staff with any training on IMCI	78.46	52.70	54.11	45.91	32.97
**Guideline**	Guidelines for IMCI	76.74	55.46	47.93	56.32	30.23
	IMCI chart booklet	78.06	59.78	49.35	64.58	35.14

		DUPF	ULPF	PC	NGO	Private
	IMCI mother's cards (IMCI card)	62.65	50.15	33.78	52.56	24.58
	Other visual aids for teaching caretakers	65.70	54.39	55.88	68.49	41.49
**Equipment**	Child scale (child or infant)	84.76	66.10	55.77	80.08	77.59
**Diagnostics capacity**	Hemoglobin	62.53	8.18	3.78	58.33	68.26
**Medicine**	ORS	64.45	34.50	73.57	78.80	46.65
**Activity reported/ observed[1] in facilities**	Diagnose and/or treat child malnutrition	98.00	93.50	93.19	90.08	97.29
	Provide vitamin A supplementation to children	79.18	43.62	85.92	75.11	58.28
	Provide iron supplementation to children	72.69	65.67	62.35	72.55	65.12
	Provide zinc supplementation to children	79.93	43.65	86.10	80.36	71.78
	Provide deworming to children	98.28	94.35	99.02	96.54	79.25
	Providing group health education	40.29	37.11	35.2	37.89	6.09
	Weighing the child	66.02	39.98	38.21	59.13	38.95
	Plotting child's weight on graph	40.61	24.96	26.54	43.87	18.55

[1]Survey team assessing whether they see these practices in general in the facility at the time of the visit.
DUPF: District and upazila public facilities, ULPF: Union level public facilities Public community clinic
ANC: antenatal care, BEmOC: Basic Emergency Obstetric and Neonatal Care, CEmOC: Comprehensive Emergency Obstetric and Neonatal Care, IMCI: integrated management of childhood illness, IMPAC: integrated management of pregnancy and childbirth, ORS: Oral rehydration salts.

For peer review only

Supplemental Figure 1: Place of ANC and child care by facility type, Bangladesh 2014

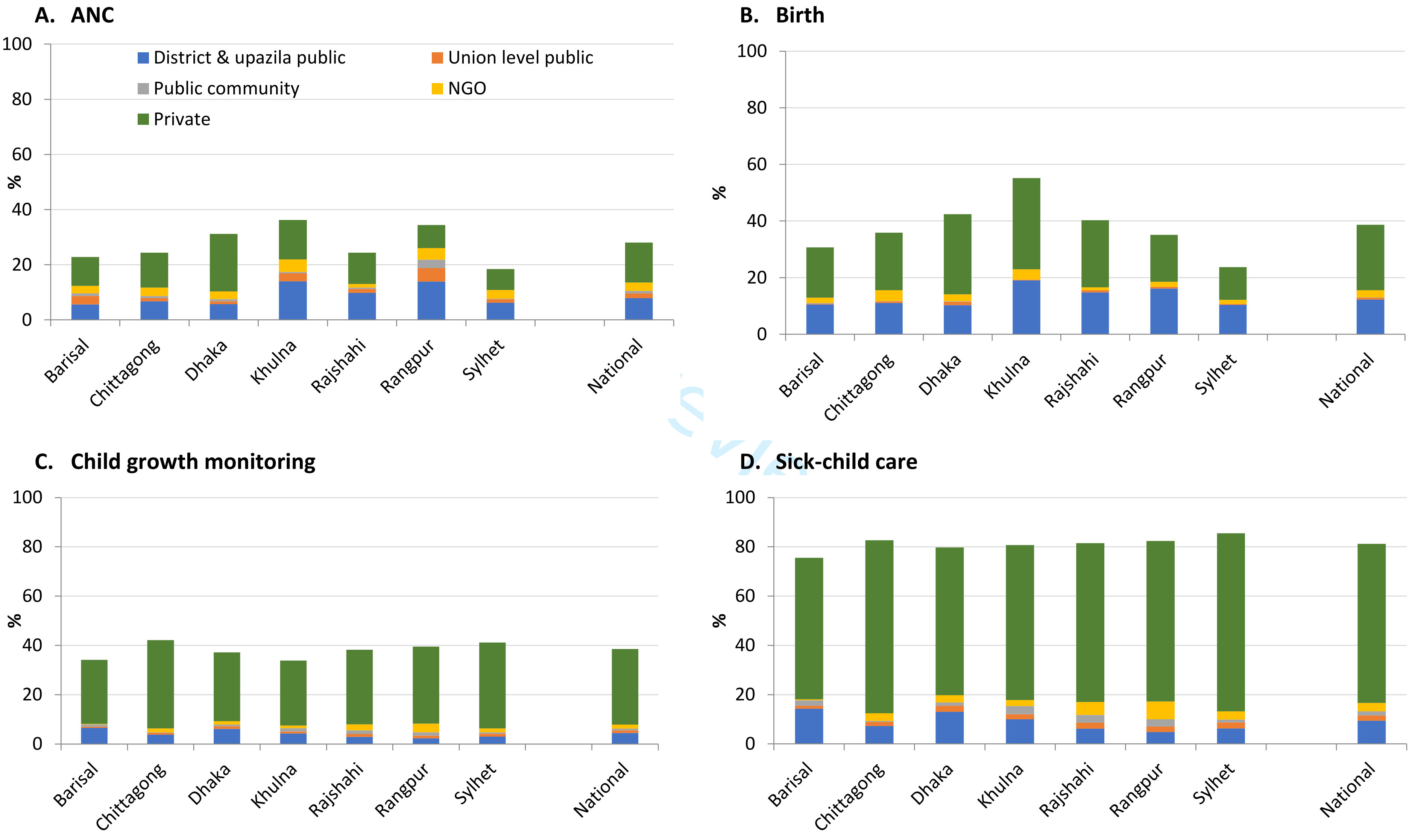

Source: DHS data

Supplemental Figure 2: Distribution of facilities with ANC and sick child care by facility type, Bangladesh 2014

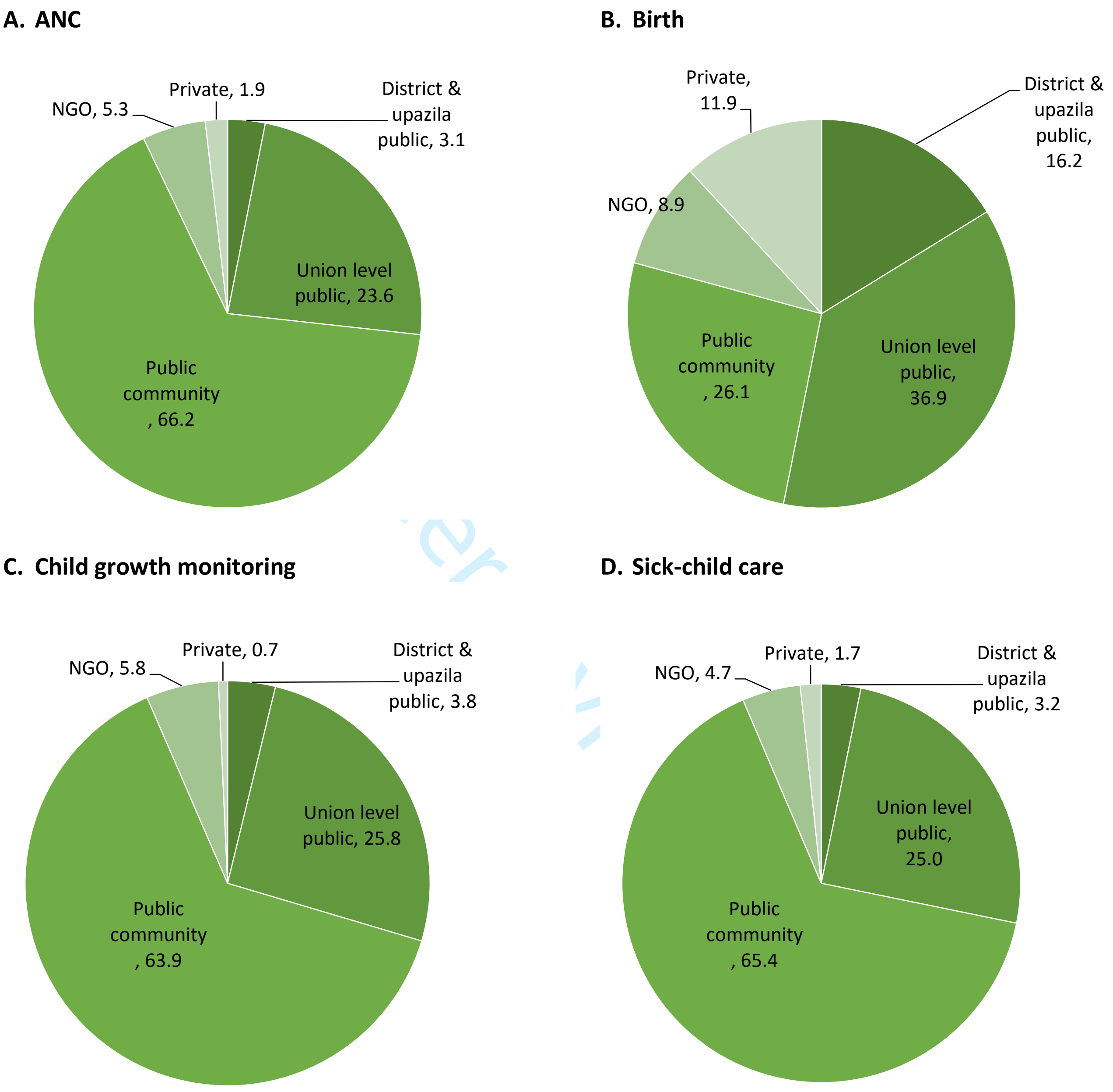

Source: SPA data

STROBE Statement—Checklist of items that should be included in reports of cross-sectional studies

	Item No	Recommendation	Page number from manuscript
**Title and abstract**	1	(*a*) Indicate the study's design with a commonly used term in the title or the abstract	3
		(*b*) Provide in the abstract an informative and balanced summary of what was done and what was found	3-4
**Introduction**			
Background/rationale	2	Explain the scientific background and rationale for the investigation being reported	6-8
Objectives	3	State specific objectives, including any prespecified hypotheses	8
**Methods**			
Study design	4	Present key elements of study design early in the paper	9-10
Setting	5	Describe the setting, locations, and relevant dates, including periods of recruitment, exposure, follow-up, and data collection	9-10
Participants	6	(*a*) Give the eligibility criteria, and the sources and methods of selection of participants	9-10
Variables	7	Clearly define all outcomes, exposures, predictors, potential confounders, and effect modifiers. Give diagnostic criteria, if applicable	10-12
Data sources/ measurement	8*	For each variable of interest, give sources of data and details of methods of assessment (measurement). Describe comparability of assessment methods if there is more than one group	10-12
Bias	9	Describe any efforts to address potential sources of bias	NA
Study size	10	Explain how the study size was arrived at	NA
Quantitative variables	11	Explain how quantitative variables were handled in the analyses. If applicable, describe which groupings were chosen and why	13-14
Statistical methods	12	(*a*) Describe all statistical methods, including those used to control for confounding	13-14
		(*b*) Describe any methods used to examine subgroups and interactions	13-14
		(*c*) Explain how missing data were addressed	NA
		(*d*) If applicable, describe analytical methods taking account of sampling strategy	13-14
		(*e*) Describe any sensitivity analyses	NA
**Results**			
Participants	13*	(a) Report numbers of individuals at each stage of study—eg numbers potentially eligible, examined for eligibility, confirmed eligible, included in the study, completing follow-up, and analysed	14
		(b) Give reasons for non-participation at each stage	NA
		(c) Consider use of a flow diagram	NA
Descriptive data	14*	(a) Give characteristics of study participants (eg demographic, clinical, social) and information on exposures and potential	14

For peer review only - http://bmjopen.bmj.com/site/about/guidelines.xhtml

		confounders	
		(b) Indicate number of participants with missing data for each variable of interest	NA
Outcome data	15*	Report numbers of outcome events or summary measures	15-20
Main results	16	(*a*) Give unadjusted estimates and, if applicable, confounder-adjusted estimates and their precision (eg, 95% confidence interval). Make clear which confounders were adjusted for and why they were included	15-20
		(*b*) Report category boundaries when continuous variables were categorized	NA
		(*c*) If relevant, consider translating estimates of relative risk into absolute risk for a meaningful time period	NA
Other analyses	17	Report other analyses done—eg analyses of subgroups and interactions, and sensitivity analyses	NA
**Discussion**			
Key results	18	Summarise key results with reference to study objectives	20
Limitations	19	Discuss limitations of the study, taking into account sources of potential bias or imprecision. Discuss both direction and magnitude of any potential bias	24
Interpretation	20	Give a cautious overall interpretation of results considering objectives, limitations, multiplicity of analyses, results from similar studies, and other relevant evidence	21-24
Generalisability	21	Discuss the generalisability (external validity) of the study results	25
**Other information**			
Funding	22	Give the source of funding and the role of the funders for the present study and, if applicable, for the original study on which the present article is based	27

*Give information separately for exposed and unexposed groups.

Note: An Explanation and Elaboration article discusses each checklist item and gives methodological background and published examples of transparent reporting. The STROBE checklist is best used in conjunction with this article (freely available on the Web sites of PLoS Medicine at http://www.plosmedicine.org/, Annals of Internal Medicine at http://www.annals.org/, and Epidemiology at http://www.epidem.com/). Information on the STROBE Initiative is available at www.strobe-statement.org.

For peer review only

BMJ Open

Effective coverage of nutrition interventions across the continuum of care in Bangladesh: Insights from nationwide cross-sectional household and health facility surveys

Journal:	*BMJ Open*
Manuscript ID	bmjopen-2020-040109.R1
Article Type:	Original research
Date Submitted by the Author:	24-Sep-2020
Complete List of Authors:	Nguyen, Phuong Hong; International Food Policy Research Institute<br>Khương , Quỳnh Long ; Hanoi University of Public Health<br>Pramanik, Priyanjana ; International Food Policy Research Institute<br>Billah, Sk; ICDDRB, Maternal and Child Health Division<br>Menon, Purnima; International Food Policy Research Institute<br>Piwoz, Ellen; Bill and Melinda Gates Foundation,<br>Leslie, Hannah; Harvard TH Chan School of Public Health, Global Health and Population
**Primary Subject Heading**:	Health services research
Secondary Subject Heading:	Global health, Health services research, Public health
Keywords:	EPIDEMIOLOGY, International health services < HEALTH SERVICES ADMINISTRATION & MANAGEMENT, Quality in health care < HEALTH SERVICES ADMINISTRATION & MANAGEMENT, Public health < INFECTIOUS DISEASES, NUTRITION & DIETETICS

SCHOLARONE™
Manuscripts

I, the Submitting Author has the right to grant and does grant on behalf of all authors of the Work (as defined in the below author licence), an exclusive licence and/or a non-exclusive licence for contributions from authors who are: i) UK Crown employees; ii) where BMJ has agreed a CC-BY licence shall apply, and/or iii) in accordance with the terms applicable for US Federal Government officers or employees acting as part of their official duties; on a worldwide, perpetual, irrevocable, royalty-free basis to BMJ Publishing Group Ltd ("BMJ") its licensees and where the relevant Journal is co-owned by BMJ to the co-owners of the Journal, to publish the Work in this journal and any other BMJ products and to exploit all rights, as set out in our licence.

The Submitting Author accepts and understands that any supply made under these terms is made by BMJ to the Submitting Author unless you are acting as an employee on behalf of your employer or a postgraduate student of an affiliated institution which is paying any applicable article publishing charge ("APC") for Open Access articles. Where the Submitting Author wishes to make the Work available on an Open Access basis (and intends to pay the relevant APC), the terms of reuse of such Open Access shall be governed by a Creative Commons licence – details of these licences and which Creative Commons licence will apply to this Work are set out in our licence referred to above.

Other than as permitted in any relevant BMJ Author's Self Archiving Policies, I confirm this Work has not been accepted for publication elsewhere, is not being considered for publication elsewhere and does not duplicate material already published. I confirm all authors consent to publication of this Work and authorise the granting of this licence.

For peer review only

Effective coverage of nutrition interventions across the continuum of care in Bangladesh: Insights from nationwide cross-sectional household and health facility surveys

Authors:

Phuong Hong Nguyen[1*], Long Quynh Khuong[2], Priyanjana Pramanik[1], Sk Masum Billah[3 4], Purnima Menon[1], Ellen Piwoz[5], and Hannah H Leslie[6]

Author affiliations:

[1]International Food Policy Research Institute, Washington, DC, USA; [2]Hanoi University of Public Health, Hanoi, Viet Nam; [3]Maternal and Child Health Division, ICDDR, B, Dhaka, Bangladesh; [4]The University of Sydney, Faculty of Medicine and Health, Sydney School of Public Health, New South Wales, Australia [5]Bill & Melinda Gates Foundation; [6]Harvard T.H. Chan School of Public Health, Boston, MA, USA

***Corresponding author:**

Phuong Hong Nguyen, Poverty, Health and Nutrition Division, International Food Policy Research Institute, 2005 I Street, NW, Washington DC, 20005. Phone: 202-826-4088. Email: P.H.Nguyen@cgiar.org

Word count: 4,642 (from introduction to conclusion, not including tables)

Abbreviations

ANC	: Antenatal care
BEmONC	: Basic Emergency Obstetric and Neonatal Care
CEmONC	: Comprehensive Emergency Obstetric and Neonatal Care
DHS	: Demographic and Health Surveys
DUPF	: District and upazila public facilities

For peer review only - http://bmjopen.bmj.com/site/about/guidelines.xhtml

23	IFA	: Iron folic acid
24	IMCI	: Integrated management of childhood illness
25	IMPAC	: Integrated management of pregnancy and childbirth
26	LMIC	: Low- and middle-income countries
27	MUAC	: Mid-upper arm circumference
28	NGO	: Non-governmental organization
29	ORS	: Oral rehydration salts
30	PC	: Public community clinic
31	Pp	: Percentage points
32	SES	: Socio-economic status
33	SPA	: Service Provision Assessment
34	ULPF	: Union level public facilities
35	WHO	: World Health Organization

For peer review only - http://bmjopen.bmj.com/site/about/guidelines.xhtml

ABSTRACT

Introduction: Improving the impact of nutrition interventions requires adequate measurement of both reach and quality of interventions, but limited evidence exists on advancing coverage measurement. We adjusted contact-based coverage estimates, taking into consideration the inputs required to deliver quality nutrition services, to calculate input-adjusted coverage of nutrition interventions across the continuum of care from pregnancy through early childhood in Bangladesh.

Methods: We used data from the 2014 Bangladesh Demographic and Health Surveys to assess use of maternal and child health services and the 2014 Service Provision Assessment to determine facility readiness to deliver nutrition interventions. Service readiness captured availability of nutrition-specific services and inputs (including human resources and training, equipment, diagnostics, and medicines). Contact coverage was combined with service readiness to create a measure of input-adjusted coverage at the national and regional levels, across place of residence, and by maternal education and household socio-economic quintiles.

Results: Contact coverage varied from 28% for attending at least 4 ANC visits to 38% for institutional delivery, 35% for child growth monitoring and 81% for sick-child care. Facilities demonstrated incomplete readiness for nutrition interventions, ranging from 45 to 54% across services. Nutrition input-adjusted coverage was suboptimal (18% for ANC, 24% for institutional delivery, 20% for child growth monitoring and 52% for sick-child care) and varied between regions within the country. Inequalities in input-adjusted coverage were large during ANC and institutional delivery (13-17 percentage points [pp] between urban and rural areas, 14-16pp

For peer review only - http://bmjopen.bmj.com/site/about/guidelines.xhtml

For peer review only

between low and high education, and 27-35pp between highest and lowest wealth quintiles), and less variable for sick-child care (< 2pp).

Conclusion: Nutrition input-adjusted coverage was suboptimal and varied sub-nationally and across the continuum of care in Bangladesh. Special efforts are needed to improve the reach as well as the quality of health and nutrition services to achieve the Sustainable Development Goals.

Keywords: Bangladesh, facility readiness, input-adjusted coverage, nutrition, effective coverage

For peer review only

Strengths and limitations of this study

- Our study used nationally representative population and health system data to estimate input-adjusted coverage for nutrition interventions across the continuum of maternal and early childhood care.
- Our study quantified inequities in input-adjusted coverage within the population and indicated potential priorities for improvement, including special investment in reaching underserved populations with key services, while also ensuring adequate quality of services.
- Although the SPA survey was quite comprehensive, it did not capture every aspect to measure facility readiness to provide nutrition interventions or to calculate each step of care cascades from need for services through to health benefits.
- The exclusion of small private clinics hospitals with less than 20 beds could lead to an overestimation of quality for private sector users, as larger facilities tend to have higher readiness than smaller ones.

For peer review only - http://bmjopen.bmj.com/site/about/guidelines.xhtml

INTRODUCTION

Global evidence suggests that if high-quality health systems could effectively deliver a core subset of 19 maternal and child interventions, almost one quarter of the maternal deaths, neonatal deaths, and stillbirths would be prevented.[1] Access to ten evidence-based nutrition interventions alone could reduce the mortality rate in children under 5 by 15%.[2] Despite this, coverage data for high-impact interventions along the continuum of care remain limited due to challenges in data collection and measurement.[3] Improving the impact of nutrition interventions requires adequate measurement of both reach and quality of interventions. Current global measurement mainly focuses on contact coverage indicators,[4] but does not adequately capture the quality of services delivered, thus providing only weak links with actual health benefits received by the population in need.[5]

Effective coverage has been variously defined as 'the fraction of maximum possible health gain an individual with a health care need can expect to receive from the health system'[6] and as a situation where 'people who need health services obtain them in a timely manner and at a level of quality necessary to obtain the desired effect and potential health gains.[7] Effective coverage goes beyond contact coverage measurement by using not just the receipt of services by clients but also the quality of care received. Multiple quality domains can be considered, including inputs (service and equipment availability, training etc.), service delivery processes (e.g. compliance with protocols and standards of care), and outcomes (health benefits, client satisfaction etc.).[8] However, different studies have used various definitions and measurements for effective coverage, thus limiting the comparability among studies. To address this challenge, the recently proposed seven-step coverage framework begins with the target client population,

For peer review only - http://bmjopen.bmj.com/site/about/guidelines.xhtml

and following them through a hypothetical cascade of the losses of health benefits at different stages, including service contact (an individual in need contacts health service), input-adjusted coverage (contacts health service that is ready), intervention coverage (receives health services), quality-adjusted coverage (receives health service according to standard), user adherence -adjusted coverage (user adherence) and outcome-adjusted coverage (health gain achieved).[5 9]

Effective coverage has been found to be considerably lower than contact coverage for antenatal care (ANC),[10 11] family planning, and sick-child care in several low and middle income countries (LMICs).[11] Findings from LMICs also suggested low levels of availability, readiness, and coverage of obstetric services[12] and showed substantial reductions (20-39 percentage points [pp]) from contact coverage to effective coverage in facility birth.[13] Overall, a review of 36 studies showed large gaps between contact coverage and quality-adjusted coverage levels (10 to 38 pp) across the continuum of care for reproductive, maternal, newborn, and child health.[5]

Although recent studies have paid more attention to capturing the quality of care and quantifying the gaps between contact coverage and effective coverage across the continuum of care, studies on nutrition-related interventions are scarce. Previous studies mainly came from small-scale studies that focused on fortified foods where contact coverage was measured through exposure to a particular fortified food and quality-adjusted measures were based on regular consumption of the fortified food.[5] Recently, one study in Malawi showed that even though the utilization of ANC and facility delivery was high, women often did not receive nutrition-related interventions (such as iron interventions, nutrition counseling and breastfeeding counseling).[14]

For peer review only - http://bmjopen.bmj.com/site/about/guidelines.xhtml

Bangladesh has observed significant improvement in contact coverage in maternal and child health in the last decade,[15] yet substantial gaps remain in coverage of nutrition services, with less than 30% of women receiving all four key ANC nutrition interventions, and only 25% of sick children having their weight checked against a growth-chart.[16] Large gaps in facility readiness to provide nutrition interventions persist. Specifically, only around half of providers reported having received any training on nutrition, and only 13% of facilities possessed more than 5 of the 13 items deemed essential for service delivery for children.[16] Previous studies in Bangladesh have reported separately on contact coverage[17-19] and quality of services provided[16 20]. This paper aimed to adjust contact-based health coverage estimates in Bangladesh, taking into consideration the inputs required to deliver quality nutrition interventions across the continuum of care, specifically ANC and delivery for women and growth monitoring and curative care for young children.

For peer review only - http://bmjopen.bmj.com/site/about/guidelines.xhtml

DATA AND METHODS

Data sources

We used two sets of publicly-available nationally representative data: the 2014 Bangladesh Demographic and Health Survey (DHS)[21] and the 2014 Service Provision Assessment (SPA).[22] The DHS survey was a population-based household surveys that provided representative data on health and nutrition indicators at national and regional levels.[21] The survey was based on a two-stage stratified sample of households. In the first stage, 600 enumeration areas were selected with probability proportional to the enumeration area size. In the second stage of sampling, a systematic sample of 30 households on average was selected from a household list in each selected cluster by equal probability sampling. All women in selected households with a birth in the three years before the survey were interviewed about antenatal care and birth care for the most recent live birth. Child curative care questions were asked for all living child under 5 years in the household.

The SPA is a formal health facility-based survey designed to provide information on health service availability and readiness, with an emphasis on the delivery of reproductive, maternal, newborn and child health services.[22] The Bangladesh SPA included a standard set of survey instruments for a facility inventory and a health care provider interview. Facilities were assessed for the availability of health services, basic amenities, equipment, laboratory services, essential medicines, and human resources. During the facility inventory, the survey team attempted to observe elements of routinely offered care (such as weighing children, plotting child's weight on graph, etc.). Health facilities were sampled from a complete listing of 19,184 registered health facilities in the country, with the aim to provide the survey results separately

For peer review only - http://bmjopen.bmj.com/site/about/guidelines.xhtml

143 for the seven administrative divisions and different types of public facilities, NGO clinics/hospitals, and private hospitals. Small private clinics with less than 20 beds were excluded. Further details on the survey's sampling strategy were presented in the survey report.[22] The Bangladesh SPA did not include observation of selected client visits or exit interviews.

148 **Statistical analysis**

149 Input-adjusted coverage for nutrition intervention was calculated among individuals in need of care as the product of the coverage of services and the readiness of health facilities to provide quality care.

152 ***Coverage of services***

153 We estimated coverage of services based on DHS data, accounting for the DHS sampling weights. Coverage of ANC care was defined as the percentage of women 15–49 years old with at least one live birth in the three years preceding the survey who reported at least 4 ANC visits (one of those visits from a trained provider) for their most recent birth. Coverage of institutional delivery was defined as the percentage of women with a live birth in the 3 years preceding the survey who gave birth in a health facility. There was no consensus on coverage measurement for child growth monitoring. However, growth monitoring activities, such as height, weight and mid-upper arm circumference (MUAC) measurement, are intended to be a core part of the Integrated Management of Childhood Illness (IMCI) protocol that is followed during sick-child contacts[16] which has been shown to have an impact on childhood stunting.[23] Based on this, and given the lack of survey questions that explicitly ask respondents about growth monitoring services, coverage of growth monitoring was defined as the percentage of

For peer review only - http://bmjopen.bmj.com/site/about/guidelines.xhtml

all children under 5 years of age who sought treatment at a formal health facility. Coverage of sick-child care was defined as the percentage of children with any illness who sought treatment at a formal health facility.

Facility readiness to provide nutrition interventions

We analyzed facility readiness to deliver nutrition interventions based on six attributes as guided by the World Health Organization (WHO)'s Service Availability and Readiness Assessment Manual[24] including: 1) availability of nutrition interventions, 2) trained personnel, 3) guidelines, 4) equipment, 5) diagnostic capacity, and 6) medicines. We used the tracer indicators that are most relevant to capture selected nutrition interventions. The selected indicators for estimating input-adjusted coverage for nutrition interventions across continuum of care are presented in **Table 1**. During ANC, multiple nutrition interventions should occur; these include delivering iron folic acid (IFA) supplementation, weight monitoring for pregnant women, anemia management, blood pressure management, fetal growth monitoring and health and nutrition education. During birth, the main nutrition intervention is support for early breastfeeding practices. During early childhood, national protocols are for children to be weighed and to receive nutrition education, vitamin A supplementation, deworming, anemia management, and ORS and zinc for diarrhea.

We calculated the composite readiness score using a weighted additive approach, which weights the 6 domains equally within each measure. Previous studies have found that three methods (simple additive, weighted additive, or principal component analysis) for calculating quality indices yield generally comparable results. [25] The weighted additive method has been recommended[26] as easy to calculate and interpret. The domain scores were calculated by

For peer review only - http://bmjopen.bmj.com/site/about/guidelines.xhtml

averaging the indicators within each domain; domain scores were then summed and multiplied by 100 to create the total weighted additive scores which expressed as percent of total readiness. The calculated readiness score for a specific facility category was an average score of all facilities in the same category. We accounted for the SPA sampling weight when summarizing readiness scores.

Table 1: Definition of indicators for estimating input-adjusted coverage for nutrition interventions across continuum of care

Services	Associated nutrition interventions	Measure of need (denominator)	Measure of use (numerator)	Quality estimator
Antenatal care	• IFA supplementation<br>• Weight monitoring for pregnant women<br>• Anemia management<br>• Blood pressure management<br>• Fetal growth monitoring<br>• Health and nutrition education	Women 15–49 years old with at least one child under 3 years	Women 15–49 years old with at least one child under 3 years, whom for their most recent birth, reported at least 4 ANC visits (ANC4+)	Availability of services<br>• IFA supplementation<br>• Intermittent preventive treatment for malaria<br>• Weighing of clients<br>• Taking blood pressure<br>• Conducting group health education sessions<br>• Urine test for protein<br>• Blood test for anemia<br>• Measuring client's height<br>• Ultrasonography<br>Human resources<br>• Staff with any training on ANC<br>Guidelines<br>• National guidelines for ANC, visual aids for client education<br>Basic equipment<br>• Adult weighing scale<br>• Tape measure for fundal height<br>• Blood pressure apparatus<br>• Stethoscope<br>• Fetal stethoscope<br>Diagnostic capacity<br>• Hemoglobin,<br>• Urine protein<br>Essential medicines<br>• IFA tablets
Birth care	• Child birthweight measures<br>• Early breastfeeding practices	Women 15–49 years old with at least one	Women 15–49 years old with at least	Availability of services<br>• Kangaroo Mother Care<br>• Skin to skin contacts

For peer review only - http://bmjopen.bmj.com/site/about/guidelines.xhtml

Services	Associated nutrition interventions	Measure of need (denominator)	Measure of use (numerator)	Quality estimator
		child under 5 years	one child under 5 years, whom for their most recent birth, reported delivery in a health facility	• Initiation of breastfeeding within the first hour<br>• Do not give the newborn pre-lacteal liquids<br>• Weigh the newborn immediately<br>Human resources<br>• Staff with any training on IMPACT<br>Guidelines<br>• Guidelines on basic birth care BEmONC<br>• Guidelines on comprehensive birth care: CEmONC<br>Basic equipment<br>• Infant scale<br>• Manual or digital BP apparatus
Child growth monitoring	Child growth monitoring	All children alive between 0 and 59 months	All children who sought treatment from a medical provider	Availability of services<br>• Weighing the child<br>• Plotting child's weight on graph<br>Human resources<br>• Staff with any training on growth monitoring<br>Guidelines<br>• Guidelines for growth monitoring<br>Basic equipment:<br>• Scale<br>• Length or height board<br>• Tape for measuring head<br>• Growth chart
Sick child care	• Nutrition education<br>• Vitamin A supplementation<br>• Deworming<br>• Anemia management<br>ORS and zinc for diarrhea	All children alive between 0 and 59 months who had diarrhea or ARI in the last two weeks	All children who had diarrhea or ARI, for whom advice on treatment was sought from a medical provider	Availability of services<br>• Diagnose and/or treat child malnutrition<br>• Provide vitamin A supplementation to children<br>• Provide iron supplementation to children<br>• Provide zinc supplementation to children<br>• Provide deworming to children<br>• Provide group health education<br>Human resources<br>• Staff with any training on IMCI<br>Guidelines<br>• IMCI guideline: national guidelines for IMCI, IMCI chart booklet, IMCI card, other visual aids<br>Basic equipment<br>• Scale<br>Diagnostic capacity<br>• Hemoglobin<br>Essential medicines:<br>• ORS<br>• Albendazole/ mebendazole

For peer review only

For peer review only - http://bmjopen.bmj.com/site/about/guidelines.xhtml

Services	Associated nutrition interventions	Measure of need (denominator)	Measure of use (numerator)	Quality estimator
				• Iron tablet<br>• Vitamin A<br>• Zinc tablet/ zinc sulphate syrup.

Estimating input-adjusted coverage

We linked health facility and household survey data at the aggregate level, using an ecological linking method which has been shown a feasible and valid approach for estimating quality-adjusted effective coverage.[27] We disaggregated the coverage by type of facility where services were sought. In case women went to more than one type of facility for ANC, we used the highest level of facility for analyses. Facility types were harmonized between the DHS and SPA at 5 levels: district and upazila public facilities, union level public facilities, public community clinic, NGO clinic/hospital and private clinic/hospital (Supplemental Table 1).

Input-adjusted coverage was estimated at both the national and regional level (7 divisions), accounting for 5 types of facilities where the care was sought. At the regional level, the input-adjusted coverage was the summation of input-adjusted coverage of each type of facility that was constructed as the product of the coverage and readiness estimates:

$$\mathrm{IAC_r} = \sum_j C_{rj} * Q_{rj}$$

where IAC_r represented input-adjusted coverage in region r,

C_{rj} was the proportion of women/children who sought care in facility type j in region r,

And Q_{rj} was the average readiness score of facility type j in region r.

The national input-adjusted coverage was the summation of regional input-adjusted coverage weighted by the proportion of users in each region:

$$\mathrm{IAC_T} = \sum_r IAC_r * w_r$$

For peer review only - http://bmjopen.bmj.com/site/about/guidelines.xhtml

where w_r was the proportion of users in region r.

The variance of input-adjusted coverage estimates was calculated with an approximation procedure referred to as the "delta" method which has been used and described in detail in previous research.[28] Finally, we calculated the input-adjusted coverage by strata of place of residence, educational level and socio-economic (SES) quintiles. This analysis assumed that there was no systematic difference in facility readiness within region by the education or socioeconomic stratifiers (e.g. that within a given sub-national region, the average readiness of public community clinics used by highly educated women was the same as average readiness of public community clinics used by less educated women). Data analysis was performed using Stata version 16.0.

Patient and public involvement

Patients and/or the public were not involved in the design, or conduct, or reporting, or dissemination plans of this research.

RESULTS

The household survey included 17,863 women (out of 18,245 eligible women aged 15-49 years, a response rate of 98%), among them 4,488 women reporting a birth in the three years preceding the survey and 7,886 women with living children under 5 years. Health service coverage varied considerably between services, with low coverage for ANC4+ (28%), institutional delivery (38%), child growth monitoring (35%), but substantially higher for sick-child care (81%). The place of ANC and childcare by facility type was shown in **Supplemental Figure 1.**

For peer review only - http://bmjopen.bmj.com/site/about/guidelines.xhtml

The health facility survey included 1,548 facilities of 1,596 sampled (97% response rate); among them 1,493 facilities (96%) offering services on antenatal care, 586 (38%) offering services on delivery care, and 1,463 (92%) offering curative care for sick children. Government health facilities are the major providers of care (**Supplemental Figure 2)**. The availability of interventions across the continuum of care and inputs to deliver them is presented in **Table 2** for the national level. Routine availability of nutrition interventions varied. In ANC, around half of facilities took weight or measured blood pressure, a third conducted group health education sessions, and <10% conducted blood tests for anemia or urine tests for protein, and only 2% used ultrasonography to check on fetal growth. While health facilities performed well in providing supports for early breastfeeding during delivery care per facility self-report (70-98%), provision of services related to child growth assessment and nutritional management of illness was limited: only 41% of facilities had evidence of routine availability of measurement of child weight, with 27% plotting the child weight on a growth chart.

Overall, facilities lacked trained personnel, guidelines, equipment, medicines or supplies needed to provide high quality of care (**Table 2**). Of facilities offering each service, approximately half of facilities had staff with any training on ANC, child growth monitoring, and IMCI, but a lower proportion of facilities (39%) had staff with any training on integrated management of pregnancy and childbirth (IMPAC). Similarly, around half of facilities had national or other guidelines for ANC, growth monitoring or IMCI, but only 27% had guidelines for Basic Emergency Obstetric and Neonatal Care (BEmONC) or Comprehensive Emergency Obstetric and Neonatal Care (CEmONC). While a majority of facilities (>85%) had adult weighing scales, blood pressure monitors and stethoscopes, only 36% had tape measures for fundal

For peer review only - http://bmjopen.bmj.com/site/about/guidelines.xhtml

height and 15% had fetal stethoscopes. Child scales, length or height board and growth chart were only available in 60-70% of facilities that offered sick-child care. Regarding diagnostic capacity, 11% of facilities were able to test for hemoglobin and 18% for urine protein. Iron-folic acid supplements were available in 91% of facilities. Zinc or vitamin A supplementation was reported in three quarters of facilities and deworming for children was reported in almost all facilities. The availability of these items varied by division (Supplemental Table 2) and by types of facilities (Supplemental Table 3).

Table 2: Percentage of health facilities with structural input items and their readiness score

	Input items	% facilities with item available	95% CI
**Antenatal care (n=1493 facilities)**			
**Routine availability of nutrition interventions**	IFA/folic acid supplementation	81.28	75.67-86.90
	Intermittent preventive treatment for malaria (IPT)	5.06	3.18-6.93
	Weighing of clients	49.89	41.20-58.59
	Taking blood pressure	50.19	41.05-59.34
	Urine test for protein	8.56	4.61-12.50
	Blood test for anemia	6.58	3.05-10.10
	Ultrasonography	1.97	0.98-2.97
	Conducting group health education sessions	34.02	25.81-42.23
**Staff**	Staff with any training on ANC	48.75	43.24-54.26
**Guideline**	National or other guidelines for ANC	49.64	41.88-57.40
	Visual aids for client education	66.93	60.71-73.15
**Functional equipment**	Adult weighting scale	85.44	81.83-89.06
	Tape measure for fundal height	36.17	29.37-42.97
	Blood pressure apparatus	91.22	88.21-94.24
	Stethoscope	91.93	89.06-94.79
	Fetal stethoscope	15.25	10.77-19.74
**Diagnostics capacity**	Hemoglobin	10.57	7.15-13.99
	Urine protein	17.87	13.62-22.12
**Medicine**	Iron or folic acid tablets	91.41	88.18-94.64
Readiness score for ANC		***51.09***	***48.60-53.58***
**Birth care (n=586 facilities)**			
**Routine availability of nutrition interventions**	Kangaroo practice for low birth weight babies	44.11	32.79-55.42
	Skin to skin contacts	70.11	60.58-79.65
	Initiation of breastfeeding within the first hour	97.19	94.54-99.83
	Do not give the newborn prelacteal liquids	77.49	69.09-85.90
	Weigh the newborn immediately	70.08	61.53-78.63
**Staff**	Staff with any training on IMPAC	38.74	30.01-47.47
**Guideline**	Guidelines for BEmONC or CEmONC	27.18	17.28-37.09
**Functional equipment**	Infant scale	60.64	52.62-68.65
	Manual or digital BP apparatus	93.35	89.08-97.62

For peer review only

For peer review only - http://bmjopen.bmj.com/site/about/guidelines.xhtml

	Input items	% facilities with item available	95% CI
Readiness score for birth care		***53.68***	***49.19-58.16***
**Child growth monitoring (n=1463 facilities)**			
**Routine availability of nutrition interventions**	Weighing the child	40.92	31.83-50.00
	Plotting child's weight on graph	27.48	18.72-36.23
**Staff**	Staff with any training on growth monitoring	48.76	42.46-55.07
**Guideline**	Guidelines for growth monitoring	46.65	39.28-54.01
**Functional equipment**	Child scale (child or infant)	69.00	63.08-74.92
	Length or height board	59.16	51.64-66.67
	Tape for measuring head	30.67	24.48-36.86
	Growth chart	66.70	60.66-72.73
Readiness score for child growth monitoring		***45.44***	***41.29-49.60***
**Sick child care (n=1463 facilities)**			
**Routine availability of nutrition interventions**	Diagnose and/or treat child malnutrition	93.33	90.57-96.09
	Provide vitamin A supplementation to children	74.44	70.55-78.34
	Provide iron supplementation to children	64.03	57.31-70.75
	Provide zinc supplementation to children	75.05	70.19-79.90
	Provide deworming to children	97.44	96.43-98.46
	Providing group health education	35.45	26.30-44.61
	Weighing the child	40.92	31.83-50.00
	Plotting child's weight on graph	27.48	18.72-36.23
**Staff**	Staff with any training on IMCI	53.85	47.36-60.33
**Guideline**	Guidelines for IMCI	50.84	44.29-57.39
	IMCI chart booklet	53.35	47.13-59.56
	IMCI mother's cards (IMCI card)	39.48	33.50-45.45
	Other visual aids for teaching caretakers	56.23	50.36-62.09
**Functional equipment**	Child scale (child or infant)	60.80	54.16-67.44
**Diagnostics capacity**	Hemoglobin	10.34	6.85-13.84
**Medicine**	ORS	63.59	58.14-69.04
	Albendazole/mebendazole	87.59	83.68-91.50
	Iron tablet	54.35	46.15-62.56
	Vitamin A	66.52	60.60-72.44
	Zinc tablet/zinc sulphate syrup	65.75	59.92-71.57
Readiness score for sick child care		***50.20***	***46.99-53.40***

[1]Survey team assessing whether they see these practices in general in the facility at the time of the visit.
ANC: antenatal care, BEmONC: Basic Emergency Obstetric and Neonatal Care, CEmONC: Comprehensive Emergency Obstetric and Neonatal Care, IMCI: integrated management of childhood illness, IMPAC: integrated management of pregnancy and childbirth, ORS: Oral rehydration salts.

Readiness scores were calculated at the national (**Table 2**) and regional levels and by facility type (**Figure 1**). Overall, facilities demonstrated 51% of the expected resources to provide nutrition interventions during ANC, 54% for delivery care, 45% for growth monitoring, and approximately half the required resources for child health services. Readiness scores varied

For peer review only - http://bmjopen.bmj.com/site/about/guidelines.xhtml

by regions and depending on the service. Rangpur performed the best overall, and Sylhet showed high readiness for ANC but lowest on delivery care and average on others (**Figure 1**). District and upazila public facilities had the highest readiness scores across the continuum of care, whereas lower-level facilities (union level and public community) were much less prepared. For example, district public hospitals had the highest readiness score, with 73% of the expected capacity to provide nutrition interventions during ANC care services, but public community clinics had a readiness score of only 49%.

<Insert Figure 1 here>

Figure 2 plots health service coverage against facilities' readiness score in each region of the country. For ANC4+, the regional averages of coverage and readiness fell mostly in quadrant III for all regions, indicating both low coverage (ranged between 18-36%) and moderate readiness (between 49-56%) (Table 3), thus the priority is improving coverage as well as quality of nutrition interventions for ANC care. In contrast, the priority for delivery care clearly lies in improving coverage: most regions had low access (less than 50% except Khulna) but had higher readiness (48-68%). For child growth monitoring, all regions showed low coverage (30-39%); Dhaka had the lowest readiness (38%) while other regions had a higher but wide range of readiness (45-56%). For sick-child care, regions had similar levels of high coverage (>75%) but showed lower and highly variable readiness.

<Insert Figure 2 here>

Taking into account the readiness of facilities to provide nutrition interventions and the use of services, we estimated the input-adjusted coverage at the national and regional level (**Table 3 and Figure 3**). At the national level, input-adjusted coverage was 18% for ANC4+, 24%

For peer review only - http://bmjopen.bmj.com/site/about/guidelines.xhtml

for delivery care, 20% for child growth monitoring and 52% for sick-child care. The gaps between contact and input-adjusted coverage ranged between 10-39 percentage points (pp).

<Insert Figure 3 here>

Table 3: Estimated regional and national input-adjusted coverage of nutrition interventions across continuum of care, Bangladesh 2014

	Contact coverage	Readiness score	Input-adjusted coverage	
			Estimate	95% CI
**Antenatal care 4+**				
Barisal	22.79	49.08	14.57	10.95-19.12
Chittagong	24.35	49.54	15.67	12.80-19.05
Dhaka	31.19	50.46	19.87	16.54-23.68
Khulna	36.21	50.05	23.03	18.83-27.85
Rajshahi	24.36	49.25	15.92	12.44-20.13
Rangpur	34.44	56.02	23.20	18.34-28.89
Sylhet	18.44	56.48	12.18	8.70-16.80
**Overall**	**28.06**	**51.09**	**18.11**	**16.50-19.84**
**Birth care**				
Barisal	30.38	48.82	18.47	13.85-24.20
Chittagong	35.49	50.37	22.68	18.70-27.23
Dhaka	40.91	53.47	25.53	21.22-30.37
Khulna	54.96	52.28	35.56	28.50-43.32
Rajshahi	39.23	52.05	24.66	20.64-29.18
Rangpur	34.52	68.12	23.19	18.58-28.54
Sylhet	22.87	47.95	14.35	11.06-18.43
**Overall**	**37.73**	**53.68**	**23.92**	**21.86-26.10**
**Child growth monitoring**				
Barisal	32.77	49.23	20.64	16.74-25.18
Chittagong	36.78	44.83	22.47	18.06-27.58
Dhaka	33.87	38.94	20.73	17.79-24.01
Khulna	29.79	44.83	17.65	14.79-20.92
Rajshahi	35.34	46.92	7.72	6.52-9.12
Rangpur	36.99	56.26	25.27	19.50-32.08
Sylhet	38.51	40.44	22.74	16.85-29.95
**Overall**	**35.05**	**45.44**	**20.18**	**18.45-22.03**
**Sick child care**				
Barisal	76.83	53.54	50.36	41.56-59.13
Chittagong	82.92	44.80	51.64	42.40-60.77
Dhaka	80.95	45.59	50.60	42.80-58.38
Khulna	77.28	45.96	47.52	38.84-56.34
Rajshahi	78.53	52.47	51.23	40.45-61.90
Rangpur	82.69	64.50	57.65	48.52-66.29
Sylhet	84.38	53.72	53.21	42.61-63.53
**Overall**	**81.20**	**50.20**	**51.68**	**47.75-55.58**

For peer review only - http://bmjopen.bmj.com/site/about/guidelines.xhtml

When examining the inequality gaps, we found that input-adjusted coverage varied by as much as 8-24 pp between regions within the country (Figure 3). Inequality in input-adjusted coverage was large during pregnancy and delivery (13-17 pp between urban and rural areas, 14-16 pp between low and high education, and 23-35 pp between the rich and the poor), but narrower for child growth monitoring and sick-child care (<2 pp) (Figure 4).

<Insert Figure 4 here>

DISCUSSION

Improving both coverage and quality of healthcare services is essential to achieving universal health coverage and achieving the third Sustainable Development Goal of ensuring healthy lives and promoting well-being for all at all ages.[29] Our study is one of very few to develop and apply nutrition-related metrics of effective coverage across the continuum of care by incorporating service readiness of nutrition interventions into coverage estimates of ANC and post-delivery care. Our findings showed low levels of coverage across the continuum of care (28-38%), except for sick-child care (81%); we also found moderate levels of facility readiness to provide nutrition interventions (41-74%). After adjustment for readiness, the input-adjusted coverage was found to be 10-40 pp lower than the contact coverage, and there was high level of inequality between regions within the country, residential areas, education and SES status for ANC and birth care, but less so for sick-child care. Because our estimates focused on inputs to care and did not capture how often these inputs were provided in adherence with evidence-based protocols, they should be considered an upper limit of the quality of nutrition care actually received.

For peer review only - http://bmjopen.bmj.com/site/about/guidelines.xhtml

The large gap between contact and adjusted coverage in our study was aligned with a review of 36 studies finding a range of 10-38 pp reductions between crude coverage and quality-adjusted coverage across the continuum of care.[5] These findings indicated that women who sought care in a health facility did not necessarily receive the quality of care needed for them and their children. Our findings provided insight into health system challenges and indicate potential priorities for improvement.

For ANC, the priority is ensuring that all four contacts are provided by a trained provider, while improving coverage and quality simultaneously to reach high levels of effective coverage for nutrition interventions. We found a low contact coverage of 4+ ANC (28%), with evidence of inequities by wealth, education and place of residence. Evidence from a systematic review showed that both demand-side (such as maternal education, spousal education, marital status, women's employment, autonomy, cultural beliefs, media exposure, and household income) and supply-side factors (such as service availability and cost) influence ANC uptake.[30 31] Financial and transport constraints may be a greater barrier for women in remote and poorer areas.[32 33] Interventions such as mobile health technologies have been found to improve ANC attendance,[34] but improving coverage requires targeted investments to reach underserved communities. We also found low readiness of the health system to provide nutritional interventions during ANC (51%) which is consistent with a previous study in Bangladesh.[16] Our findings suggested that facility readiness can be improved by ensuring the availability of trained providers and necessary equipment, as well as strengthening diagnostic capacity.

For birth care, the priority clearly lies in improving coverage of facility-based deliveries while also improving readiness and provider compliance with labor management protocols to

For peer review only - http://bmjopen.bmj.com/site/about/guidelines.xhtml

increase support for early initiation of breastfeeding. Although the trend of institutional delivery has increased over time from 29% in 2011[35 36] to 38% at the time of the survey in 2014, nearly two third of all births still take place at home. Women's educational interventions and community-level mobilization may increase the coverage of institutional delivery.[37 38] In terms of quality, facilities have moderate readiness (54%) to provide nutrition interventions, particularly activities related to early initiation of breastfeeding, but additional efforts are needed to improve the overall quality of service for facility-based delivery, including training staff, providing guidelines, emergency transport and necessary equipment.[39]

For care for children under 5, the priority is improving both coverage and quality of nutrition interventions for child growth monitoring. While community-based management of acute malnutrition activities such as screening and growth monitoring has been prioritized under the Bangladesh National Nutrition Services,[40] there is little data on the coverage and uptake of these interventions. Growth monitoring activities are part of the IMCI protocol that is to be followed during sick-child contacts, but implementation is poor[16] and their reach across the population is limited by the reach of public health facilities and by inclusion in sick child care rather than in well-child care.[41] This suggests the need for widely available services to deliver preventive nutrition services, not only to identify malnourished children, but to detect growth faltering early through regular growth monitoring and counseling for all children. Coverage can be increased by generating community awareness about the need for these services and generating demand. To ensure quality, trained staff should be available and have the necessary equipment to take anthropometric measurements, correctly use growth charts, and counsel

For peer review only

For peer review only - http://bmjopen.bmj.com/site/about/guidelines.xhtml

caregivers. Other elements of overall sick-child care quality should be improved by ensuring that staffs are trained on IMCI, and that guidelines, equipment and medicine are available.

The inequality in adjusted coverage highlights targets for intervention according to administrative division and populations served. There is a clear variability in the adjusted coverage of indicators across administrative divisions, with some divisions (Khulna and Rangpur) performing better overall and others (Sylhet) performing poorly overall. For Sylhet specifically, poor performance may be because of its terrain, which makes many areas hard to reach. However, lessons should be shared from the successes of high-performing divisions such as Khulna and Rangpur, and focused strategic planning and action could help reduce regional disparities. Input-adjusted coverage also showed inequities by place of residence, education, and socioeconomic status. However, the size of these inequalities varied where the gaps were larger in the case of ANC and delivery, but smaller for child growth monitoring and sick-child care. Our findings of inequalities in coverage during pregnancy and delivery align with previous research finding that urban residence, higher education attainment (for the respondent and spouse), and higher wealth status significantly predict utilization of institutional delivery services[36] and 4+ ANC exposure[17]. Thus, there is a special need to specifically target underserved populations.

Investing in strengthening effective coverage of nutrition interventions is particularly important for a resource-poor country like Bangladesh which still carries a large burden of poor maternal and child health and nutrition. Despite consistent improvements in coverage of key nutrition service delivery platforms/contacts i.e. ANC and child health services, the health sector can do much more to strengthen nutrition service delivery in Bangladesh. The rapid

For peer review only - http://bmjopen.bmj.com/site/about/guidelines.xhtml

growing private sector in the context of an ineffective regulatory environment has contributed to the lack of standardized practice in this setting [42]. Investing in the quality of public services, therefore, could bring clients back to use public services and ensure better coverage of higher quality care. The challenge for governments scaling proven interventions at desired coverage *and* quality in such settings, however, is multidimensional. Depending on the type of contact and the intervention, it is likely that investments in strengthening the public sector platforms require both structural and process fixes. In concert with this, the government's stewardship in establishing functioning regulatory and accountability mechanisms for private sector service delivery can help to transform contact coverage into effective coverage.

Previous research has shown that relying only on facility-based health service delivery may not be appropriate for preventive service delivery in Bangladesh. In the current policy set-up, only a subset of children is reached by services provided during sick child visits and coverage of ANC for women continues to be sub-optimal [16]. To improve overall service coverage and quality, there is a need to strengthen existing services, while also exploring other avenues to improve access and coverage of key nutrition interventions through new service delivery contacts. Potential approaches include the provision of nutrition interventions in a preventative setting or via outreach activities, including home visits by frontline workers, community-based events for nutrition service interventions during pregnancy, or well-child services held at the community-level. Evidence exists about the use of community-based frontline workers to support infant and young child feeding practices and maternal nutrition in multiple countries including Bangladesh [43-46]. These, and other options, should continue to be

For peer review only - http://bmjopen.bmj.com/site/about/guidelines.xhtml

explored in Bangladesh to ensure broad scale coverage of high quality nutrition interventions for women and children.

Our study has several strengths. Studies using data from household surveys often report contact coverage estimates that are limited by the respondent recall, but do not capture the content of these services nor their quality. Our study has reported both the services readiness (which was estimated from health facility data), and the contact coverage (which was drawn from household survey data) and combined these two to estimate input-adjusted coverage. The household surveys and facility data were collected in the same year, thus allowing us to compare facility readiness and services received at the same time for child health services. These data were combined using valid ecological linking method[27] by using facility type and disaggregating by divisions and urban/rural location. Variance and precision of coverage indicators were derived from linked data using the recommended delta method[28]. By including nutrition interventions for multiple populations (urban vs. rural; varying levels of education and wealth quintile) in our analysis, we provide a potential tool to assess the health promotion capacity of the health system by highlighting underserved populations.

We acknowledge some of the limitations of this analysis. *First,* although we included various facility levels, the exclusion of small private clinics or hospitals with less than 20 beds from the SPA could lead to an overestimation of quality for private sector users as larger facilities tend to have higher readiness than smaller ones. *Second,* contact coverage was estimated based on women's recall; however, women may not remember exactly where they received services, particularly if they went to more than one type of facility. We have addressed this by restricting the recall period of 3 years for ANC and birth to minimize the recall error.

For peer review only - http://bmjopen.bmj.com/site/about/guidelines.xhtml

433 *Third*, the assumption of no systematic difference in facility readiness within region by the education or socioeconomic stratifies may affect inequality estimates. In reality, differences in facilities in the same category, discrimination in provision care of underserved groups, and lack of individual capacity to extract value from the healthcare system may lead to systematic differences in quality experienced by women of different socioeconomic groups in the same tier of facilities,[47] leading to higher inequalities than are shown here. Finally, although the SPA survey is quite comprehensive, it does not capture every aspect to measure facility readiness to provide nutrition interventions (such as calcium or food supplements for pregnant women, or IFA and food supplements for children), nor do the data available allow calculation of the care cascade from need for services through health benefits, including quality-adjusted coverage, user adherence -adjusted coverage, or outcome-adjusted coverage[5 9]. Additional data on beneficiary side such as client observations, exit interviews or client perceptions are needed to understand the flow of services provided, services received and whether they can translate into changes in behavior or practices.

CONCLUSION

Sustainable Development Goal 3 calls for achieving universal health coverage, which requires successfully covering underserved populations with healthcare services as well as ensuring that the quality of services is adequate. Nutrition interventions should be an integral part of health services for mothers and children; however, we find that nutrition input-adjusted coverage was suboptimal and varied sub-nationally and across the continuum of care in

For peer review only - http://bmjopen.bmj.com/site/about/guidelines.xhtml

454 Bangladesh. Special efforts are needed to improve the reach as well as the quality of health and

455 nutrition services to achieve the SDGs.

For peer review only

For peer review only - http://bmjopen.bmj.com/site/about/guidelines.xhtml

FIGURE LEGENDS

Figure 1: Readiness score of facilities to provide nutrition interventions, by division (A) and types of facility (B), Bangladesh 2014

A. By division

B. By facility type

Figure 2: Regional readiness score of facilities to provide nutrition interventions versus coverage, Bangladesh 2014

Figure 3: Input-adjusted coverage of nutrition interventions by region, Bangladesh 2014

Figure 4: Inequality in input-adjusted coverage of nutrition interventions, Bangladesh 2014

SUPPLEMENTARY FILES

Supplementary table 1: Harmonized facility categories and reported categories in SPA and DHS

Supplemental Table 2: Percentage of health facilities with structural input items, by division

Supplemental Table 3: Percentage of health facilities with structural input items, by facility type

Supplemental Figure 1: Place of ANC and child care by facility type, Bangladesh 2014

Supplemental Figure 2: Distribution of facilities with ANC and sick child care by facility type, Bangladesh 2014

For peer review only - http://bmjopen.bmj.com/site/about/guidelines.xhtml

For peer review only

AUTHOR CONTRIBUTIONS

PHN conceived the manuscript, conducted the statistical analysis, wrote significant sections of the manuscript and revised the manuscript.

LQK conducted the statistical analysis, prepared tables and figures for the manuscript.

PP conducted the literature review, wrote significant sections of the manuscript and revised the manuscript.

MB supported data interpretation, reviewed and edited the manuscript.

PM supported data interpretation, reviewed and edited the manuscript.

EP supported data interpretation, reviewed and edited the manuscript.

HHL conceived the manuscript, advised on statistical analysis, supported data interpretation, and revised the manuscript.

All authors read and approved the final submitted manuscript.

FUNDING: Bill & Melinda Gates Foundation through POSHAN, led by International Food Policy Research Institute. Grant number: OPP50838

CONFLICT OF INTEREST STATEMENT: Authors declare no conflicts of interest.

DATA AVAILABILITY STATEMENT: All relevant data are within the manuscript and its Supporting Information files. Additional original data can be found at the DHS website https://dhsprogram.com/data/available-datasets.cfm.

For peer review only

For peer review only - http://bmjopen.bmj.com/site/about/guidelines.xhtml

496 **ETHICAL APPROVAL**

497 The survey and its procedures were properly reviewed and approved by the authority of the
498 National Institute of Population Research and Training (NIPORT) of the Ministry of Health and
499 Family Welfare. This study used secondary data analyses; thus, ethics approval was not
500 required.

For peer review only

For peer review only - http://bmjopen.bmj.com/site/about/guidelines.xhtml

REFERENCES

1. Chou VB, Walker N, Kanyangarara M. Estimating the global impact of poor quality of care on maternal and neonatal outcomes in 81 low- and middle-income countries: A modeling study. *PLoS Med* 2019;16(12):e1002990. doi: 10.1371/journal.pmed.1002990 [published Online First: 2019/12/19]
2. Bhutta ZA, Das JK, Rizvi A, et al. Evidence-based interventions for improvement of maternal and child nutrition: what can be done and at what cost? *Lancet* 2013;382(9890):452-77. doi: 10.1016/S0140-6736(13)60996-4 [published Online First: 2013/06/12]
3. Gillespie S, Menon P, Heidkamp R, et al. Measuring the coverage of nutrition interventions along the continuum of care: time to act at scale. *BMJ Glob Health* 2019;4(Suppl 4):e001290. doi: 10.1136/bmjgh-2018-001290 [published Online First: 2019/07/13]
4. Countdown to 2030 Collaboration. Countdown to 2030: tracking progress towards universal coverage for reproductive, maternal, newborn, and child health. *Lancet* 2018;391(10129):1538-48. doi: 10.1016/S0140-6736(18)30104-1 [published Online First: 2018/02/06]
5. Amouzou A, Leslie HH, Ram M, et al. Advances in the measurement of coverage for RMNCH and nutrition: from contact to effective coverage. *BMJ Glob Health* 2019;4(Suppl 4):e001297. doi: 10.1136/bmjgh-2018-001297 [published Online First: 2019/07/13]
6. Shengelia B, Tandon A, Adams OB, et al. Access, utilization, quality, and effective coverage: an integrated conceptual framework and measurement strategy. *Soc Sci Med* 2005;61(1):97-109. doi: 10.1016/j.socscimed.2004.11.055 [published Online First: 2005/04/26]
7. Boerma T, AbouZahr C, Evans D, et al. Monitoring intervention coverage in the context of universal health coverage. *PLoS Med* 2014;11(9):e1001728. doi: 10.1371/journal.pmed.1001728 [published Online First: 2014/09/23]
8. Donabedian A. Evaluating the quality of medical care. 1966. *Milbank Q* 2005;83(4):691-729. doi: 10.1111/j.1468-0009.2005.00397.x [published Online First: 2005/11/11]
9. Marsh AD, Muzigaba M, Diaz T, et al. Effective coverage measurement in maternal, newborn,child, and adolescent health and nutrition: progress, future prospects, and implications for quality health systems. *Lancet Glob Health* 2020;8:e730–36.
10. Kanyangarara M, Munos MK, Walker N. Quality of antenatal care service provision in health facilities across sub-Saharan Africa: Evidence from nationally representative health facility assessments. *J Glob Health* 2017;7(2):021101. doi: 10.7189/jogh.07.021101 [published Online First: 2017/11/23]
11. Leslie HH, Malata A, Ndiaye Y, et al. Effective coverage of primary care services in eight high-mortality countries. *BMJ Glob Health* 2017;2(3):e000424. doi: 10.1136/bmjgh-2017-000424 [published Online First: 2018/04/11]
12. Kanyangarara M, Chou VB, Creanga AA, et al. Linking household and health facility surveys to assess obstetric service availability, readiness and coverage: evidence from 17 low- and middle-income countries. *J Glob Health* 2018;8(1):010603. doi: 10.7189/jogh.08.010603 [published Online First: 2018/06/05]
13. Wang W, Mallick L, Allen C, et al. Effective coverage of facility delivery in Bangladesh, Haiti, Malawi, Nepal, Senegal, and Tanzania. *PLoS One* 2019;14(6):e0217853. doi: 10.1371/journal.pone.0217853 [published Online First: 2019/06/12]
14. Joseph NT, Piwoz E, Lee D, et al. Examining coverage, content, and impact of maternal nutrition interventions: the case for quality-adjusted coverage measurement. *Journal of Global Health* 2020;10: 010501
15. NIPORT. Bangladesh Demographic and Health Survey 2017-18: Key Indicators. Dhaka, Bangladesh, and Rockville, Maryland, USA: NIPORT and ICF, 2019.

For peer review only - http://bmjopen.bmj.com/site/about/guidelines.xhtml

16. Billah SM, Saha KK, Khan ANS, et al. Quality of nutrition services in primary health care facilities: Implications for integrating nutrition into the health system in Bangladesh. *PLoS One* 2017;12(5):e0178121. doi: 10.1371/journal.pone.0178121 [published Online First: 2017/05/26]
17. Islam MM, Masud MS. Health care seeking behaviour during pregnancy, delivery and the postnatal period in Bangladesh: Assessing the compliance with WHO recommendations. *Midwifery* 2018;63:8-16. doi: 10.1016/j.midw.2018.04.021 [published Online First: 2018/05/15]
18. Ali N, Sultana M, Sheikh N, et al. Predictors of Optimal Antenatal Care Service Utilization Among Adolescents and Adult Women in Bangladesh. *Health Serv Res Manag Epidemiol* 2018;5:2333392818781729. doi: 10.1177/2333392818781729 [published Online First: 2018/08/08]
19. Anwar I, Nababan HY, Mostari S, et al. Trends and inequities in use of maternal health care services in Bangladesh, 1991-2011. *PLoS One* 2015;10(3):e0120309. doi: 10.1371/journal.pone.0120309 [published Online First: 2015/03/24]
20. Biswas TK, Sujon H, Rahman MH, et al. Quality of maternal and newborn healthcare services in two public hospitals of Bangladesh: identifying gaps and provisions for improvement. *BMC Pregnancy Childbirth* 2019;19(1):488. doi: 10.1186/s12884-019-2656-1 [published Online First: 2019/12/12]
21. NIPORT. National Institute of Population Research and Training (NIPORT), Mitra and Associates, and ICF International. Bangladesh Demographic and Health Survey 2014. Dhaka, Bangladesh, and Rockville, Maryland, USA: NIPORT, Mitra and Associates, and ICF International, 2016.
22. NIPORT. National Institute of Population Research and Training (NIPORT), Associates for Community and Population Research (ACPR), and ICF International. 2016. Bangladesh Health Facility Survey 2014. Dhaka, Bangladesh: NIPORT, ACPR, and ICF International, 2016.
23. Arifeen SE, Hoque DM, Akter T, et al. Effect of the Integrated Management of Childhood Illness strategy on childhood mortality and nutrition in a rural area in Bangladesh: a cluster randomised trial. *Lancet* 2009;374(9687):393-403. doi: 10.1016/S0140-6736(09)60828-X [published Online First: 2009/08/04]
24. WHO. Service Availability and Readiness Assessment (SARA). An annual monitoring system for service delivery. Reference Manual. Geneva, Switzerland: World Health Organization 2013.
25. Sheffel A, Zeger S, Heidkamp R, et al. Development of summary indices of antenatal care service quality in Haiti, Malawi and Tanzania. *BMJ Open* 2019;9(12):e032558. doi: 10.1136/bmjopen-2019-032558 [published Online First: 2019/12/05]
26. Mallick L, Temsah G, Wang W. Comparing summary measures of quality of care for family planning in Haiti, Malawi, and Tanzania. *PLoS One* 2019;14(6):e0217547. doi: 10.1371/journal.pone.0217547 [published Online First: 2019/06/08]
27. Munos MK, Maiga A, Do M, et al. Linking household survey and health facility data for effective coverage measures: a comparison of ecological and individual linking methods using the Multiple Indicator Cluster Survey in Cote d'Ivoire. *J Glob Health* 2018;8(2):020803. doi: 10.7189/jogh.08.020803 [published Online First: 2018/11/10]
28. Sauer SM, Pullum T, Wang W, et al. Variance estimation for effective coverage measures: A simulation study. *Journal of Glob Health (in press)* 2020
29. UNDP. Sustainable Development Goals Geneva, Switzerland: United Nations; 2015 [Available from: https://sustainabledevelopment.un.org/sdg3 accessed 12/03 2020.
30. Simkhada B, Teijlingen ER, Porter M, et al. Factors affecting the utilization of antenatal care in developing countries: systematic review of the literature. *J Adv Nurs* 2008;61(3):244-60. doi: 10.1111/j.1365-2648.2007.04532.x [published Online First: 2008/01/17]

For peer review only - http://bmjopen.bmj.com/site/about/guidelines.xhtml

31. Haider MR, Qureshi ZP, Khan MM. Effects of women's autonomy on maternal healthcare utilization in Bangladesh: Evidence from a national survey. *Sex Reprod Healthc* 2017;14:40-47. doi: 10.1016/j.srhc.2017.09.002 [published Online First: 2017/12/03]
32. Haque MA, Dash SK, Chowdhury MA. Maternal health care seeking behavior: the case of Haor (wetland) in Bangladesh. *BMC Public Health* 2016;16:592. doi: 10.1186/s12889-016-3296-2 [published Online First: 2016/07/20]
33. Islam MR, Odland JO. Determinants of antenatal and postnatal care visits among Indigenous people in Bangladesh: a study of the Mru community. *Rural Remote Health* 2011;11(2):1672. [published Online First: 2011/07/01]
34. Watterson JL, Walsh J, Madeka I. Using mHealth to Improve Usage of Antenatal Care, Postnatal Care, and Immunization: A Systematic Review of the Literature. *Biomed Res Int* 2015;2015:153402. doi: 10.1155/2015/153402 [published Online First: 2015/09/18]
35. NIPORT. National Institute of Population Research and Training (NIPORT), Mitra and Associates, and ICF International. Bangladesh Demographic and Health Survey 2011. Dhaka, Bangladesh, and Rockville, Maryland, USA: NIPORT, Mitra and Associates, and ICF International, 2013.
36. Yaya S, Bishwajit G, Ekholuenetale M. Factors associated with the utilization of institutional delivery services in Bangladesh. *PLoS One* 2017;12(2):e0171573. doi: 10.1371/journal.pone.0171573 [published Online First: 2017/02/14]
37. Sharma BB, Jones L, Loxton DJ, et al. Systematic review of community participation interventions to improve maternal health outcomes in rural South Asia. *BMC Pregnancy Childbirth* 2018;18(1):327. doi: 10.1186/s12884-018-1964-1 [published Online First: 2018/08/12]
38. Lassi ZS, Bhutta ZA. Community-based intervention packages for reducing maternal and neonatal morbidity and mortality and improving neonatal outcomes. *Cochrane Database Syst Rev* 2015(3):CD007754. doi: 10.1002/14651858.CD007754.pub3 [published Online First: 2015/03/25]
39. NIPORT. National Institute of Population Research and Training (NIPORT), Associates for Community and Population Research (ACPR), and ICF International. 2020. Bangladesh Health Facility Survey 2017. Dhaka, Bangladesh: NIPORT, ACPR, and ICF International, 2020.
40. MoH&FW. Operational Plan For 'National Nutrition Services' July 2011-June 2016. In: Welfare MoHaF, ed., 2011.
41. Saha KK, Billah SM, Menon P, et al. Bangladesh National Nutrition Services: assessment of implementation status: The World Bank, 2015.
42. Ahmed SM, Evans TG, Standing H, et al. Harnessing pluralism for better health in Bangladesh. *Lancet* 2013;382(9906):1746-55. doi: 10.1016/S0140-6736(13)62147-9 [published Online First: 2013/11/26]
43. Kim SS, Nguyen PH, Tran LM, et al. Large-Scale Social and Behavior Change Communication Interventions Have Sustained Impacts on Infant and Young Child Feeding Knowledge and Practices: Results of a 2-Year Follow-Up Study in Bangladesh. *J Nutr* 2018;148(10):1605-14. doi: 10.1093/jn/nxy147 [published Online First: 2018/09/01]
44. Menon P, Nguyen PH, Saha KK, et al. Combining Intensive Counseling by Frontline Workers with a Nationwide Mass Media Campaign Has Large Differential Impacts on Complementary Feeding Practices but Not on Child Growth: Results of a Cluster-Randomized Program Evaluation in Bangladesh. *J Nutr* 2016;146(10):2075-84. doi: 10.3945/jn.116.232314
45. Menon P, Nguyen PH, Saha KK, et al. Impacts on Breastfeeding Practices of At-Scale Strategies That Combine Intensive Interpersonal Counseling, Mass Media, and Community Mobilization: Results of Cluster-Randomized Program Evaluations in Bangladesh and Viet Nam. *PLoS Med* 2016;13(10):e1002159. doi: 10.1371/journal.pmed.1002159

For peer review only - http://bmjopen.bmj.com/site/about/guidelines.xhtml

46. Nguyen PH, Kim SS, Sanghvi T, et al. Integrating Nutrition Interventions into an Existing Maternal, Neonatal, and Child Health Program Increased Maternal Dietary Diversity, Micronutrient Intake, and Exclusive Breastfeeding Practices in Bangladesh: Results of a Cluster-Randomized Program Evaluation. *J Nutr* 2017;147(12):2326-37. doi: 10.3945/jn.117.257303 [published Online First: 2017/10/13]
47. Sharma J, Leslie HH, Kundu F, et al. Poor Quality for Poor Women? Inequities in the Quality of Antenatal and Delivery Care in Kenya. *PLoS One* 2017;12(1):e0171236. doi: 10.1371/journal.pone.0171236 [published Online First: 2017/02/01]

For peer review only

For peer review only - http://bmjopen.bmj.com/site/about/guidelines.xhtml

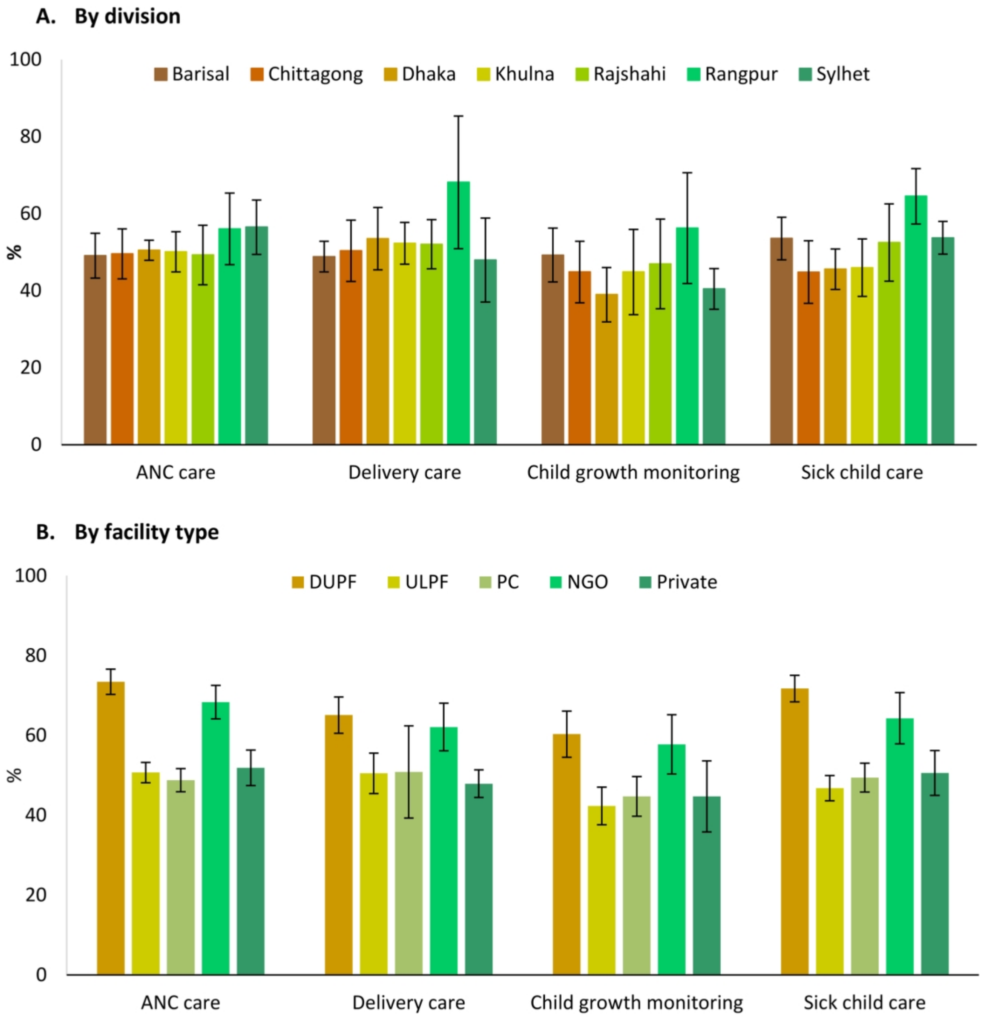

Figure 1

82x86mm (600 x 600 DPI)

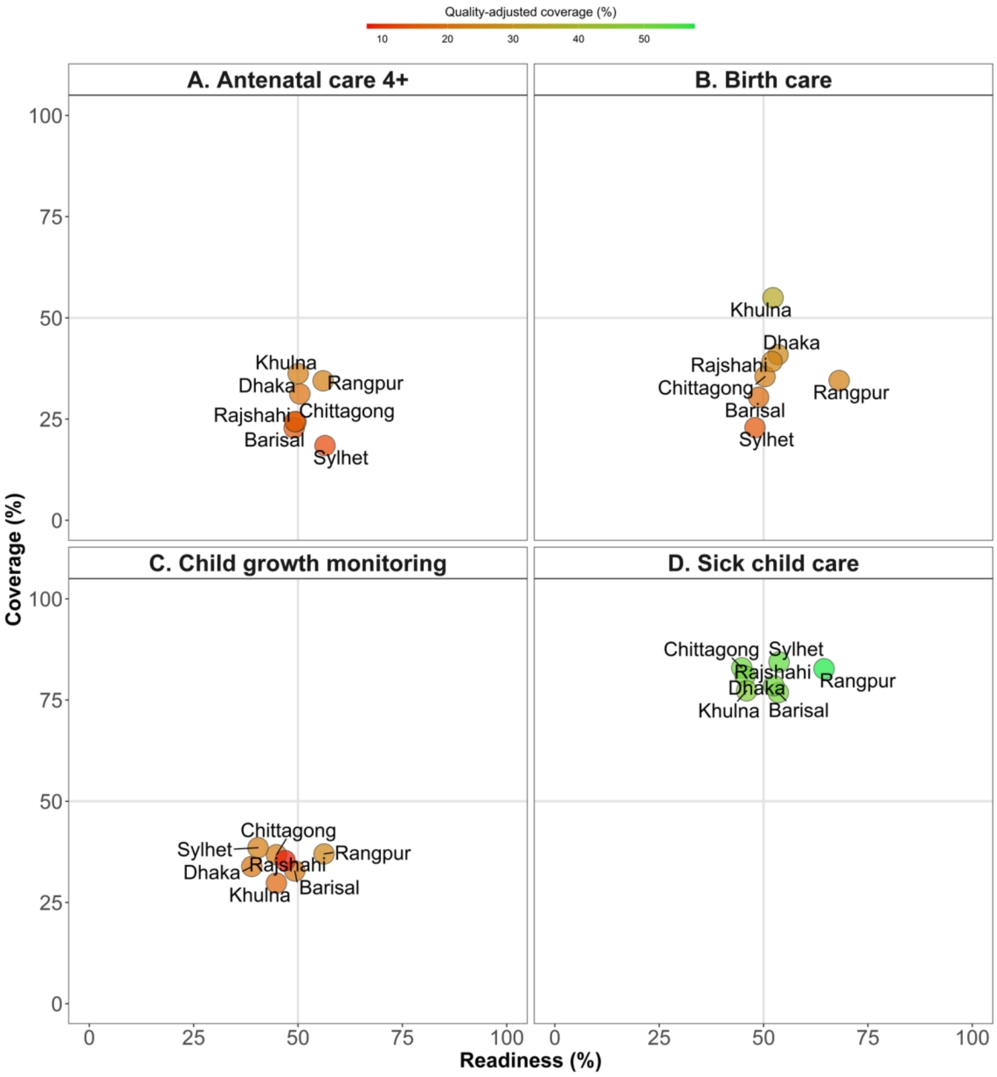

Figure 2

152x165mm (300 x 300 DPI)

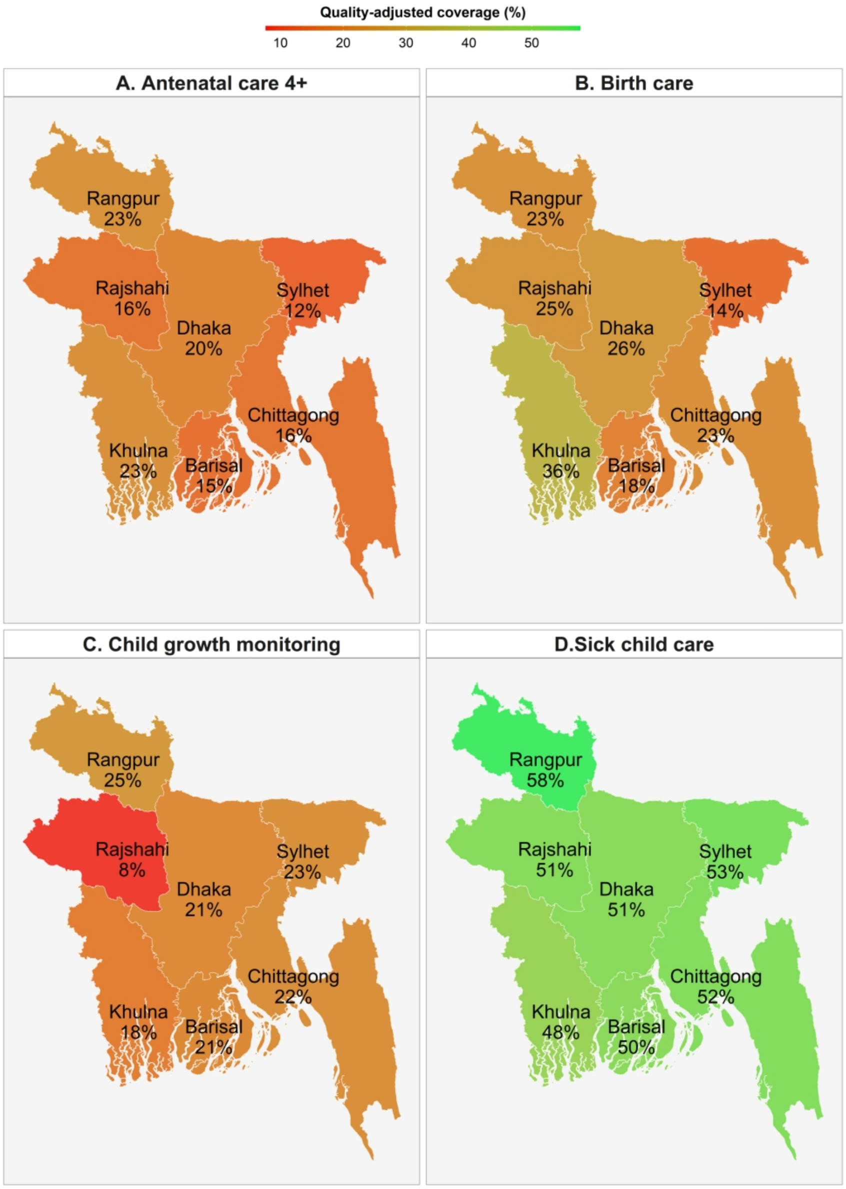

Figure 3

101x127mm (600 x 600 DPI)

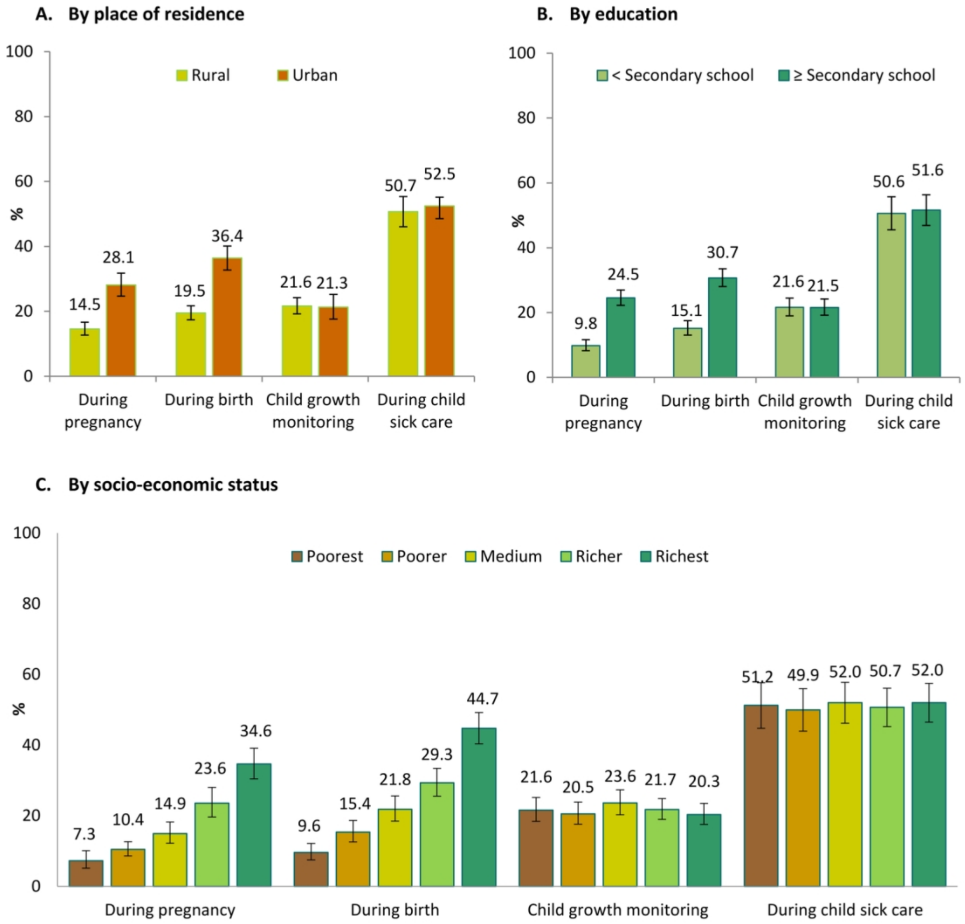

Figure 4

72x68mm (600 x 600 DPI)

Supplemental Table 1. Harmonized facility categories and reported categories in SPA and DHS

Harmonized facility category	SPA facility category	DHS facility category
District and upazila public facilities	District hospital (DH)<br>Upazila health complex (UHC)<br>Maternal and child welfare center (MCWC)	District hospital (DH)<br>Upazila health complex (UHC)<br>Maternal and child welfare center (MCWC)
Union level public facilities	Union health and family welfare center (UHFWC)/(UHFWC upgraded)<br>Union subcenter/rural dispensary	Upazila health & family welfare center<br>Satellite clinic/epi outreach
Public community clinic	Public community clinic	Public community clinic<br>Other public sectors
NGO clinic/hospital	NGO clinic/hospital	NGO static clinic<br>NGO satellite clinic<br>Other NGO clinic
Private clinic/hospital	Private clinic/hospital	Private clinic/hospital<br>Private medical<br>Traditional/Qualified doctor

For peer review only

Supplemental Table 2: Percentage of health facilities with structural input items, by division

		Barisal	Chittagong	Dhaka	Khulna	Rajshahi	Rangpur	Sylhet
**Antenatal care**								
**Routine availability of nutrition interventions[1]**	Iron/ Acid folic supplementation	67.79	76.89	88.41	72.75	85.68	78.23	93.25
	Intermittent preventive treatment for malaria (IPT)	3.49	6.86	6.78	0.64	5.51	4.42	3.39
	Weighing of clients	56.52	50.54	43.05	51.27	51.65	52.14	58.28
	Taking blood pressure	57.73	47.82	47.39	53.24	51.94	47.16	56.5
	Urine test for protein	3.97	3.57	8.69	6.06	10.76	17.19	9.78
	Blood test for anemia	4.95	3.08	6.94	1.87	5.88	16.96	6.55
	Ultrasonography	0.45	0.88	5.04	1.29	0.74	0.54	0.70
	Conducting group health education sessions	33.90	31.37	30.7	32.61	34.47	42.42	40.18
**Staff**	Staff with any training on ANC	38.55	57.24	57.8	44.27	35.87	39.45	54.35
**Guideline**	National guidelines for ANC	56.26	38.49	39.93	58.47	46.86	70.65	60.09
	Visual aids for client education	69.80	63.78	57.09	72.27	67.74	72.19	91.51
**Functional equipment**	Adult weighing scale	88.74	89.73	72.93	91.45	87.21	93.23	90.38
	Tape measure for fundal height	41.18	27.51	33.42	40.77	32.44	50.37	36.35
	Blood pressure apparatus	87.03	86.88	90.67	94.51	92.03	97.85	88.96
	Stethoscope	89.75	86.32	92.10	96.73	93.69	96.06	87.98
	Fetal stethoscope	13.36	7.84	15.89	13.24	8.39	29.11	26.26
**Diagnostics capacity**	Hemoglobin	5.91	8.68	12.46	3.71	9.29	19.29	11.60
	Urine protein	8.28	15.84	20.06	11.35	18.58	26.71	18.58
**Medicine**	Iron or folic acid tablets	96.99	91.40	91.52	90.51	96.58	100.00	94.70
**Birth care**								
**Routine availability of nutrition interventions**	Kangaroo practice	35.64	41.26	48.20	48.44	37.55	47.06	39.57
	Skin to skin contacts	52.92	60.54	71.35	85.17	63.57	90.55	62.84
	Initiation of breastfeeding within the first hour	91.98	97.71	100.00	98.81	100.00	91.05	87.99
	Do not give the newborn prelacteal liquids	91.37	82.08	68.68	93.28	78.34	76.31	77.25
	Weigh the newborn immediately	65.43	64.54	67.81	67.99	84.63	82.27	55.60
**Staff**	Staff with any training on IMPACT	23.68	33.01	45.01	37.02	35.94	43.94	34.23
**Guideline**	Guidelines for BEmOC or CEmOC	32.66	26.00	25.83	21.19	10.84	56.25	20.46
**Functional equipment**	Infant scale	52.46	56.88	49.54	61.50	77.21	89.71	52.01
	Manual or digital BP apparatus	90.45	89.58	94.13	82.86	100.00	100.00	92.91
**Child growth monitoring**								
**Routine availability of nutrition interventions**	Weighing the child	39.71	40.74	34.46	40.04	43.46	47.86	42.85
	Plotting child's weight on graph	24.45	27.65	21.23	23.10	38.82	34.38	18.48
**Staff**	Staff with any training on growth monitoring	50.08	46.13	55.37	47.18	50.20	40.08	49.72
**Guideline**	Guidelines for growth monitoring	57.50	48.33	37.09	51.80	41.36	65.11	22.03
**Functional equipment**	Child scale (child or infant)	69.87	67.66	50.28	60.79	75.85	96.16	82.95
	Length or height board	70.34	49.76	50.48	56.34	66.41	69.23	72.63
	Tape for measuring head	32.28	24.25	23.24	30.86	27.20	43.00	50.57
	Growth chart	83.21	69.66	45.23	71.65	60.51	91.50	66.73
Readiness score for child growth monitoring								

For peer review only

Sick child care								
**Routine availability of nutrition interventions**	Diagnose and/or treat child malnutrition	97.01	94.28	84.28	97.59	99.69	94.59	98.87
	Provide vitamin A supplementation to children	72.65	69.00	72.35	78.79	75.49	86.22	64.50
	Provide iron supplementation to children	37.19	56.07	67.36	60.26	73.79	80.93	57.49
	Provide zinc supplementation to children	80.52	59.31	79.16	69.97	73.71	88.11	80.68
	Provide deworming to children	96.15	98.79	96.78	96.65	97.95	97.04	99.34
	Providing group health education	32.00	33.08	35.90	36.20	36.42	39.01	31.05
	Weighing the child	39.71	40.74	34.46	40.04	43.46	47.86	42.85
	Plotting child’s weight on graph	24.45	27.65	21.23	23.10	38.82	34.38	18.48
**Staff**	Staff with any training on IMCI	67.19	48.62	57.54	42.75	52.18	55.86	58.89
**Guideline**	Guidelines for IMCI	66.62	42.83	38.33	51.17	45.03	77.60	58.55
	IMCI chart booklet	64.33	40.94	49.47	34.79	55.25	84.87	57.20
	IMCI mother's cards (IMCI card)	43.90	32.20	31.08	25.35	32.13	83.08	38.93
	Other visual aids for teaching caretakers	64.14	46.00	43.75	53.46	60.44	80.14	71.73
**Functional equipment**	Child scale (child or infant)	66.12	57.20	40.63	64.15	64.93	84.61	79.74
**Diagnostics capacity**	Hemoglobin	5.15	8.29	11.85	3.45	9.50	19.83	11.63
**Medicine**	ORS	71.97	63.52	61.01	51.95	74.91	69.94	52.35
	Albendazole/ Mebendazole	91.78	87.03	89.88	85.69	89.78	96.99	97.72
	Iron tablet	35.08	43.04	57.41	60.68	72.61	58.15	33.61
	Vitamin A	67.99	54.26	67.73	62.17	76.47	81.77	47.22
	Zinc tablet/ Zinc sulphate syrup	76.46	56.77	69.70	61.62	71.29	76.64	62.32

[1]Survey team assessing whether they see these practices in general in the facility at the time of the visit.
ANC: antenatal care, BEmOC: Basic Emergency Obstetric and Neonatal Care, CEmOC: Comprehensive Emergency Obstetric and Neonatal Care, IMCI: integrated management of childhood illness, IMPAC: integrated management of pregnancy and childbirth, ORS: Oral rehydration salts.

For peer review only

Supplemental Table 3: Percentage of health facilities with structural input items, by facility type

		DUPF	ULPF	PC	NGO	Private
**Antenatal care**						
**Routine availability of nutrition interventions[1]**	Iron/ Acid folic supplementation	84.21	79.68	82.82	78.76	56.46
	Intermittent preventive treatment for malaria (IPT)	21.74	5.34	2.97	10.97	30.99
	Weighing of clients	65.25	48.95	48.96	58.51	39.94
	Taking blood pressure	68.39	53.44	47.38	59.04	44.53
	Urine test for protein	32.42	3.89	5.80	34.68	35.79
	Blood test for anemia	33.24	4.08	3.06	29.73	35.79
	Ultrasonography	11.56	0.43	0.00	10.58	41.93
	Conducting group health education sessions	42.19	36.42	32.62	42.86	10.54
**Staff**	Staff with any training on ANC	83.64	50.04	46.31	60.36	27.48
**Guideline**	National or other guidelines for ANC	70.33	47.82	48.16	72.95	24.70
	Visual aids for client education	79.25	68.52	65.47	78.34	45.94
**Equipment**	Adult weighing scale	91.47	82.20	85.67	94.15	83.82
	Tape measure for fundal height	60.76	35.40	32.67	63.31	52.64
	Blood pressure apparatus	99.12	90.74	90.30	99.14	94.67
	Stethoscope	98.94	91.02	91.18	99.15	97.97
	Fetal stethoscope	46.35	20.38	8.67	46.55	43.29
**Diagnostics capacity**	Hemoglobin	61.97	7.90	3.53	58.87	72.14
	Urine protein	63.89	12.02	12.34	68.63	68.12
**Medicine**	Iron or folic acid tablets	96.41	94.51	94.57	89.92	66.29
**Birth care**						
**Routine availability of nutrition interventions[1]**	Kangaroo practice	61.43	33.04	40.84	57.97	51.62
	Skin to skin contacts	87.27	70.94	54.47	73.82	75.7
	Initiation of breastfeeding within the first hour	97.64	94.27	100.00	100.00	97.34
	Do not give the newborn prelacteal liquids	71.24	88.78	72.36	73.56	65.14
	Weigh the newborn immediately	85.97	61.98	55.57	92.25	88.85
**Staff**	Staff with any training on IMPACT	55.96	31.75	40.71	52.84	22.05
**Guideline**	Guidelines for BEmOC or CEmOC	40.50	26.09	27.42	27.94	11.29
**Equipment**	Infant scale	69.52	60.47	47.87	76.72	64.97
	Manual or digital BP apparatus	96.91	88.33	93.18	99.34	100.00
**Child growth monitoring**						
**Routine availability of nutrition interventions[1]**	Weighing the child	66.02	39.98	38.21	59.13	38.95
	Plotting child's weight on graph	40.61	24.96	26.54	43.87	18.55
**Staff**	Staff with any training on growth monitoring	67.88	42.91	50.18	47.73	40.35
**Guideline**	Guidelines for growth monitoring	52.55	43.30	46.22	64.57	29.31
**Equipment**	Child scale (child or infant)	82.28	74.50	64.19	86.20	90.63
	Tape for measuring head	47.65	26.41	28.95	57.15	32.90
	Length or height board	71.77	52.26	60.09	71.51	57.32
	Growth chart	85.51	68.72	63.01	83.71	85.13
Readiness score for child growth monitoring						
**Sick child care**						
**Routine availability of nutrition interventions[1]**	Diagnose and/or treat child malnutrition	98.00	93.50	93.19	90.08	97.29
	Provide vitamin A supplementation to children	79.18	43.62	85.92	75.11	58.28
	Provide iron supplementation to children	72.69	65.67	62.35	72.55	65.12
	Provide zinc supplementation to children	79.93	43.65	86.10	80.36	71.78
	Provide deworming to children	98.28	94.35	99.02	96.54	79.25
	Providing group health education	40.29	37.11	35.2	37.89	6.09

For peer review only - http://bmjopen.bmj.com/site/about/guidelines.xhtml

	Weighing the child	66.02	39.98	38.21	59.13	38.95
	Plotting child's weight on graph	40.61	24.96	26.54	43.87	18.55
**Staff**	Staff with any training on IMCI	78.46	52.70	54.11	45.91	32.97
**Guideline**	Guidelines for IMCI	76.74	55.46	47.93	56.32	30.23
	IMCI chart booklet	78.06	59.78	49.35	64.58	35.14
	IMCI mother's cards (IMCI card)	62.65	50.15	33.78	52.56	24.58
	Other visual aids for teaching caretakers	65.70	54.39	55.88	68.49	41.49
**Equipment**	Child scale (child or infant)	84.76	66.10	55.77	80.08	77.59
**Diagnostics capacity**	Hemoglobin	62.53	8.18	3.78	58.33	68.26
**Medicine**	ORS	64.45	34.50	73.57	78.80	46.65
	Albendazole/ Mebendazole	75.39	87.03	93.31	83.53	79.06
	Iron tablet	64.20	53.69	52.45	73.53	59.43
	Vitamin A	58.72	29.90	80.73	70.19	53.41
	Zinc tablet/ Zinc sulphate syrup	65.12	34.36	79.26	73.28	80.94

[1]Survey team assessing whether they see these practices in general in the facility at the time of the visit.
DUPF: District and upazila public facilities, ULPF: Union level public facilities Public community clinic
ANC: antenatal care, BEmOC: Basic Emergency Obstetric and Neonatal Care, CEmOC: Comprehensive Emergency Obstetric and Neonatal Care, IMCI: integrated management of childhood illness, IMPAC: integrated management of pregnancy and childbirth, ORS: Oral rehydration salts.

For peer review only

Supplemental Figure 1: Place of ANC and child care by facility type, Bangladesh 2014

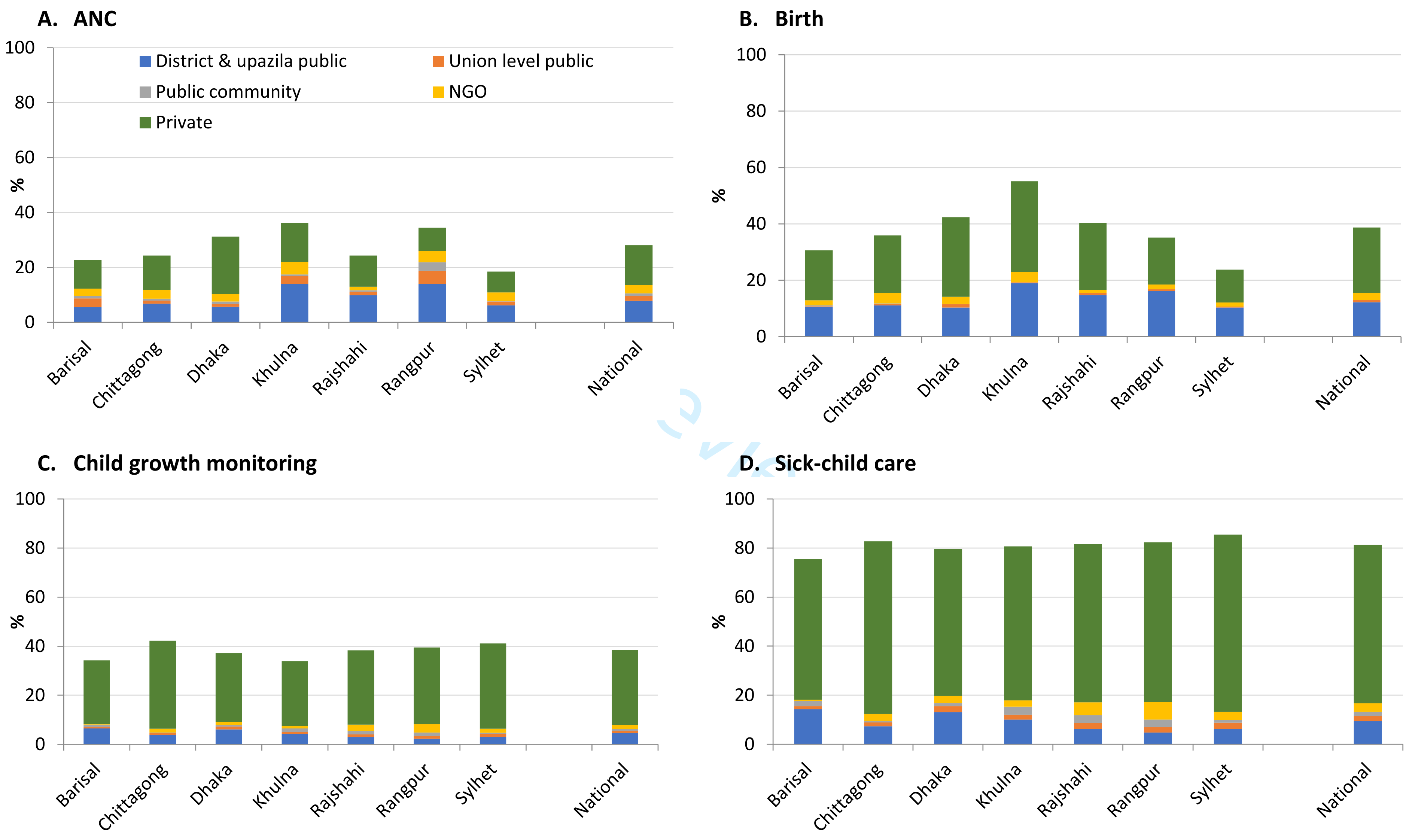

Source: DHS data

Supplemental Figure 2: Distribution of facilities with ANC and sick child care by facility type, Bangladesh 2014

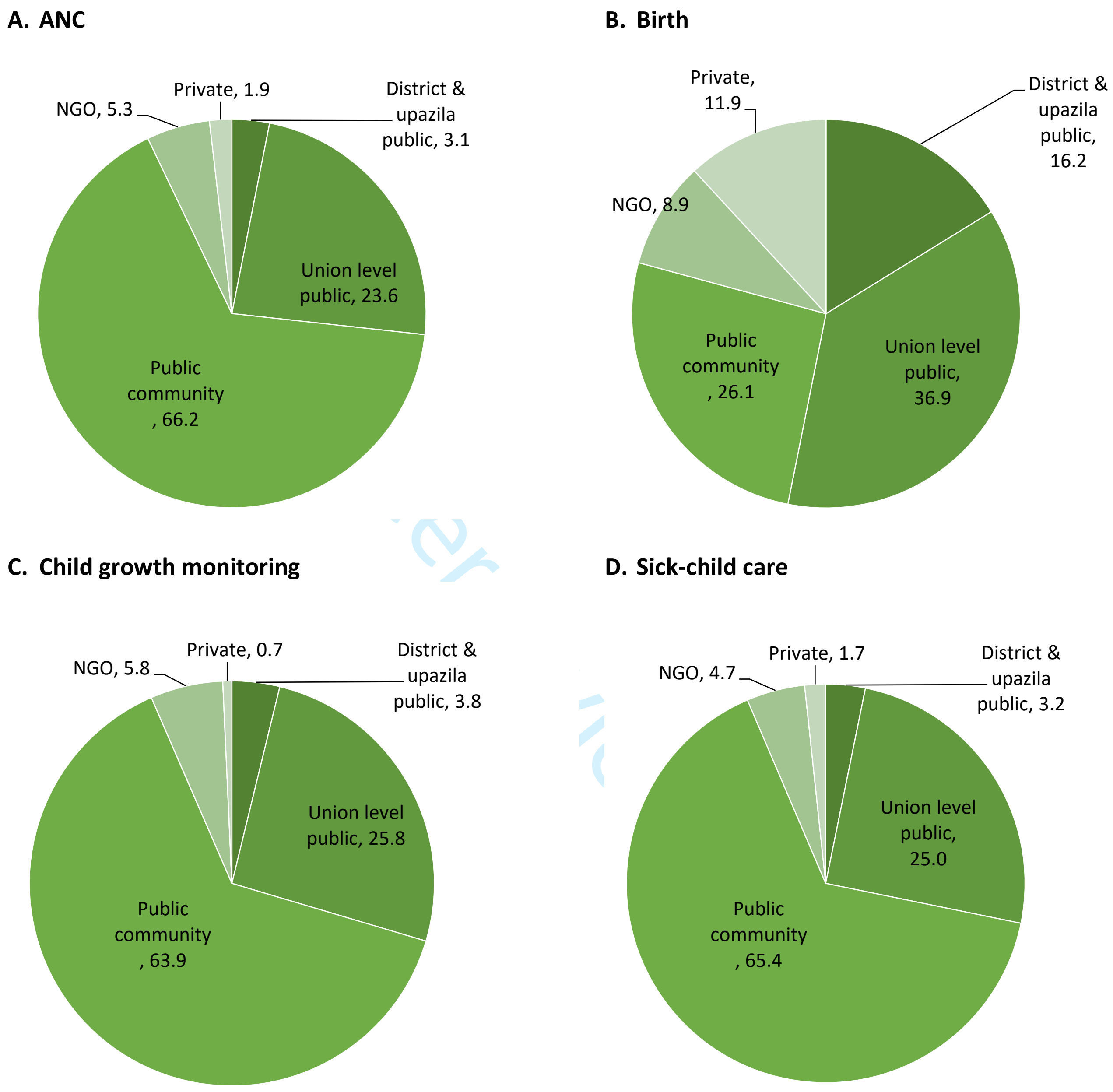

Source: SPA data

For peer review only - http://bmjopen.bmj.com/site/about/guidelines.xhtml

STROBE Statement—Checklist of items that should be included in reports of cross-sectional studies

	Item No	Recommendation	Page number from manuscript
**Title and abstract**	1	(*a*) Indicate the study's design with a commonly used term in the title or the abstract	1, 3
		(*b*) Provide in the abstract an informative and balanced summary of what was done and what was found	3-4
**Introduction**			
Background/rationale	2	Explain the scientific background and rationale for the investigation being reported	6-8
Objectives	3	State specific objectives, including any prespecified hypotheses	8
**Methods**			
Study design	4	Present key elements of study design early in the paper	9-10
Setting	5	Describe the setting, locations, and relevant dates, including periods of recruitment, exposure, follow-up, and data collection	9-10
Participants	6	(*a*) Give the eligibility criteria, and the sources and methods of selection of participants	9-10
Variables	7	Clearly define all outcomes, exposures, predictors, potential confounders, and effect modifiers. Give diagnostic criteria, if applicable	10-12
Data sources/ measurement	8*	For each variable of interest, give sources of data and details of methods of assessment (measurement). Describe comparability of assessment methods if there is more than one group	10-12
Bias	9	Describe any efforts to address potential sources of bias	NA
Study size	10	Explain how the study size was arrived at	NA
Quantitative variables	11	Explain how quantitative variables were handled in the analyses. If applicable, describe which groupings were chosen and why	13-15
Statistical methods	12	(*a*) Describe all statistical methods, including those used to control for confounding	13-15
		(*b*) Describe any methods used to examine subgroups and interactions	13-15
		(*c*) Explain how missing data were addressed	NA
		(*d*) If applicable, describe analytical methods taking account of sampling strategy	13-15
		(*e*) Describe any sensitivity analyses	NA
**Results**			
Participants	13*	(a) Report numbers of individuals at each stage of study—eg numbers potentially eligible, examined for eligibility, confirmed eligible, included in the study, completing follow-up, and analysed	15
		(b) Give reasons for non-participation at each stage	NA
		(c) Consider use of a flow diagram	NA
Descriptive data	14*	(a) Give characteristics of study participants (eg demographic, clinical, social) and information on exposures and potential	15

For peer review only - http://bmjopen.bmj.com/site/about/guidelines.xhtml

For peer review only

		confounders	
		(b) Indicate number of participants with missing data for each variable of interest	NA
Outcome data	15*	Report numbers of outcome events or summary measures	15-20
Main results	16	(*a*) Give unadjusted estimates and, if applicable, confounder-adjusted estimates and their precision (eg, 95% confidence interval). Make clear which confounders were adjusted for and why they were included	15-20
		(*b*) Report category boundaries when continuous variables were categorized	NA
		(*c*) If relevant, consider translating estimates of relative risk into absolute risk for a meaningful time period	NA
Other analyses	17	Report other analyses done—eg analyses of subgroups and interactions, and sensitivity analyses	NA
**Discussion**			
Key results	18	Summarise key results with reference to study objectives	21
Limitations	19	Discuss limitations of the study, taking into account sources of potential bias or imprecision. Discuss both direction and magnitude of any potential bias	26-27
Interpretation	20	Give a cautious overall interpretation of results considering objectives, limitations, multiplicity of analyses, results from similar studies, and other relevant evidence	21-24
Generalisability	21	Discuss the generalisability (external validity) of the study results	25
**Other information**			
Funding	22	Give the source of funding and the role of the funders for the present study and, if applicable, for the original study on which the present article is based	29

*Give information separately for exposed and unexposed groups.

Note: An Explanation and Elaboration article discusses each checklist item and gives methodological background and published examples of transparent reporting. The STROBE checklist is best used in conjunction with this article (freely available on the Web sites of PLoS Medicine at http://www.plosmedicine.org/, Annals of Internal Medicine at http://www.annals.org/, and Epidemiology at http://www.epidem.com/). Information on the STROBE Initiative is available at www.strobe-statement.org.

For peer review only

For peer review only - http://bmjopen.bmj.com/site/about/guidelines.xhtml

BMJ Open

Effective coverage of nutrition interventions across the continuum of care in Bangladesh: Insights from nationwide cross-sectional household and health facility surveys

Journal:	*BMJ Open*
Manuscript ID	bmjopen-2020-040109.R2
Article Type:	Original research
Date Submitted by the Author:	28-Oct-2020
Complete List of Authors:	Nguyen, Phuong Hong; International Food Policy Research Institute<br>Khương , Quỳnh Long ; Hanoi University of Public Health<br>Pramanik, Priyanjana ; International Food Policy Research Institute<br>Billah, Sk; ICDDRB, Maternal and Child Health Division<br>Menon, Purnima; International Food Policy Research Institute<br>Piwoz, Ellen; Bill and Melinda Gates Foundation,<br>Leslie, Hannah; Harvard TH Chan School of Public Health, Global Health and Population
**Primary Subject Heading**:	Health services research
Secondary Subject Heading:	Global health, Health services research, Public health
Keywords:	EPIDEMIOLOGY, International health services < HEALTH SERVICES ADMINISTRATION & MANAGEMENT, Quality in health care < HEALTH SERVICES ADMINISTRATION & MANAGEMENT, Public health < INFECTIOUS DISEASES, NUTRITION & DIETETICS

SCHOLARONE™
Manuscripts

I, the Submitting Author has the right to grant and does grant on behalf of all authors of the Work (as defined in the below author licence), an exclusive licence and/or a non-exclusive licence for contributions from authors who are: i) UK Crown employees; ii) where BMJ has agreed a CC-BY licence shall apply, and/or iii) in accordance with the terms applicable for US Federal Government officers or employees acting as part of their official duties; on a worldwide, perpetual, irrevocable, royalty-free basis to BMJ Publishing Group Ltd ("BMJ") its licensees and where the relevant Journal is co-owned by BMJ to the co-owners of the Journal, to publish the Work in this journal and any other BMJ products and to exploit all rights, as set out in our licence.

The Submitting Author accepts and understands that any supply made under these terms is made by BMJ to the Submitting Author unless you are acting as an employee on behalf of your employer or a postgraduate student of an affiliated institution which is paying any applicable article publishing charge ("APC") for Open Access articles. Where the Submitting Author wishes to make the Work available on an Open Access basis (and intends to pay the relevant APC), the terms of reuse of such Open Access shall be governed by a Creative Commons licence – details of these licences and which Creative Commons licence will apply to this Work are set out in our licence referred to above.

Other than as permitted in any relevant BMJ Author's Self Archiving Policies, I confirm this Work has not been accepted for publication elsewhere, is not being considered for publication elsewhere and does not duplicate material already published. I confirm all authors consent to publication of this Work and authorise the granting of this licence.

For peer review only

Effective coverage of nutrition interventions across the continuum of care in Bangladesh: Insights from nationwide cross-sectional household and health facility surveys

Authors:

Phuong Hong Nguyen[1*], Long Quynh Khuong[2], Priyanjana Pramanik[1], Sk Masum Billah[3 4], Purnima Menon[1], Ellen Piwoz[5], and Hannah H Leslie[6]

Author affiliations:

[1]International Food Policy Research Institute, Washington, DC, USA; [2]Hanoi University of Public Health, Hanoi, Viet Nam; [3]Maternal and Child Health Division, ICDDR, B, Dhaka, Bangladesh; [4]The University of Sydney, Faculty of Medicine and Health, Sydney School of Public Health, New South Wales, Australia [5]Bill & Melinda Gates Foundation; [6]Harvard T.H. Chan School of Public Health, Boston, MA, USA

***Corresponding author:**

Phuong Hong Nguyen, Poverty, Health and Nutrition Division, International Food Policy Research Institute, 2005 I Street, NW, Washington DC, 20005. Phone: 202-826-4088. Email: P.H.Nguyen@cgiar.org

Word count: 4,642 (from introduction to conclusion, not including tables)

Abbreviations

ANC	: Antenatal care
BEmONC	: Basic Emergency Obstetric and Neonatal Care
CEmONC	: Comprehensive Emergency Obstetric and Neonatal Care
DHS	: Demographic and Health Surveys
DUPF	: District and upazila public facilities

For peer review only

For peer review only - http://bmjopen.bmj.com/site/about/guidelines.xhtml

23	IFA	: Iron folic acid
24	IMCI	: Integrated management of childhood illness
25	IMPAC	: Integrated management of pregnancy and childbirth
26	LMIC	: Low- and middle-income countries
27	MUAC	: Mid-upper arm circumference
28	NGO	: Non-governmental organization
29	ORS	: Oral rehydration salts
30	PC	: Public community clinic
31	Pp	: Percentage points
32	SES	: Socio-economic status
33	SPA	: Service Provision Assessment
34	ULPF	: Union level public facilities
35	WHO	: World Health Organization

For peer review only

For peer review only - http://bmjopen.bmj.com/site/about/guidelines.xhtml

ABSTRACT

Introduction: Improving the impact of nutrition interventions requires adequate measurement of both reach and quality of interventions, but limited evidence exists on advancing coverage measurement. We adjusted contact-based coverage estimates, taking into consideration the inputs required to deliver quality nutrition services, to calculate input-adjusted coverage of nutrition interventions across the continuum of care from pregnancy through early childhood in Bangladesh.

Methods: We used data from the 2014 Bangladesh Demographic and Health Surveys to assess use of maternal and child health services and the 2014 Service Provision Assessment to determine facility readiness to deliver nutrition interventions. Service readiness captured availability of nutrition-specific inputs (including human resources and training, equipment, diagnostics, and medicines). Contact coverage was combined with service readiness to create a measure of input-adjusted coverage at the national and regional levels, across place of residence, and by maternal education and household socio-economic quintiles.

Results: Contact coverage varied from 28% for attending at least 4 ANC visits to 38% for institutional delivery, 35% for child growth monitoring and 81% for sick-child care. Facilities demonstrated incomplete readiness for nutrition interventions, ranging from 48 to 51% across services. Nutrition input-adjusted coverage was suboptimal (18% for ANC, 23% for institutional delivery, 20% for child growth monitoring and 52% for sick-child care) and varied between regions within the country. Inequalities in input-adjusted coverage were large during ANC and institutional delivery (14-17 percentage points [pp] between urban and rural areas, 15pp

For peer review only - http://bmjopen.bmj.com/site/about/guidelines.xhtml

between low and high education, and 28-34pp between highest and lowest wealth quintiles), and less variable for sick-child care (< 2pp).

Conclusion: Nutrition input-adjusted coverage was suboptimal and varied sub-nationally and across the continuum of care in Bangladesh. Special efforts are needed to improve the reach as well as the quality of health and nutrition services to achieve the Sustainable Development Goals.

Keywords: Bangladesh, facility readiness, input-adjusted coverage, nutrition, effective coverage

For peer review only

For peer review only - http://bmjopen.bmj.com/site/about/guidelines.xhtml

Strengths and limitations of this study

- Our study used nationally representative population and health system data to estimate input-adjusted coverage for nutrition interventions across the continuum of maternal and early childhood care.
- Our study quantified inequities in input-adjusted coverage within the population and indicated potential priorities for improvement, including special investment in reaching underserved populations with key services, while also ensuring adequate quality of services.
- Although the SPA survey was quite comprehensive, it did not capture every aspect to measure facility readiness to provide nutrition interventions or to calculate each step of care cascades from need for services through to health benefits.
- The exclusion of small private clinics hospitals with less than 20 beds could lead to an overestimation of quality for private sector users, as larger facilities tend to have higher readiness than smaller ones.

For peer review only - http://bmjopen.bmj.com/site/about/guidelines.xhtml

INTRODUCTION

Global evidence suggests that if high-quality health systems could effectively deliver a core subset of 19 maternal and child interventions, almost one quarter of the maternal deaths, neonatal deaths, and stillbirths would be prevented.[1] Access to ten evidence-based nutrition interventions alone could reduce the mortality rate in children under 5 by 15%.[2] Despite this, coverage data for high-impact interventions along the continuum of care remain limited due to challenges in data collection and measurement.[3] Improving the impact of nutrition interventions requires adequate measurement of both reach and quality of interventions. Current global measurement mainly focuses on contact coverage indicators,[4] but does not adequately capture the quality of services delivered, thus providing only weak links with actual health benefits received by the population in need.[5]

Effective coverage has been variously defined as 'the fraction of maximum possible health gain an individual with a health care need can expect to receive from the health system'[6] and as a situation where 'people who need health services obtain them in a timely manner and at a level of quality necessary to obtain the desired effect and potential health gains.[7] Effective coverage goes beyond contact coverage measurement by using not just the receipt of services by clients but also the quality of care received. Multiple quality domains can be considered, including inputs (service and equipment availability, training etc.), service delivery processes (e.g. compliance with protocols and standards of care), and outcomes (health benefits, client satisfaction etc.).[8] However, different studies have used various definitions and measurements for effective coverage, thus limiting the comparability among studies. To address this challenge, the recently proposed seven-step coverage framework begins with the target client population,

For peer review only - http://bmjopen.bmj.com/site/about/guidelines.xhtml

and following them through a hypothetical cascade of the losses of health benefits at different stages, including service contact (an individual in need contacts health service), input-adjusted coverage (contacts health service that is ready), intervention coverage (receives health services), quality-adjusted coverage (receives health service according to standard), user adherence -adjusted coverage (user adherence) and outcome-adjusted coverage (health gain achieved).[5 9]

Effective coverage has been found to be considerably lower than contact coverage for antenatal care (ANC),[10 11] family planning, and sick-child care in several low and middle income countries (LMICs).[11] Findings from LMICs also suggested low levels of availability, readiness, and coverage of obstetric services[12] and showed substantial reductions (20-39 percentage points [pp]) from contact coverage to effective coverage in facility birth.[13] Overall, a review of 36 studies showed large gaps between contact coverage and quality-adjusted coverage levels (10 to 38 pp) across the continuum of care for reproductive, maternal, newborn, and child health.[5]

Although recent studies have paid more attention to capturing the quality of care and quantifying the gaps between contact coverage and effective coverage across the continuum of care, studies on nutrition-related interventions are scarce. Previous studies mainly came from small-scale studies that focused on fortified foods where contact coverage was measured through exposure to a particular fortified food and quality-adjusted measures were based on regular consumption of the fortified food.[5] Recently, one study in Malawi showed that even though the utilization of ANC and facility delivery was high, women often did not receive nutrition-related interventions (such as iron interventions, nutrition counseling and breastfeeding counseling).[14]

For peer review only - http://bmjopen.bmj.com/site/about/guidelines.xhtml

Bangladesh has observed significant improvement in contact coverage in maternal and child health in the last decade,[15] yet substantial gaps remain in coverage of nutrition services, with less than 30% of women receiving all four key ANC nutrition interventions - IFA supplement, calcium supplements, dietary advice and weight monitoring, and only 25% of sick children having their weight checked against a growth-chart.[16] Large gaps in facility readiness to provide nutrition interventions persist. Specifically, only around half of providers reported having received any training on nutrition, and only 13% of facilities possessed more than 5 of the 13 items deemed essential for service delivery for children.[16] Previous studies in Bangladesh have reported separately on contact coverage[17-19] and quality of services provided.[16 20] This paper aimed to adjust contact-based health coverage estimates in Bangladesh, taking into consideration the inputs required to deliver quality nutrition interventions across the continuum of care, specifically ANC and delivery for women and growth monitoring and curative care for young children.

DATA AND METHODS

Data sources

We used two sets of publicly-available nationally representative data: the 2014 Bangladesh Demographic and Health Survey (DHS)[21] and the 2014 Service Provision Assessment (SPA).[22] The DHS survey was a population-based household surveys that provided representative data on health and nutrition indicators at national and regional levels.[21] The survey was based on a two-stage stratified sample of households. In the first stage, 600 enumeration areas were selected with probability proportional to the enumeration area size. In

the second stage of sampling, a systematic sample of 30 households on average was selected from a household list in each selected cluster by equal probability sampling. All women in selected households with a birth in the three years before the survey were interviewed about antenatal care and birth care for the most recent live birth. Child curative care questions were asked for all living child under 5 years in the household.

The SPA is a formal health facility-based survey designed to provide information on health service availability and readiness, with an emphasis on the delivery of reproductive, maternal, newborn and child health services.[22] The Bangladesh SPA included a standard set of survey instruments for a facility inventory and a health care provider interview. Facilities were assessed for the availability of health services, basic amenities, equipment, laboratory services, essential medicines, and human resources. The SPA survey captures tracer indicators defined jointly by USAID and WHO to monitor health system strengthening [23]. Health facilities were sampled from a complete listing of 19,184 registered health facilities in the country, with the aim to provide the survey results separately for the seven administrative divisions and different types of public facilities, NGO clinics/hospitals, and private hospitals. Small private clinics with less than 20 beds were excluded. Further details on the survey's sampling strategy were presented in the survey report.[22] The Bangladesh SPA did not include observation of selected client visits or exit interviews.

Statistical analysis

Input-adjusted coverage for nutrition intervention was calculated among individuals in need of care as the product of the coverage of services and the readiness of health facilities to provide quality care.

For peer review only - http://bmjopen.bmj.com/site/about/guidelines.xhtml

Coverage of services

We estimated coverage of services based on DHS data, accounting for the DHS sampling weights. Coverage of ANC care was defined as the percentage of women 15–49 years old with at least one live birth in the three years preceding the survey who reported at least 4 ANC visits (one of those visits from a trained provider) for their most recent birth. Coverage of institutional delivery was defined as the percentage of women with a live birth in the 3 years preceding the survey who gave birth in a health facility. There was no consensus on coverage measurement for child growth monitoring. However, growth monitoring activities, such as height, weight and mid-upper arm circumference (MUAC) measurement, are intended to be a core part of the Integrated Management of Childhood Illness (IMCI) protocol that is followed during sick-child contacts[16] which has been shown to have an impact on childhood stunting.[24] Based on this, and given the lack of survey questions that explicitly ask respondents about growth monitoring services, coverage of growth monitoring was defined as the percentage of all children under 5 years of age who sought care for illness at a formal health facility. Care-seeking for well child visits is not captured in the DHS; moreover, delivery of growth monitoring services in Bangladesh is delivered via IMCI, so while this indicator provides an incomplete picture of growth monitoring, it does correspond to the current service delivery model. Coverage of sick-child care was defined as the percentage of children with any illness who sought care at a formal health facility.

Facility readiness to provide nutrition interventions

We analyzed facility readiness to deliver nutrition interventions based on five attributes as guided by the World Health Organization (WHO)’s Service Availability and Readiness

For peer review only - http://bmjopen.bmj.com/site/about/guidelines.xhtml

175 Assessment Manual[25] including: 1) trained personnel, 2) guidelines, 3) equipment, 4) diagnostic
176 capacity, and 5) medicines. We used the tracer indicators that are most relevant to capture
177 selected nutrition interventions. The selected indicators for estimating input-adjusted coverage
178 for nutrition interventions across continuum of care are presented in **Table 1**. During ANC,
179 multiple nutrition interventions should occur; these include delivering iron folic acid (IFA)
180 supplementation, weight monitoring for pregnant women, anemia management, blood
181 pressure management, fetal growth monitoring and health and nutrition education. During
182 birth, the main nutrition intervention is support for early breastfeeding practices. During early
183 childhood, national protocols are for children to be weighed and to receive nutrition education,
184 vitamin A supplementation, deworming, anemia management, and ORS and zinc for diarrhea.
185 We calculated the composite readiness score using a weighted additive approach, which
186 weights the 5 domains equally within each measure. Previous studies have found that three
187 methods (simple additive, weighted additive, or principal component analysis) for calculating
188 quality indices yield generally comparable results. [26] The weighted additive method has been
189 recommended[27] as easy to calculate and interpret. The domain scores were calculated by
190 averaging the indicators within each domain; domain scores were then summed and multiplied
191 by 100 to create the total weighted additive scores which expressed as percent of total readiness.
192 The calculated readiness score for a specific facility category was an average score of all
193 facilities in the same category. We accounted for the SPA sampling weight when summarizing
194 readiness scores.
195

For peer review only - http://bmjopen.bmj.com/site/about/guidelines.xhtml

Table 1: Definition of indicators for estimating input-adjusted coverage for nutrition interventions across continuum of care

Services	Associated nutrition interventions	Measure of need (denominator)	Measure of use (numerator)	Quality estimator
Antenatal care	• IFA supplementation<br>• Weight monitoring for pregnant women<br>• Anemia management<br>• Blood pressure management<br>• Fetal growth monitoring<br>• Health and nutrition education	Women 15–49 years old with at least one child under 3 years	Women 15–49 years old with at least one child under 3 years, whom for their most recent birth, reported at least 4 ANC visits (ANC4+)	Human resources<br>• Staff with any training on ANC<br>Guidelines<br>• National guidelines for ANC, visual aids for client education<br>Basic equipment<br>• Adult weighing scale<br>• Tape measure for fundal height<br>• Blood pressure apparatus<br>• Stethoscope<br>• Fetal stethoscope<br>Diagnostic capacity<br>• Hemoglobin,<br>• Urine protein<br>Essential medicines<br>• Iron tablets<br>• IFA tablets
Birth care	• Child birthweight measures<br>• Early breastfeeding practices	Women 15–49 years old with at least one child under 5 years	Women 15–49 years old with at least one child under 5 years, whom for their most recent birth, reported delivery in a health facility	Human resources<br>• Staff with any training on IMPACT<br>Guidelines<br>• Guidelines on basic birth care BEmONC<br>• Guidelines on comprehensive birth care: CEmONC<br>Basic equipment<br>• Infant scale<br>• Manual or digital BP apparatus
Child growth monitoring	Child growth monitoring	All children alive between 0 and 59 months	All children who had diarrhea or ARI symptoms for whom care was sought from a medical provider	Human resources<br>• Staff with any training on growth monitoring<br>Guidelines<br>• Guidelines for growth monitoring<br>Basic equipment:<br>• Scale<br>• Length or height board<br>• Tape for measuring head<br>• Growth chart
Sick child care	• Nutrition education<br>• Vitamin A supplementation<br>• Deworming<br>• Anemia management<br>ORS and zinc for diarrhea	All children alive between 0 and 59 months who had diarrhea or ARI in the last two weeks	All children who had diarrhea or ARI symptoms for whom care was sought from a medical provider	Human resources<br>• Staff with any training on IMCI<br>Guidelines<br>• IMCI guideline: national guidelines for IMCI, IMCI chart booklet, IMCI card, other visual aids<br>Basic equipment<br>• Scale

For peer review only

For peer review only - http://bmjopen.bmj.com/site/about/guidelines.xhtml

Services	Associated nutrition interventions	Measure of need (denominator)	Measure of use (numerator)	Quality estimator
				Diagnostic capacity<br>• Hemoglobin<br>Essential medicines:<br>• ORS<br>• Albendazole/ mebendazole<br>• Iron tablet<br>• Vitamin A<br>• Zinc tablet/ zinc sulphate syrup.

198

199 ***Estimating input-adjusted coverage***

200 We linked health facility and household survey data at the aggregate level, using an ecological
201 linking method which has been shown a feasible and valid approach for estimating quality-
202 adjusted effective coverage.[28] We disaggregated the coverage by type of facility where services
203 were sought. In case women went to more than one type of facility for ANC, we used the
204 highest level of facility for analyses. Facility types were harmonized between the DHS and SPA
205 at 5 levels: district and upazila public facilities, union level public facilities, public community
206 clinic, NGO clinic/hospital and private clinic/hospital (Supplemental Table 1).
207 Input-adjusted coverage was estimated at both the national and regional level (7 divisions),
208 accounting for 5 types of facilities where the care was sought. At the regional level, the input-
209 adjusted coverage was the summation of input-adjusted coverage of each type of facility that
210 was constructed as the product of the coverage and readiness estimates:

211 $$IAC_r = \sum_j C_{rj} * Q_{rj}$$

212 where IAC_r represented input-adjusted coverage in region r,
213 C_{rj} was the proportion of women/children who sought care in facility type j in region r,
214 And Q_{rj} was the average readiness score of facility type j in region r.

For peer review only - http://bmjopen.bmj.com/site/about/guidelines.xhtml

The national input-adjusted coverage was the summation of regional input-adjusted coverage weighted by the proportion of users in each region:

$$IAC_T = \sum_r IAC_r * w_r$$

where w_r was the proportion of users in region r.

The variance of input-adjusted coverage estimates was calculated with an approximation procedure referred to as the "delta" method which has been used and described in detail in previous research.[29] Finally, we calculated the input-adjusted coverage by strata of place of residence, educational level and socio-economic (SES) quintiles. This analysis assumed that there was no systematic difference in facility readiness within region by the education or socioeconomic stratifiers (e.g. that within a given sub-national region, the average readiness of public community clinics used by highly educated women was the same as average readiness of public community clinics used by less educated women). Data analysis was performed using Stata version 16.0.

Patient and public involvement

Patients and/or the public were not involved in the design, or conduct, or reporting, or dissemination plans of this research.

For peer review only - http://bmjopen.bmj.com/site/about/guidelines.xhtml

RESULTS

The household survey included 17,863 women (out of 18,245 eligible women aged 15-49 years, a response rate of 98%), among them 4,488 women reporting a birth in the three years preceding the survey and 7,886 women with living children under 5 years. Health service coverage varied considerably between services, with low coverage for ANC4+ (28%), institutional delivery (38%), child growth monitoring (35%), but substantially higher for sick-child care (81%). The place of ANC and childcare by facility type was shown in **Supplemental Figure 1.**

The health facility survey included 1,548 facilities of 1,596 sampled (97% response rate); among them 1,493 facilities (96%) offering services on antenatal care, 586 (38%) offering services on delivery care, and 1,463 (92%) offering curative care for sick children. Government health facilities are the major providers of care (**Supplemental Figure 2)**.

The availability of tracer indicators that would be required to deliver interventions across the continuum of care is presented in **Table 2** for the national level. Overall, facilities lacked trained personnel, guidelines, equipment, medicines or supplies needed to provide high quality of care (**Table 2**). Of facilities offering each service, approximately half of facilities had staff with any training on ANC, child growth monitoring, and IMCI, but a lower proportion of facilities (39%) had staff with any training on integrated management of pregnancy and childbirth (IMPAC). Similarly, around half of facilities had national or other guidelines for ANC, growth monitoring or IMCI, but only 27% had guidelines for Basic Emergency Obstetric and Neonatal Care (BEmONC) or Comprehensive Emergency Obstetric and Neonatal Care (CEmONC). While a majority of facilities (>85%) had adult weighing scales, blood pressure monitors and

For peer review only - http://bmjopen.bmj.com/site/about/guidelines.xhtml

253 stethoscopes, only 36% had tape measures for fundal height and 15% had fetal stethoscopes.
254 Child scales, length or height board and growth chart were only available in 60-70% of facilities
255 that offered sick-child care. Regarding diagnostic capacity, 11% of facilities were able to test for
256 hemoglobin and 18% for urine protein. Iron-folic acid supplements were available in 91% of
257 facilities. Zinc or vitamin A supplementation was reported in three quarters of facilities and
258 deworming for children was reported in almost all facilities. The availability of these items
259 varied by division (**Supplemental Table 2**) and by types of facilities (**Supplemental Table 3**).

260 **Table 2: Percentage of health facilities with structural input items and their readiness score**

	Input items	% facilities with item available	95% CI
**Antenatal care (n=1493 facilities)**			
**Staff**	Staff with any training on ANC	48.75	43.24-54.26
**Guideline**	National or other guidelines for ANC	49.64	41.88-57.40
	Visual aids for client education	66.93	60.71-73.15
**Functional equipment**	Adult weighting scale	85.44	81.83-89.06
	Tape measure for fundal height	36.17	29.37-42.97
	Blood pressure apparatus	91.22	88.21-94.24
	Stethoscope	91.93	89.06-94.79
	Fetal stethoscope	15.25	10.77-19.74
**Diagnostics capacity**	Hemoglobin	10.57	7.15-13.99
	Urine protein	17.87	13.62-22.12
**Medicine**	Iron tablets	60.06	51.75-68.37
	IFA tablets	83.88	79.02-88.73
Readiness score for ANC		***51.44***	***48.56-54.32***
**Birth care (n=586 facilities)**			
**Staff**	Staff with any training on IMPAC	38.74	30.01-47.47
**Guideline**	Guidelines for BEmONC or CEmONC	27.18	17.28-37.09
**Functional equipment**	Infant scale	60.64	52.62-68.65
	Manual or digital BP apparatus	93.35	89.08-97.62
Readiness score for birth care		***47.64***	***41.81-53.46***
**Child growth monitoring (n=1463 facilities)**			
**Staff**	Staff with any training on growth monitoring	48.76	42.46-55.07
**Guideline**	Guidelines for growth monitoring	46.65	39.28-54.01
**Functional equipment**	Child scale (child or infant)	69.00	63.08-74.92
	Length or height board	59.16	51.64-66.67
	Tape for measuring head	30.67	24.48-36.86
	Growth chart	66.70	60.66-72.73
Readiness score for child growth monitoring		***50.60***	***46.86-54.34***
**Sick child care (n=1463 facilities)**			
**Staff**	Staff with any training on IMCI	53.85	47.36-60.33
**Guideline**	Guidelines for IMCI	50.84	44.29-57.39

For peer review only - http://bmjopen.bmj.com/site/about/guidelines.xhtml

	Input items	% facilities with item available	95% CI
	IMCI chart booklet	53.35	47.13-59.56
	IMCI mother's cards (IMCI card)	39.48	33.50-45.45
	Other visual aids for teaching caretakers	56.23	50.36-62.09
**Functional equipment**	Child scale (child or infant)	60.80	54.16-67.44
**Diagnostics capacity**	Hemoglobin	10.34	6.85-13.84
**Medicine**	ORS	63.59	58.14-69.04
	Albendazole/mebendazole	87.59	83.68-91.50
	Iron tablet	54.35	46.15-62.56
	Vitamin A	66.52	60.60-72.44
	Zinc tablet/zinc sulphate syrup	65.75	59.92-71.57
Readiness score for sick child care		***48.30***	***44.90-51.69***

ANC: antenatal care, BEmONC: Basic Emergency Obstetric and Neonatal Care, CEmONC: Comprehensive Emergency Obstetric and Neonatal Care, IMCI: integrated management of childhood illness, IMPAC: integrated management of pregnancy and childbirth, ORS: Oral rehydration salts.

Readiness scores were calculated at the national (**Table 2**) and regional levels and by facility type (**Figure 1**). Overall, facilities demonstrated 51% of the expected resources to provide nutrition interventions during ANC, 48% for delivery care, 51% for growth monitoring, and approximately half the required resources for child health services. Readiness scores varied by regions and depending on the service. Rangpur performed the best overall, and Sylhet showed high readiness for ANC but lowest on delivery care and average on others (**Figure 1**). District and upazila public facilities had the highest readiness scores across the continuum of care, whereas lower-level facilities (union level and public community) were much less prepared. For example, district public hospitals had the highest readiness score, with 76% of the expected capacity to provide nutrition interventions during ANC care services, but public community clinics had a readiness score of only 49%.

<Insert Figure 1 here>

Figure 2 plots health service coverage against facilities' readiness score in each region of the country. For ANC4+, the regional averages of coverage and readiness fell mostly in quadrant

For peer review only - http://bmjopen.bmj.com/site/about/guidelines.xhtml

III for all regions, indicating both low coverage (ranged between 18-36%) and moderate readiness (between 48-57%) (Table 3), thus the priority is improving coverage as well as quality of nutrition interventions for ANC care. Similar priority was observed for delivery care: most regions had low access (30-40% except Khulna) and low readiness (less than 50% except Rangpur). For child growth monitoring, all regions showed low coverage (30-39%); Dhaka had the lowest readiness (45%) while other regions had a higher but wide range of readiness (50-60%). For sick-child care, regions had similar levels of high coverage (>75%) but showed lower and highly variable readiness.

<Insert Figure 2 here>

Taking into account the readiness of facilities to provide nutrition interventions and the use of services, we estimated the input-adjusted coverage at the national and regional level (**Table 3 and Figure 3**). At the national level, input-adjusted coverage was 18% for ANC4+, 23% for delivery care, 20% for child growth monitoring and 52% for sick-child care. The gaps between contact and input-adjusted coverage ranged between 10-30 percentage points (pp).

<Insert Figure 3 here>

For peer review only

For peer review only - http://bmjopen.bmj.com/site/about/guidelines.xhtml

Table 3: Estimated regional and national input-adjusted coverage of nutrition interventions across continuum of care, Bangladesh 2014

	Contact coverage	Readiness score	Input-adjusted coverage	
			Estimate	95% CI
**Antenatal care 4+**				
Barisal	22.79	48.04	14.72	11.02-19.38
Chittagong	24.35	49.09	15.80	12.85-19.28
Dhaka	31.19	50.90	19.96	16.59-23.82
Khulna	36.21	51.65	23.20	19.01-27.99
Rajshahi	24.36	50.24	16.06	12.49-20.42
Rangpur	34.44	57.05	23.35	18.33-29.24
Sylhet	18.44	55.13	12.25	8.74-16.92
**Overall**	**28.06**	**51.44**	**18.23**	**16.59-19.99**
**Birth care**				
Barisal	30.38	42.60	17.70	13.07-23.53
Chittagong	35.49	44.08	21.87	17.83-26.53
Dhaka	40.91	47.56	24.82	20.46-29.76
Khulna	54.96	43.47	34.96	26.76-44.17
Rajshahi	39.23	45.13	23.57	19.38-28.34
Rangpur	34.52	65.02	22.89	17.70-29.07
Sylhet	22.87	42.39	14.12	10.79-18.25
**Overall**	**37.73**	**47.64**	**23.24**	**21.11-25.51**
**Child growth monitoring**				
Barisal	32.77	57.17	21.79	17.33-27.01
Chittagong	36.78	49.10	22.57	19.45-26.03
Dhaka	33.87	44.92	20.47	17.84-23.37
Khulna	29.79	51.30	18.27	14.97-22.12
Rajshahi	35.34	49.68	8.94	7.66-10.41
Rangpur	36.99	60.06	24.93	17.99-33.45
Sylhet	38.51	46.66	23.64	16.39-32.84
**Overall**	**35.05**	**50.60**	**20.41**	**18.76-22.16**
**Sick child care**				
Barisal	76.83	53.28	50.95	41.64-60.19
Chittagong	82.92	42.65	51.31	41.50-61.02
Dhaka	80.95	43.29	50.50	42.42-58.56
Khulna	77.28	43.43	47.66	38.13-57.36
Rajshahi	78.53	50.04	51.92	42.02-61.68
Rangpur	82.69	63.54	58.02	48.95-66.59
Sylhet	84.38	52.60	52.69	40.21-64.84
**Overall**	**81.20**	**48.30**	**51.67**	**47.56-55.76**

When examining the inequality gaps, we found that input-adjusted coverage varied by as much as 6-32 pp between regions within the country (Figure 3). Inequality in input-adjusted coverage was large during pregnancy and delivery (14-17 pp between urban and rural areas, 15

For peer review only - http://bmjopen.bmj.com/site/about/guidelines.xhtml

pp between low and high education, and 28-34 pp between the rich and the poor), but narrower for child growth monitoring and sick-child care (<2 pp) (Figure 4).

<Insert Figure 4 here>

DISCUSSION

Improving both coverage and quality of healthcare services is essential to achieving universal health coverage and achieving the third Sustainable Development Goal of ensuring healthy lives and promoting well-being for all at all ages.[30] Our study is one of very few to develop and apply nutrition-related metrics of effective coverage across the continuum of care by incorporating service readiness of nutrition interventions into coverage estimates of ANC and post-delivery care. Our findings showed low levels of coverage across the continuum of care (28-38%), except for sick-child care (81%); we also found moderate levels of facility readiness to provide nutrition interventions (42-65). After adjustment for readiness, the input-adjusted coverage was found to be 10-30 pp lower than the contact coverage, and there was high level of inequality between regions within the country, residential areas, education and SES status for ANC and birth care, but less so for sick-child care. Because our estimates focused on inputs to care and did not capture how often these inputs were provided in adherence with evidence-based protocols, they should be considered an upper limit of the quality of nutrition care actually received.

The large gap between contact and adjusted coverage in our study was aligned with a review of 36 studies finding a range of 10-38 pp reductions between crude coverage and quality-adjusted coverage across the continuum of care.[5] These findings indicated that women

For peer review only - http://bmjopen.bmj.com/site/about/guidelines.xhtml

who sought care in a health facility did not necessarily receive the quality of care needed for them and their children. Our findings provided insight into health system challenges and indicate potential priorities for improvement.

For ANC, the priority is ensuring that all four contacts are provided by a trained provider, while improving coverage and quality simultaneously to reach high levels of effective coverage for nutrition interventions. We found a low contact coverage of 4+ ANC (28%), with evidence of inequities by wealth, education and place of residence. Evidence from a systematic review showed that both demand-side (such as maternal education, spousal education, marital status, women's employment, autonomy, cultural beliefs, media exposure, and household income) and supply-side factors (such as service availability and cost) influence ANC uptake.[31 32] Financial and transport constraints may be a greater barrier for women in remote and poorer areas.[33 34] Interventions such as mobile health technologies have been found to improve ANC attendance,[35] but improving coverage requires targeted investments to reach underserved communities. We also found low readiness of the health system to provide nutritional interventions during ANC (51%) which is consistent with a previous study in Bangladesh.[16] Our findings suggested that facility readiness can be improved by ensuring the availability of trained providers and necessary equipment, as well as strengthening diagnostic capacity.

For birth care, the priority clearly lies in improving coverage of facility-based deliveries while also improving readiness and provider compliance with labor management protocols to increase support for early initiation of breastfeeding. Although the trend of institutional delivery has increased over time from 29% in 2011[36 37] to 38% at the time of the survey in 2014, nearly two third of all births still take place at home. Women's educational interventions and

For peer review only - http://bmjopen.bmj.com/site/about/guidelines.xhtml

community-level mobilization may increase the coverage of institutional delivery.[38 39] In terms of quality, facilities have low readiness (48%) to provide nutrition interventions, particularly activities related to early initiation of breastfeeding, thus additional efforts are needed to improve the overall quality of service for facility-based delivery, including training staff, providing guidelines, emergency transport and necessary equipment.[40]

For care for children under 5, the priority is improving both coverage and quality of nutrition interventions for child growth monitoring. While community-based management of acute malnutrition activities such as screening and growth monitoring has been prioritized under the Bangladesh National Nutrition Services,[41] there is little data on the coverage and uptake of these interventions. Growth monitoring activities are part of the IMCI protocol that is to be followed during sick-child contacts, but implementation is poor[16] and their reach across the population is limited by the reach of public health facilities and by inclusion in sick child care rather than in well-child care.[42] This suggests the need for widely available services to deliver preventive nutrition services, not only to identify malnourished children, but to detect growth faltering early through regular growth monitoring and counseling for all children. Coverage can be increased by generating community awareness about the need for these services and generating demand. To ensure quality, trained staff should be available and have the necessary equipment to take anthropometric measurements, correctly use growth charts, and counsel caregivers. Measurement of care seeking among well children would help in monitoring progress in this area. Other elements of overall sick-child care quality should be improved by ensuring that staff are trained on IMCI, and that guidelines, equipment and medicine are available.

For peer review only - http://bmjopen.bmj.com/site/about/guidelines.xhtml

The inequality in adjusted coverage highlights targets for intervention according to administrative division and populations served. There is a clear variability in the adjusted coverage of indicators across administrative divisions, with some divisions (Khulna and Rangpur) performing better overall and others (Sylhet) performing poorly overall. For Sylhet specifically, poor performance may be because of its terrain, which makes many areas hard to reach. However, lessons should be shared from the successes of high-performing divisions such as Khulna and Rangpur, and focused strategic planning and action could help reduce regional disparities. Input-adjusted coverage also showed inequities by place of residence, education, and socioeconomic status. However, the size of these inequalities varied where the gaps were larger in the case of ANC and delivery, but smaller for child growth monitoring and sick-child care. Our findings of inequalities in coverage during pregnancy and delivery align with previous research finding that urban residence, higher education attainment (for the respondent and spouse), and higher wealth status significantly predict utilization of institutional delivery services[37] and 4+ ANC exposure[17]. Thus, there is a special need to specifically target underserved populations.

Investing in strengthening effective coverage of nutrition interventions is particularly important for a resource-poor country like Bangladesh which still carries a large burden of poor maternal and child health and nutrition. Despite consistent improvements in coverage of key nutrition service delivery platforms/contacts i.e. ANC and child health services, the health sector can do much more to strengthen nutrition service delivery in Bangladesh. The rapid growing private sector in the context of an ineffective regulatory environment has contributed to the lack of standardized practice in this setting [43]. Investing in the quality of public services,

For peer review only - http://bmjopen.bmj.com/site/about/guidelines.xhtml

therefore, could bring clients back to use public services and ensure better coverage of higher quality care. The challenge for governments scaling proven interventions at desired coverage *and* quality in such settings, however, is multidimensional. Depending on the type of contact and the intervention, it is likely that investments in strengthening the public sector platforms require both structural and process fixes. In concert with this, the government's stewardship in establishing functioning regulatory and accountability mechanisms for private sector service delivery can help to transform contact coverage into effective coverage.

Previous research has shown that relying only on facility-based health service delivery may not be appropriate for preventive service delivery in Bangladesh. In the current policy set-up, only a subset of children is reached by services provided during sick child visits and coverage of ANC for women continues to be sub-optimal [16]. To improve overall service coverage and quality, there is a need to strengthen existing services, while also exploring other avenues to improve access and coverage of key nutrition interventions through new service delivery contacts. Potential approaches include the provision of nutrition interventions in a preventative setting or via outreach activities, including home visits by frontline workers, community-based events for nutrition service interventions during pregnancy, or well-child services held at the community-level. Evidence exists about the use of community-based frontline workers to support infant and young child feeding practices and maternal nutrition in multiple countries including Bangladesh [44-47]. These, and other options, should continue to be explored in Bangladesh to ensure broad scale coverage of high quality nutrition interventions for women and children.

For peer review only - http://bmjopen.bmj.com/site/about/guidelines.xhtml

Our study has several strengths. Studies using data from household surveys often report contact coverage estimates that are limited by the respondent recall, but do not capture the content of these services nor their quality. Our study has reported both the services readiness (which was estimated from health facility data), and the contact coverage (which was drawn from household survey data) and combined these two to estimate input-adjusted coverage. The household surveys and facility data were collected in the same year, thus allowing us to compare facility readiness and services received at the same time for child health services. These data were combined using valid ecological linking method[28] by using facility type and disaggregating by divisions and urban/rural location. Variance and precision of coverage indicators were derived from linked data using the recommended delta method[29]. By including nutrition interventions for multiple populations (urban vs. rural; varying levels of education and wealth quintile) in our analysis, we provide a potential tool to assess the health promotion capacity of the health system by highlighting underserved populations.

We acknowledge some of the limitations of this analysis. *First,* although we included various facility levels, the exclusion of small private clinics or hospitals with less than 20 beds from the SPA could lead to an overestimation of quality for private sector users as larger facilities tend to have higher readiness than smaller ones. *Second,* contact coverage was estimated based on women's recall; however, women may not remember exactly where they received services, particularly if they went to more than one type of facility. We have addressed this by restricting the recall period of 3 years for ANC and birth to minimize the recall error. *Third*, the assumption of no systematic difference in facility readiness within region by the education or socioeconomic stratifies may affect inequality estimates. In reality, differences in

For peer review only - http://bmjopen.bmj.com/site/about/guidelines.xhtml

facilities in the same category, discrimination in provision care of underserved groups, and lack of individual capacity to extract value from the healthcare system may lead to systematic differences in quality experienced by women of different socioeconomic groups in the same tier of facilities,[48] leading to higher inequalities than are shown here. *Fourth,* the indicator used here will underestimate contact coverage of growth monitoring services if well children are frequently brought to health facilities for this purpose. Finally, although the SPA survey is quite comprehensive, it does not capture every aspect to measure facility readiness to provide nutrition interventions (such as the infrastructure to implement kangaroo mother care, calcium or food supplements for pregnant women, or IFA and food supplements for children), nor do the data available allow calculation of the care cascade from need for services through health benefits, including quality-adjusted coverage, user adherence -adjusted coverage, or outcome-adjusted coverage[5 9]. Additional data on beneficiary side such as client observations, exit interviews or client perceptions are needed to understand the flow of services provided, services received and whether they can translate into changes in behavior or practices.

CONCLUSION

Sustainable Development Goal 3 calls for achieving universal health coverage, which requires successfully covering underserved populations with healthcare services as well as ensuring that the quality of services is adequate. Nutrition interventions should be an integral part of health services for mothers and children; however, we find that nutrition input-adjusted coverage was suboptimal and varied sub-nationally and across the continuum of care in Bangladesh. Special efforts are needed to improve the reach as well as the quality of health and nutrition services to achieve the SDGs.

For peer review only - http://bmjopen.bmj.com/site/about/guidelines.xhtml

FIGURE LEGENDS

Figure 1: Readiness score of facilities to provide nutrition interventions, by division (A) and types of facility (B), Bangladesh 2014[1]

A. By division

B. By facility type

[1]ANC: antenatal care; DUPF: District and upazila public facilities; ULPF: Union level public facilities; PC: Public community clinic; NGO: Non-governmental organization

Figure 2: Regional readiness score of facilities to provide nutrition interventions versus coverage, Bangladesh 2014

Figure 3: Input-adjusted coverage of nutrition interventions by region, Bangladesh 2014

Figure 4: Inequality in input-adjusted coverage of nutrition interventions, Bangladesh 2014

SUPPLEMENTARY FILES

Supplementary table 1: Harmonized facility categories and reported categories in SPA and DHS

Supplemental Table 2: Percentage of health facilities with structural input items, by division

Supplemental Table 3: Percentage of health facilities with structural input items, by facility type

Supplemental Figure 1: Place of ANC and child care by facility type, Bangladesh 2014

Supplemental Figure 2: Distribution of facilities with ANC and sick child care by facility type, Bangladesh 2014

For peer review only - http://bmjopen.bmj.com/site/about/guidelines.xhtml

AUTHOR CONTRIBUTIONS

PHN conceived the manuscript, conducted the statistical analysis, wrote significant sections of the manuscript and revised the manuscript.

LQK conducted the statistical analysis, prepared tables and figures for the manuscript.

PP conducted the literature review, wrote significant sections of the manuscript and revised the manuscript.

MB supported data interpretation, reviewed and edited the manuscript.

PM supported data interpretation, reviewed and edited the manuscript.

EP supported data interpretation, reviewed and edited the manuscript.

HHL conceived the manuscript, advised on statistical analysis, supported data interpretation, and revised the manuscript.

All authors read and approved the final submitted manuscript.

FUNDING: Bill & Melinda Gates Foundation through POSHAN, led by International Food Policy Research Institute. Grant number: OPP50838

CONFLICT OF INTEREST STATEMENT*:* Authors declare no conflicts of interest.

DATA AVAILABILITY STATEMENT: All relevant data are within the manuscript and its Supporting Information files. Additional original data can be found at the DHS website https://dhsprogram.com/data/available-datasets.cfm.

For peer review only

ETHICAL APPROVAL

The survey and its procedures were properly reviewed and approved by the authority of the National Institute of Population Research and Training (NIPORT) of the Ministry of Health and Family Welfare. This study used secondary data analyses; thus, ethics approval was not required.

For peer review only

For peer review only - http://bmjopen.bmj.com/site/about/guidelines.xhtml

REFERENCES

1. Chou VB, Walker N, Kanyangarara M. Estimating the global impact of poor quality of care on maternal and neonatal outcomes in 81 low- and middle-income countries: A modeling study. *PLoS Med* 2019;16(12):e1002990. doi: 10.1371/journal.pmed.1002990 [published Online First: 2019/12/19]
2. Bhutta ZA, Das JK, Rizvi A, et al. Evidence-based interventions for improvement of maternal and child nutrition: what can be done and at what cost? *Lancet* 2013;382(9890):452-77. doi: 10.1016/S0140-6736(13)60996-4 [published Online First: 2013/06/12]
3. Gillespie S, Menon P, Heidkamp R, et al. Measuring the coverage of nutrition interventions along the continuum of care: time to act at scale. *BMJ Glob Health* 2019;4(Suppl 4):e001290. doi: 10.1136/bmjgh-2018-001290 [published Online First: 2019/07/13]
4. Countdown to 2030 Collaboration. Countdown to 2030: tracking progress towards universal coverage for reproductive, maternal, newborn, and child health. *Lancet* 2018;391(10129):1538-48. doi: 10.1016/S0140-6736(18)30104-1 [published Online First: 2018/02/06]
5. Amouzou A, Leslie HH, Ram M, et al. Advances in the measurement of coverage for RMNCH and nutrition: from contact to effective coverage. *BMJ Glob Health* 2019;4(Suppl 4):e001297. doi: 10.1136/bmjgh-2018-001297 [published Online First: 2019/07/13]
6. Shengelia B, Tandon A, Adams OB, et al. Access, utilization, quality, and effective coverage: an integrated conceptual framework and measurement strategy. *Soc Sci Med* 2005;61(1):97-109. doi: 10.1016/j.socscimed.2004.11.055 [published Online First: 2005/04/26]
7. Boerma T, AbouZahr C, Evans D, et al. Monitoring intervention coverage in the context of universal health coverage. *PLoS Med* 2014;11(9):e1001728. doi: 10.1371/journal.pmed.1001728 [published Online First: 2014/09/23]
8. Donabedian A. Evaluating the quality of medical care. 1966. *Milbank Q* 2005;83(4):691-729. doi: 10.1111/j.1468-0009.2005.00397.x [published Online First: 2005/11/11]
9. Marsh AD, Muzigaba M, Diaz T, et al. Effective coverage measurement in maternal, newborn,child, and adolescent health and nutrition: progress, future prospects, and implications for quality health systems. *Lancet Glob Health* 2020;8:e730–36.
10. Kanyangarara M, Munos MK, Walker N. Quality of antenatal care service provision in health facilities across sub-Saharan Africa: Evidence from nationally representative health facility assessments. *J Glob Health* 2017;7(2):021101. doi: 10.7189/jogh.07.021101 [published Online First: 2017/11/23]
11. Leslie HH, Malata A, Ndiaye Y, et al. Effective coverage of primary care services in eight high-mortality countries. *BMJ Glob Health* 2017;2(3):e000424. doi: 10.1136/bmjgh-2017-000424 [published Online First: 2018/04/11]
12. Kanyangarara M, Chou VB, Creanga AA, et al. Linking household and health facility surveys to assess obstetric service availability, readiness and coverage: evidence from 17 low- and middle-income countries. *J Glob Health* 2018;8(1):010603. doi: 10.7189/jogh.08.010603 [published Online First: 2018/06/05]
13. Wang W, Mallick L, Allen C, et al. Effective coverage of facility delivery in Bangladesh, Haiti, Malawi, Nepal, Senegal, and Tanzania. *PLoS One* 2019;14(6):e0217853. doi: 10.1371/journal.pone.0217853 [published Online First: 2019/06/12]
14. Joseph NT, Piwoz E, Lee D, et al. Examining coverage, content, and impact of maternal nutrition interventions: the case for quality-adjusted coverage measurement. *Journal of Global Health* 2020;10: 010501
15. NIPORT. Bangladesh Demographic and Health Survey 2017-18: Key Indicators. Dhaka, Bangladesh, and Rockville, Maryland, USA: NIPORT and ICF, 2019.

For peer review only - http://bmjopen.bmj.com/site/about/guidelines.xhtml

16. Billah SM, Saha KK, Khan ANS, et al. Quality of nutrition services in primary health care facilities: Implications for integrating nutrition into the health system in Bangladesh. *PLoS One* 2017;12(5):e0178121. doi: 10.1371/journal.pone.0178121 [published Online First: 2017/05/26]
17. Islam MM, Masud MS. Health care seeking behaviour during pregnancy, delivery and the postnatal period in Bangladesh: Assessing the compliance with WHO recommendations. *Midwifery* 2018;63:8-16. doi: 10.1016/j.midw.2018.04.021 [published Online First: 2018/05/15]
18. Ali N, Sultana M, Sheikh N, et al. Predictors of Optimal Antenatal Care Service Utilization Among Adolescents and Adult Women in Bangladesh. *Health Serv Res Manag Epidemiol* 2018;5:2333392818781729. doi: 10.1177/2333392818781729 [published Online First: 2018/08/08]
19. Anwar I, Nababan HY, Mostari S, et al. Trends and inequities in use of maternal health care services in Bangladesh, 1991-2011. *PLoS One* 2015;10(3):e0120309. doi: 10.1371/journal.pone.0120309 [published Online First: 2015/03/24]
20. Biswas TK, Sujon H, Rahman MH, et al. Quality of maternal and newborn healthcare services in two public hospitals of Bangladesh: identifying gaps and provisions for improvement. *BMC Pregnancy Childbirth* 2019;19(1):488. doi: 10.1186/s12884-019-2656-1 [published Online First: 2019/12/12]
21. NIPORT. National Institute of Population Research and Training (NIPORT), Mitra and Associates, and ICF International. Bangladesh Demographic and Health Survey 2014. Dhaka, Bangladesh, and Rockville, Maryland, USA: NIPORT, Mitra and Associates, and ICF International, 2016.
22. NIPORT. National Institute of Population Research and Training (NIPORT), Associates for Community and Population Research (ACPR), and ICF International. 2016. Bangladesh Health Facility Survey 2014. Dhaka, Bangladesh: NIPORT, ACPR, and ICF International, 2016.
23. DHS. The DHS Program. SPA overview. https://dhsprogram.com/What-We-Do/Survey-Types/SPA-Questionnaires.cfm. 2020
24. Arifeen SE, Hoque DM, Akter T, et al. Effect of the Integrated Management of Childhood Illness strategy on childhood mortality and nutrition in a rural area in Bangladesh: a cluster randomised trial. *Lancet* 2009;374(9687):393-403. doi: 10.1016/S0140-6736(09)60828-X [published Online First: 2009/08/04]
25. WHO. Service Availability and Readiness Assessment (SARA). An annual monitoring system for service delivery. Reference Manual. Geneva, Switzerland: World Health Organization 2013.
26. Sheffel A, Zeger S, Heidkamp R, et al. Development of summary indices of antenatal care service quality in Haiti, Malawi and Tanzania. *BMJ Open* 2019;9(12):e032558. doi: 10.1136/bmjopen-2019-032558 [published Online First: 2019/12/05]
27. Mallick L, Temsah G, Wang W. Comparing summary measures of quality of care for family planning in Haiti, Malawi, and Tanzania. *PLoS One* 2019;14(6):e0217547. doi: 10.1371/journal.pone.0217547 [published Online First: 2019/06/08]
28. Munos MK, Maiga A, Do M, et al. Linking household survey and health facility data for effective coverage measures: a comparison of ecological and individual linking methods using the Multiple Indicator Cluster Survey in Cote d'Ivoire. *J Glob Health* 2018;8(2):020803. doi: 10.7189/jogh.08.020803 [published Online First: 2018/11/10]
29. Sauer SM, Pullum T, Wang W, et al. Variance estimation for effective coverage measures: A simulation study. *Journal of Glob Health (in press)* 2020
30. UNDP. Sustainable Development Goals Geneva, Switzerland: United Nations; 2015 [Available from: https://sustainabledevelopment.un.org/sdg3 accessed 12/03 2020.
31. Simkhada B, Teijlingen ER, Porter M, et al. Factors affecting the utilization of antenatal care in developing countries: systematic review of the literature. *J Adv Nurs* 2008;61(3):244-60. doi: 10.1111/j.1365-2648.2007.04532.x [published Online First: 2008/01/17]

For peer review only - http://bmjopen.bmj.com/site/about/guidelines.xhtml

32. Haider MR, Qureshi ZP, Khan MM. Effects of women's autonomy on maternal healthcare utilization in Bangladesh: Evidence from a national survey. *Sex Reprod Healthc* 2017;14:40-47. doi: 10.1016/j.srhc.2017.09.002 [published Online First: 2017/12/03]
33. Haque MA, Dash SK, Chowdhury MA. Maternal health care seeking behavior: the case of Haor (wetland) in Bangladesh. *BMC Public Health* 2016;16:592. doi: 10.1186/s12889-016-3296-2 [published Online First: 2016/07/20]
34. Islam MR, Odland JO. Determinants of antenatal and postnatal care visits among Indigenous people in Bangladesh: a study of the Mru community. *Rural Remote Health* 2011;11(2):1672. [published Online First: 2011/07/01]
35. Watterson JL, Walsh J, Madeka I. Using mHealth to Improve Usage of Antenatal Care, Postnatal Care, and Immunization: A Systematic Review of the Literature. *Biomed Res Int* 2015;2015:153402. doi: 10.1155/2015/153402 [published Online First: 2015/09/18]
36. NIPORT. National Institute of Population Research and Training (NIPORT), Mitra and Associates, and ICF International. Bangladesh Demographic and Health Survey 2011. Dhaka, Bangladesh, and Rockville, Maryland, USA: NIPORT, Mitra and Associates, and ICF International, 2013.
37. Yaya S, Bishwajit G, Ekholuenetale M. Factors associated with the utilization of institutional delivery services in Bangladesh. *PLoS One* 2017;12(2):e0171573. doi: 10.1371/journal.pone.0171573 [published Online First: 2017/02/14]
38. Sharma BB, Jones L, Loxton DJ, et al. Systematic review of community participation interventions to improve maternal health outcomes in rural South Asia. *BMC Pregnancy Childbirth* 2018;18(1):327. doi: 10.1186/s12884-018-1964-1 [published Online First: 2018/08/12]
39. Lassi ZS, Bhutta ZA. Community-based intervention packages for reducing maternal and neonatal morbidity and mortality and improving neonatal outcomes. *Cochrane Database Syst Rev* 2015(3):CD007754. doi: 10.1002/14651858.CD007754.pub3 [published Online First: 2015/03/25]
40. NIPORT. National Institute of Population Research and Training (NIPORT), Associates for Community and Population Research (ACPR), and ICF International. 2020. Bangladesh Health Facility Survey 2017. Dhaka, Bangladesh: NIPORT, ACPR, and ICF International, 2020.
41. MoH&FW. Operational Plan For 'National Nutrition Services' July 2011-June 2016. In: Welfare MoHaF, ed., 2011.
42. Saha KK, Billah SM, Menon P, et al. Bangladesh National Nutrition Services: assessment of implementation status: The World Bank, 2015.
43. Ahmed SM, Evans TG, Standing H, et al. Harnessing pluralism for better health in Bangladesh. *Lancet* 2013;382(9906):1746-55. doi: 10.1016/S0140-6736(13)62147-9 [published Online First: 2013/11/26]
44. Kim SS, Nguyen PH, Tran LM, et al. Large-Scale Social and Behavior Change Communication Interventions Have Sustained Impacts on Infant and Young Child Feeding Knowledge and Practices: Results of a 2-Year Follow-Up Study in Bangladesh. *J Nutr* 2018;148(10):1605-14. doi: 10.1093/jn/nxy147 [published Online First: 2018/09/01]
45. Menon P, Nguyen PH, Saha KK, et al. Combining Intensive Counseling by Frontline Workers with a Nationwide Mass Media Campaign Has Large Differential Impacts on Complementary Feeding Practices but Not on Child Growth: Results of a Cluster-Randomized Program Evaluation in Bangladesh. *J Nutr* 2016;146(10):2075-84. doi: 10.3945/jn.116.232314
46. Menon P, Nguyen PH, Saha KK, et al. Impacts on Breastfeeding Practices of At-Scale Strategies That Combine Intensive Interpersonal Counseling, Mass Media, and Community Mobilization: Results of Cluster-Randomized Program Evaluations in Bangladesh and Viet Nam. *PLoS Med* 2016;13(10):e1002159. doi: 10.1371/journal.pmed.1002159

For peer review only

For peer review only - http://bmjopen.bmj.com/site/about/guidelines.xhtml

47. Nguyen PH, Kim SS, Sanghvi T, et al. Integrating Nutrition Interventions into an Existing Maternal, Neonatal, and Child Health Program Increased Maternal Dietary Diversity, Micronutrient Intake, and Exclusive Breastfeeding Practices in Bangladesh: Results of a Cluster-Randomized Program Evaluation. *J Nutr* 2017;147(12):2326-37. doi: 10.3945/jn.117.257303 [published Online First: 2017/10/13]

48. Sharma J, Leslie HH, Kundu F, et al. Poor Quality for Poor Women? Inequities in the Quality of Antenatal and Delivery Care in Kenya. *PLoS One* 2017;12(1):e0171236. doi: 10.1371/journal.pone.0171236 [published Online First: 2017/02/01]

For peer review only

For peer review only - http://bmjopen.bmj.com/site/about/guidelines.xhtml

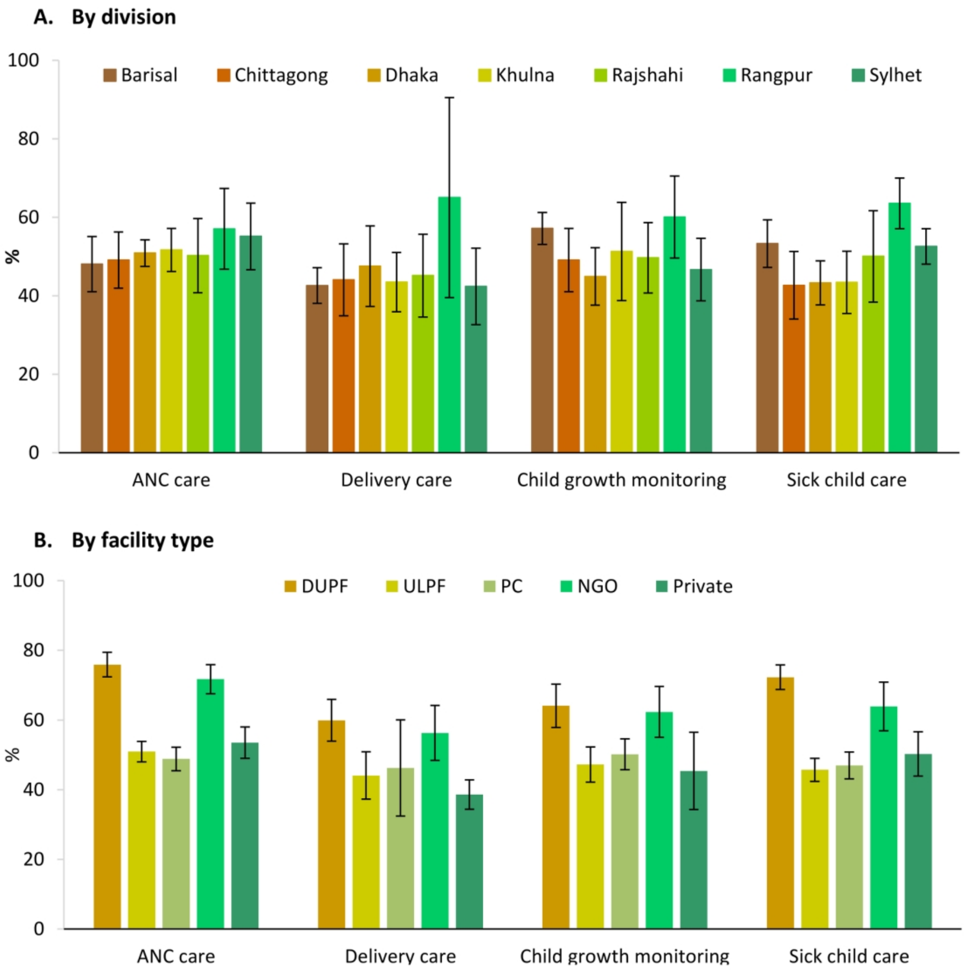

167x163mm (300 x 300 DPI)

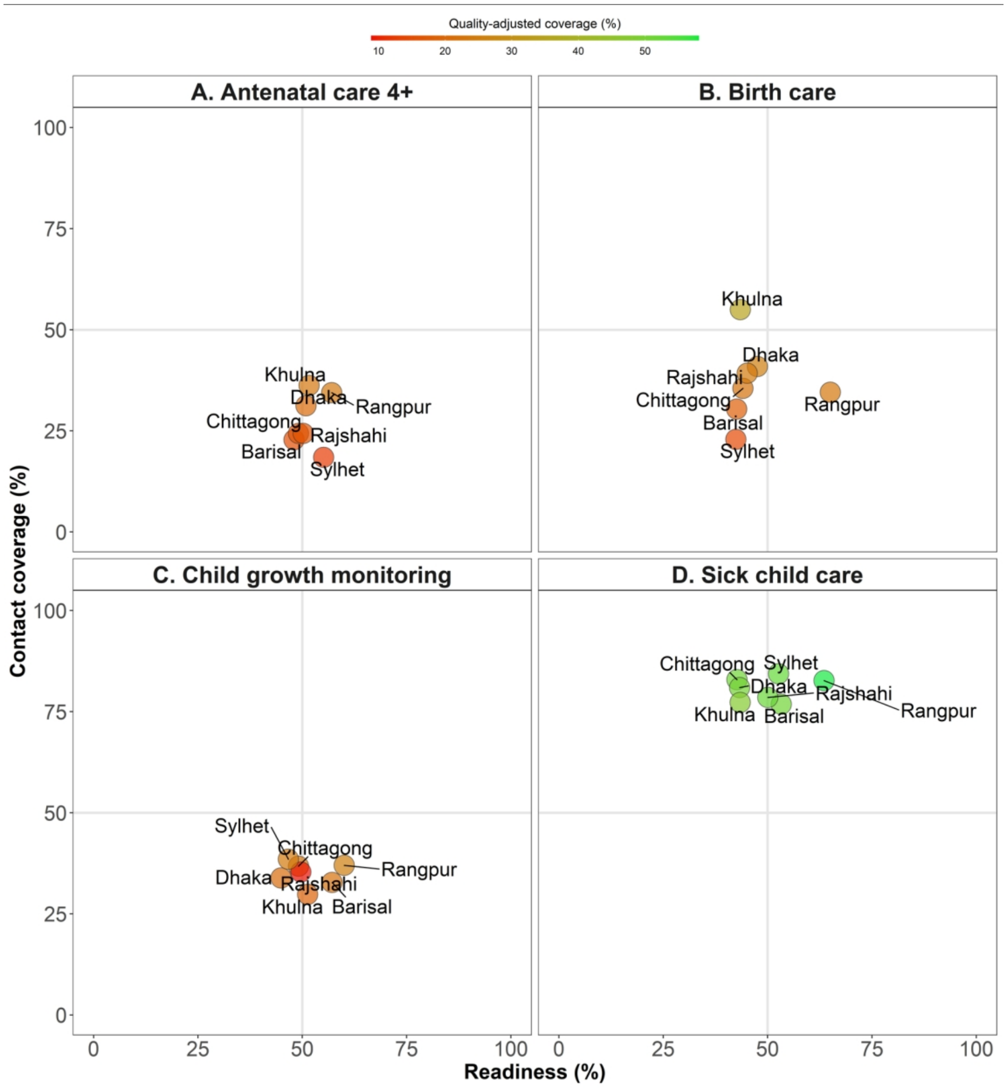

152x165mm (300 x 300 DPI)

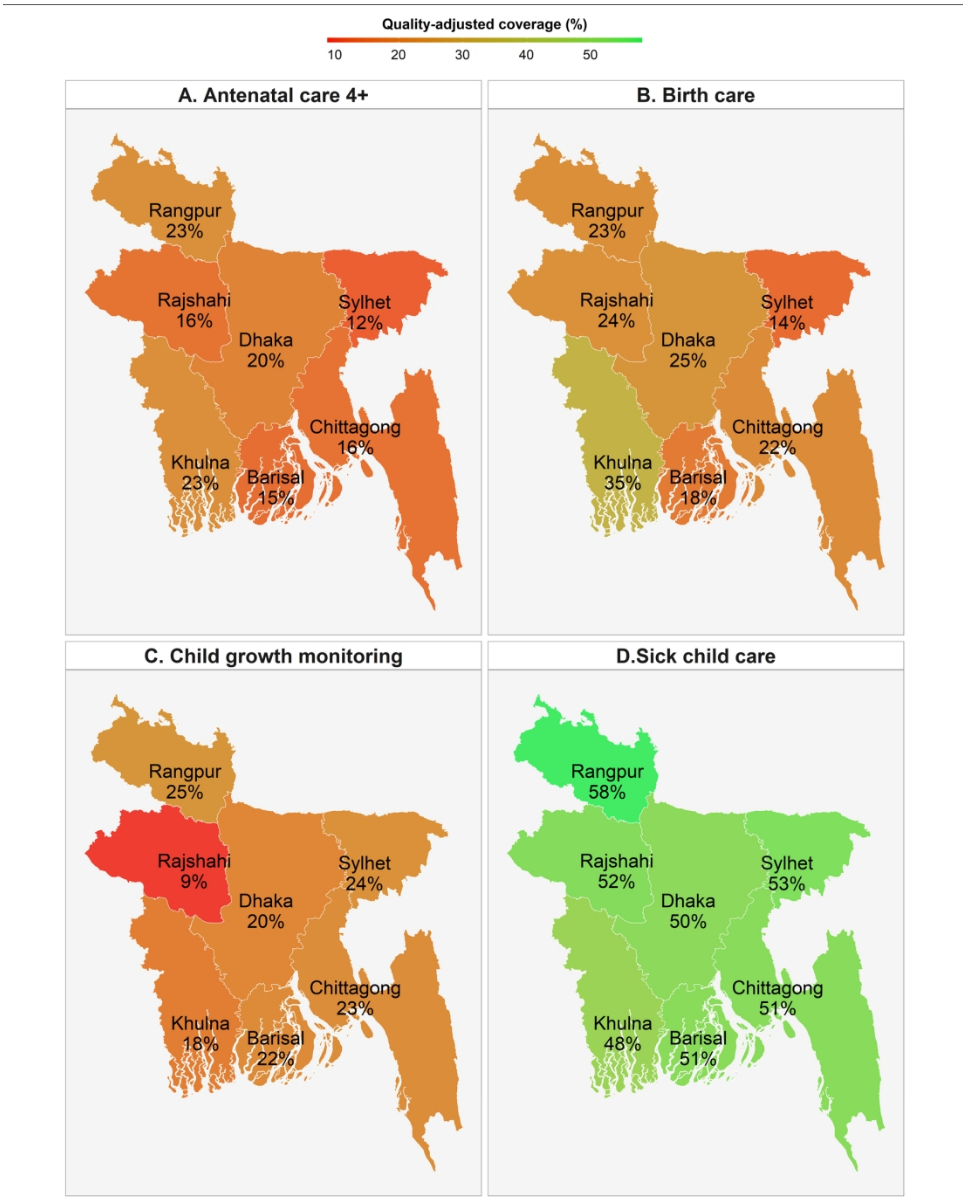

152x190mm (300 x 300 DPI)

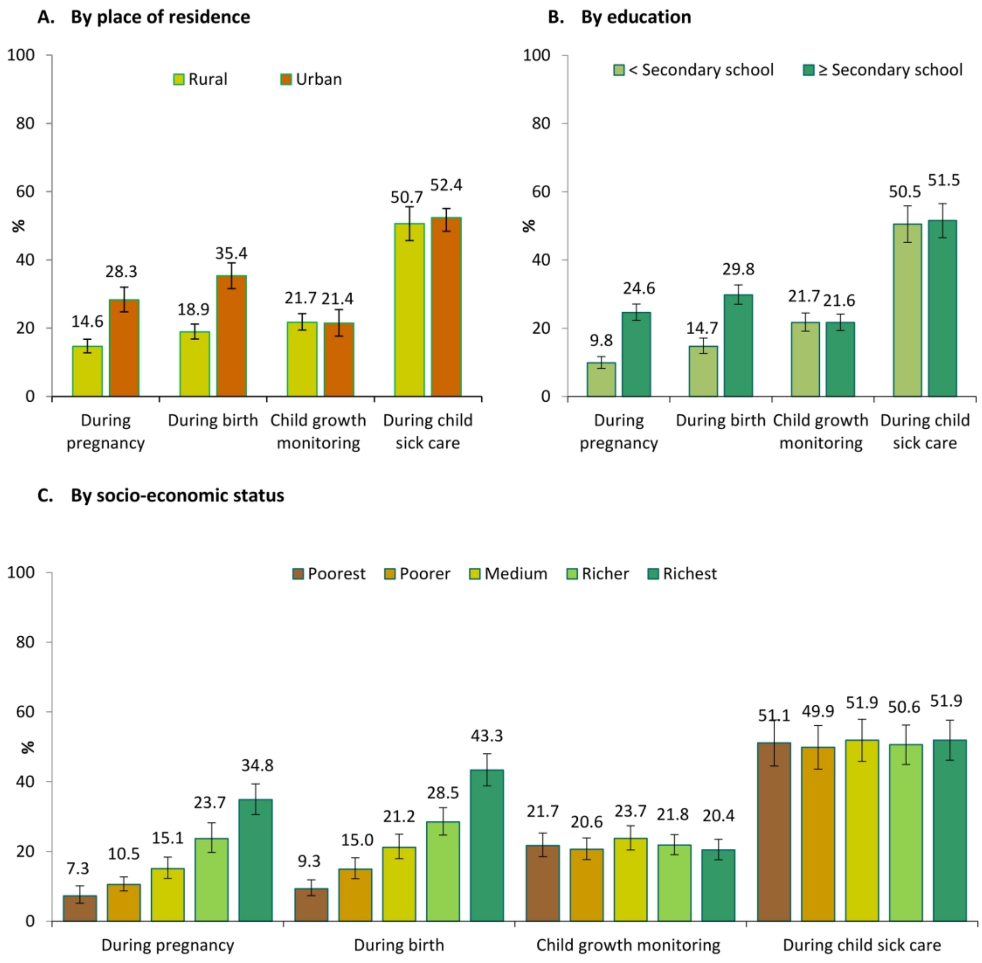

190x184mm (300 x 300 DPI)

Supplemental Table 1. Harmonized facility categories and reported categories in SPA and DHS

Harmonized facility category	SPA facility category	DHS facility category
District and upazila public facilities	District hospital (DH)<br>Upazila health complex (UHC)<br>Maternal and child welfare center (MCWC)	District hospital (DH)<br>Upazila health complex (UHC)<br>Maternal and child welfare center (MCWC)
Union level public facilities	Union health and family welfare center (UHFWC)/(UHFWC upgraded)<br>Union subcenter/rural dispensary	Upazila health & family welfare center<br>Satellite clinic/epi outreach
Public community clinic	Public community clinic	Public community clinic<br>Other public sectors
NGO clinic/hospital	NGO clinic/hospital	NGO static clinic<br>NGO satellite clinic<br>Other NGO clinic
Private clinic/hospital	Private clinic/hospital	Private clinic/hospital<br>Private medical<br>Traditional/Qualified doctor

For peer review only

Supplemental Table 2: Percentage of health facilities with structural input items, by division

		Barisal	Chittagong	Dhaka	Khulna	Rajshahi	Rangpur	Sylhet
**Antenatal care**								
**Staff**	Staff with any training on ANC	38.55	57.24	57.8	44.27	35.87	39.45	54.35
**Guideline**	National guidelines for ANC	56.26	38.49	39.93	58.47	46.86	70.65	60.09
	Visual aids for client education	69.80	63.78	57.09	72.27	67.74	72.19	91.51
**Functional equipment**	Adult weighing scale	88.74	89.73	72.93	91.45	87.21	93.23	90.38
	Tape measure for fundal height	41.18	27.51	33.42	40.77	32.44	50.37	36.35
	Blood pressure apparatus	87.03	86.88	90.67	94.51	92.03	97.85	88.96
	Stethoscope	89.75	86.32	92.10	96.73	93.69	96.06	87.98
	Fetal stethoscope	13.36	7.84	15.89	13.24	8.39	29.11	26.26
**Diagnostics capacity**	Hemoglobin	5.91	8.68	12.46	3.71	9.29	19.29	11.60
	Urine protein	8.28	15.84	20.06	11.35	18.58	26.71	18.58
**Medicine**	Iron tablets	41.31	50.18	64.42	64.36	73.60	65.79	42.02
	IFA tablets	93.74	80.16	73.44	83.17	89.06	90.34	86.82
**Birth care**								
**Staff**	Staff with any training on IMPACT	23.68	33.01	45.01	37.02	35.94	43.94	34.23
**Guideline**	Guidelines for BEmOC or CEmOC	32.66	26.00	25.83	21.19	10.84	56.25	20.46
**Functional equipment**	Infant scale	52.46	56.88	49.54	61.50	77.21	89.71	52.01
	Manual or digital BP apparatus	90.45	89.58	94.13	82.86	100.00	100.00	92.91
**Child growth monitoring**								
**Staff**	Staff with any training on growth monitoring	50.08	46.13	55.37	47.18	50.20	40.08	49.72
**Guideline**	Guidelines for growth monitoring	57.50	48.33	37.09	51.80	41.36	65.11	22.03
**Functional equipment**	Child scale (child or infant)	69.87	67.66	50.28	60.79	75.85	96.16	82.95
	Length or height board	70.34	49.76	50.48	56.34	66.41	69.23	72.63
	Tape for measuring head	32.28	24.25	23.24	30.86	27.20	43.00	50.57
	Growth chart	83.21	69.66	45.23	71.65	60.51	91.50	66.73
Readiness score for child growth monitoring								
**Sick child care**								
**Staff**	Staff with any training on IMCI	67.19	48.62	57.54	42.75	52.18	55.86	58.89
**Guideline**	Guidelines for IMCI	66.62	42.83	38.33	51.17	45.03	77.60	58.55
	IMCI chart booklet	64.33	40.94	49.47	34.79	55.25	84.87	57.20
	IMCI mother's cards (IMCI card)	43.90	32.20	31.08	25.35	32.13	83.08	38.93
	Other visual aids for teaching caretakers	64.14	46.00	43.75	53.46	60.44	80.14	71.73
**Functional equipment**	Child scale (child or infant)	66.12	57.20	40.63	64.15	64.93	84.61	79.74
**Diagnostics capacity**	Hemoglobin	5.15	8.29	11.85	3.45	9.50	19.83	11.63
**Medicine**	ORS	71.97	63.52	61.01	51.95	74.91	69.94	52.35
	Albendazole/ Mebendazole	91.78	87.03	89.88	85.69	89.78	96.99	97.72
	Iron tablet	35.08	43.04	57.41	60.68	72.61	58.15	33.61
	Vitamin A	67.99	54.26	67.73	62.17	76.47	81.77	47.22
	Zinc tablet/ Zinc sulphate syrup	76.46	56.77	69.70	61.62	71.29	76.64	62.32

ANC: antenatal care, BEmOC: Basic Emergency Obstetric and Neonatal Care, CEmOC: Comprehensive Emergency Obstetric and Neonatal Care, IMCI: integrated management of childhood illness, IMPAC: integrated management of pregnancy and childbirth, ORS: Oral rehydration salts.

For peer review only

Supplemental Table 3: Percentage of health facilities with structural input items, by facility type

		DUPF	ULPF	PC	NGO	Private
**Antenatal care**						
**Staff**	Staff with any training on ANC	83.64	50.04	46.31	60.36	27.48
**Guideline**	National or other guidelines for ANC	70.33	47.82	48.16	72.95	24.70
	Visual aids for client education	79.25	68.52	65.47	78.34	45.94
**Equipment**	Adult weighing scale	91.47	82.20	85.67	94.15	83.82
	Tape measure for fundal height	60.76	35.40	32.67	63.31	52.64
	Blood pressure apparatus	99.12	90.74	90.30	99.14	94.67
	Stethoscope	98.94	91.02	91.18	99.15	97.97
	Fetal stethoscope	46.35	20.38	8.67	46.55	43.29
**Diagnostics capacity**	Hemoglobin	61.97	7.90	3.53	58.87	72.14
	Urine protein	63.89	12.02	12.34	68.63	68.12
**Medicine**	Iron tablets	69.37	62.22	57.81	73.95	57.60
	IFA tablets	88.23	82.69	84.79	82.66	62.35
**Birth care**						
**Staff**	Staff with any training on IMPACT	55.96	31.75	40.71	52.84	22.05
**Guideline**	Guidelines for BEmOC or CEmOC	40.50	26.09	27.42	27.94	11.29
**Equipment**	Infant scale	69.52	60.47	47.87	76.72	64.97
	Manual or digital BP apparatus	96.91	88.33	93.18	99.34	100.00
**Child growth monitoring**						
**Staff**	Staff with any training on growth monitoring	67.88	42.91	50.18	47.73	40.35
**Guideline**	Guidelines for growth monitoring	52.55	43.30	46.22	64.57	29.31
**Equipment**	Child scale (child or infant)	82.28	74.50	64.19	86.20	90.63
	Tape for measuring head	47.65	26.41	28.95	57.15	32.90
	Length or height board	71.77	52.26	60.09	71.51	57.32
	Growth chart	85.51	68.72	63.01	83.71	85.13
Readiness score for child growth monitoring						
**Sick child care**						
**Staff**	Staff with any training on IMCI	78.46	52.70	54.11	45.91	32.97
**Guideline**	Guidelines for IMCI	76.74	55.46	47.93	56.32	30.23
	IMCI chart booklet	78.06	59.78	49.35	64.58	35.14
	IMCI mother's cards (IMCI card)	62.65	50.15	33.78	52.56	24.58
	Other visual aids for teaching caretakers	65.70	54.39	55.88	68.49	41.49
**Equipment**	Child scale (child or infant)	84.76	66.10	55.77	80.08	77.59
**Diagnostics capacity**	Hemoglobin	62.53	8.18	3.78	58.33	68.26
**Medicine**	ORS	64.45	34.50	73.57	78.80	46.65
	Albendazole/ Mebendazole	75.39	87.03	93.31	83.53	79.06
	Iron tablet	64.20	53.69	52.45	73.53	59.43
	Vitamin A	58.72	29.90	80.73	70.19	53.41
	Zinc tablet/ Zinc sulphate syrup	65.12	34.36	79.26	73.28	80.94

DUPF: District and upazila public facilities, ULPF: Union level public facilities Public community clinic
ANC: antenatal care, BEmOC: Basic Emergency Obstetric and Neonatal Care, CEmOC: Comprehensive Emergency Obstetric and Neonatal Care, IMCI: integrated management of childhood illness, IMPAC: integrated management of pregnancy and childbirth, ORS: Oral rehydration salts.

For peer review only

Supplemental Figure 1: Place of ANC and child care by facility type, Bangladesh 2014

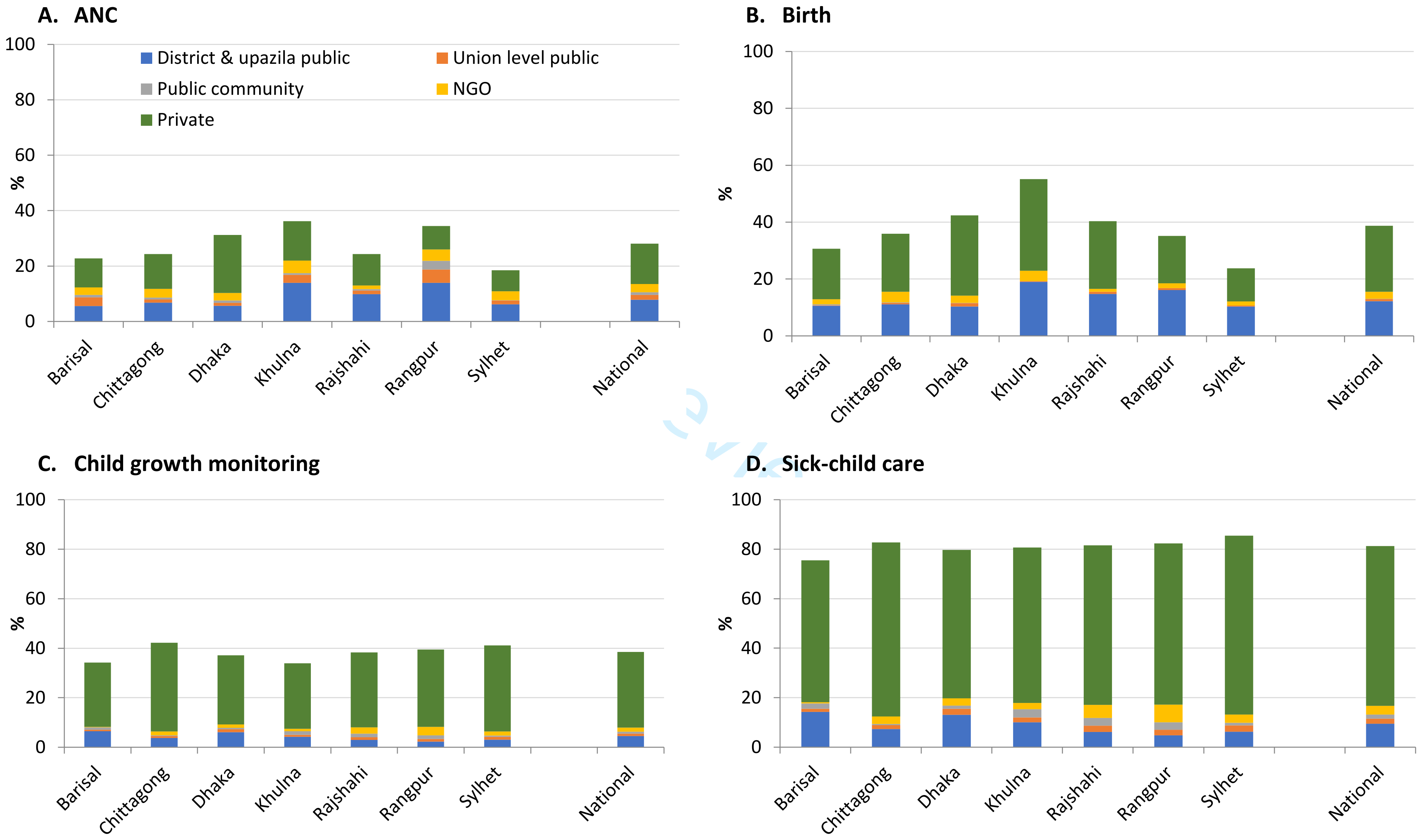

Source: DHS data

Supplemental Figure 2: Distribution of facilities with ANC and sick child care by facility type, Bangladesh 2014

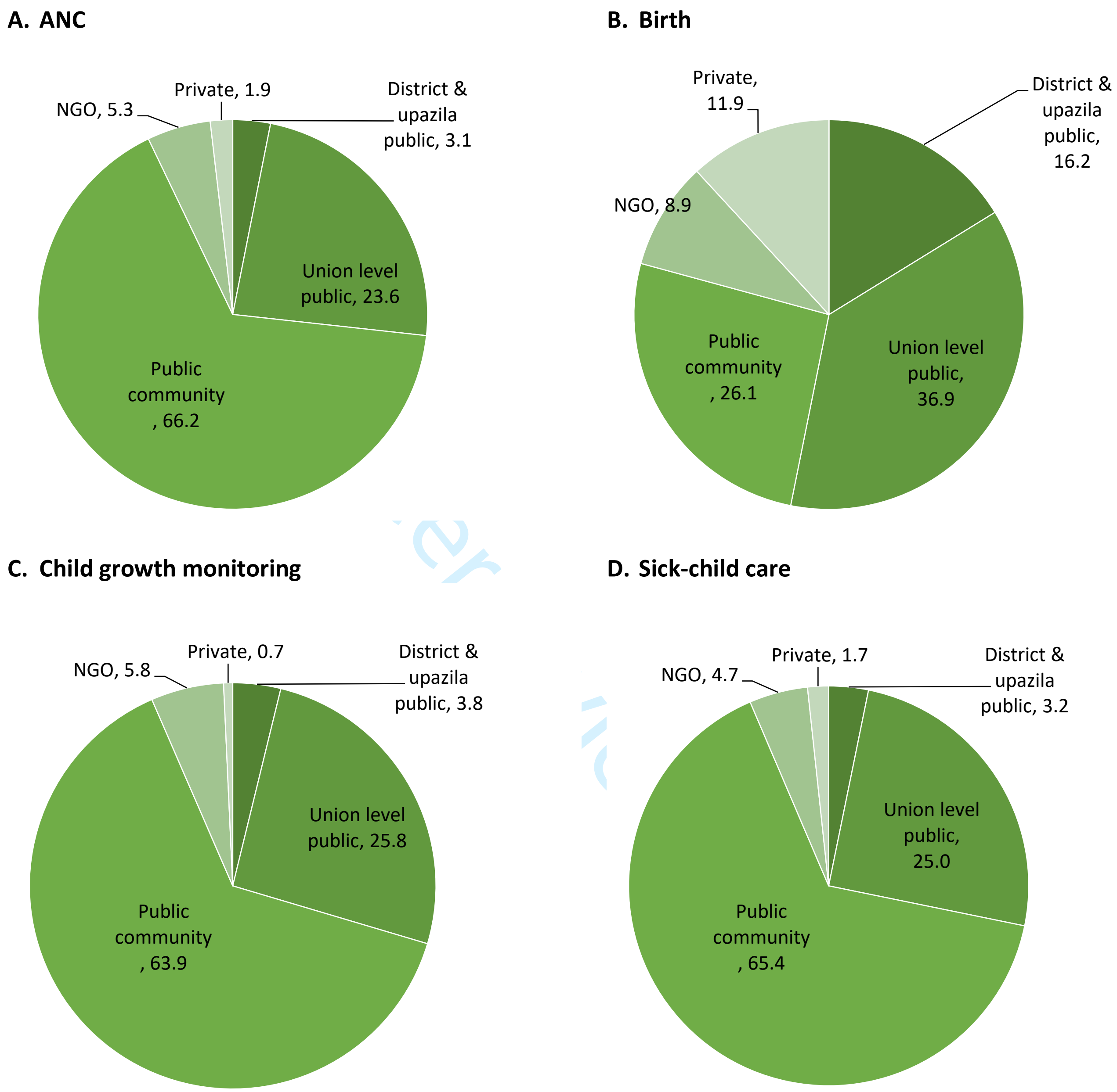

Source: SPA data

For peer review only

STROBE Statement—Checklist of items that should be included in reports of cross-sectional studies

	Item No	Recommendation	Page number from manuscript
**Title and abstract**	1	(*a*) Indicate the study's design with a commonly used term in the title or the abstract	1, 3
		(*b*) Provide in the abstract an informative and balanced summary of what was done and what was found	3-4
**Introduction**			
Background/rationale	2	Explain the scientific background and rationale for the investigation being reported	6-8
Objectives	3	State specific objectives, including any prespecified hypotheses	8
**Methods**			
Study design	4	Present key elements of study design early in the paper	9-10
Setting	5	Describe the setting, locations, and relevant dates, including periods of recruitment, exposure, follow-up, and data collection	9-10
Participants	6	(*a*) Give the eligibility criteria, and the sources and methods of selection of participants	9-10
Variables	7	Clearly define all outcomes, exposures, predictors, potential confounders, and effect modifiers. Give diagnostic criteria, if applicable	10-12
Data sources/ measurement	8*	For each variable of interest, give sources of data and details of methods of assessment (measurement). Describe comparability of assessment methods if there is more than one group	10-12
Bias	9	Describe any efforts to address potential sources of bias	NA
Study size	10	Explain how the study size was arrived at	NA
Quantitative variables	11	Explain how quantitative variables were handled in the analyses. If applicable, describe which groupings were chosen and why	13-15
Statistical methods	12	(*a*) Describe all statistical methods, including those used to control for confounding	13-15
		(*b*) Describe any methods used to examine subgroups and interactions	13-15
		(*c*) Explain how missing data were addressed	NA
		(*d*) If applicable, describe analytical methods taking account of sampling strategy	13-15
		(*e*) Describe any sensitivity analyses	NA
**Results**			
Participants	13*	(a) Report numbers of individuals at each stage of study—eg numbers potentially eligible, examined for eligibility, confirmed eligible, included in the study, completing follow-up, and analysed	15
		(b) Give reasons for non-participation at each stage	NA
		(c) Consider use of a flow diagram	NA
Descriptive data	14*	(a) Give characteristics of study participants (eg demographic, clinical, social) and information on exposures and potential	15

		confounders	
		(b) Indicate number of participants with missing data for each variable of interest	NA
Outcome data	15*	Report numbers of outcome events or summary measures	15-20
Main results	16	(*a*) Give unadjusted estimates and, if applicable, confounder-adjusted estimates and their precision (eg, 95% confidence interval). Make clear which confounders were adjusted for and why they were included	15-20
		(*b*) Report category boundaries when continuous variables were categorized	NA
		(*c*) If relevant, consider translating estimates of relative risk into absolute risk for a meaningful time period	NA
Other analyses	17	Report other analyses done—eg analyses of subgroups and interactions, and sensitivity analyses	NA
**Discussion**			
Key results	18	Summarise key results with reference to study objectives	21
Limitations	19	Discuss limitations of the study, taking into account sources of potential bias or imprecision. Discuss both direction and magnitude of any potential bias	26-27
Interpretation	20	Give a cautious overall interpretation of results considering objectives, limitations, multiplicity of analyses, results from similar studies, and other relevant evidence	21-24
Generalisability	21	Discuss the generalisability (external validity) of the study results	25
**Other information**			
Funding	22	Give the source of funding and the role of the funders for the present study and, if applicable, for the original study on which the present article is based	29

*Give information separately for exposed and unexposed groups.

Note: An Explanation and Elaboration article discusses each checklist item and gives methodological background and published examples of transparent reporting. The STROBE checklist is best used in conjunction with this article (freely available on the Web sites of PLoS Medicine at http://www.plosmedicine.org/, Annals of Internal Medicine at http://www.annals.org/, and Epidemiology at http://www.epidem.com/). Information on the STROBE Initiative is available at www.strobe-statement.org.

For peer review only
